# Improving adenine and dual base editors through introduction of TadA-8e and Rad51DBD

**DOI:** 10.1038/s41467-023-36887-1

**Published:** 2023-03-03

**Authors:** Niannian Xue, Xu Liu, Dan Zhang, Youming Wu, Yi Zhong, Jinxin Wang, Wenjing Fan, Haixia Jiang, Biyun Zhu, Xiyu Ge, Rachel V. L. Gonzalez, Liang Chen, Shun Zhang, Peilu She, Zhilin Zhong, Jianjian Sun, Xi Chen, Liren Wang, Zhimin Gu, Ping Zhu, Mingyao Liu, Dali Li, Tao P. Zhong, Xiaohui Zhang

**Affiliations:** 1 Shanghai Frontiers Science Center of Genome Editing and Cell Therapy, Shanghai Key Laboratory of Regulatory Biology, Institute of Biomedical Sciences and School of Life Sciences, East China Normal University, Shanghai, China; 2 Institute of Systems Medicine, Chinese Academy of Medical Sciences & Peking Union Medical College, Beijing, China; 3 Suzhou Institute of Systems Medicine, Suzhou, China; 4 School of Life Sciences & Biotechnology, Shanghai Jiao Tong University, Shanghai, China; 5 Department of Molecular and Integrative Physiology, University of Illinois at Urbana-Champaign, Urbana, IL USA; 6 Department of Physiology and Cellular Biophysics, Columbia University, Manhattan, NY USA; 7 BRL Medicine, Inc., Shanghai, China; 8 Guangdong Cardiovascular Institute, Guangdong Provincial People’s Hospital, Guangdong Academy of Medical Sciences, Guangzhou, China

**Keywords:** CRISPR-Cas9 genome editing, Synthetic biology

## Abstract

Base editors, including dual base editors, are innovative techniques for efficient base conversions in genomic DNA. However, the low efficiency of A-to-G base conversion at positions proximal to the protospacer adjacent motif (PAM) and the A/C simultaneous conversion of the dual base editor hinder their broad applications. In this study, through fusion of ABE8e with Rad51 DNA-binding domain, we generate a hyperactive ABE (hyABE) which offers improved A-to-G editing efficiency at the region (A_10_-A_15_) proximal to the PAM, with 1.2- to 7-fold improvement compared to ABE8e. Similarly, we develop optimized dual base editors (eA&C-BEmax and hyA&C-BEmax) with markedly improved simultaneous A/C conversion efficiency (1.2-fold and 1.5-fold improvement, respectively) compared to A&C-BEmax in human cells. Moreover, these optimized base editors catalyze efficiently nucleotide conversions in zebrafish embryos to mirror human syndrome or in human cells to potentially treat genetic diseases, indicating their great potential in broad applications for disease modeling and gene therapy.

## Abstract (web summary)

There is a low efficiency of A-to-G base conversion in at specific positions using base editors. Here the authors fuse ABE8e with the Rad51 DNA-binding domain to generate a hyperactive ABE allowing improved A-to-G editing efficiency at the region proximal to the PAM and improved simultaneous A/C conversion efficiency.

## Introduction

Base editors can directly convert one base to another in genomic DNA without double-stranded DNA cleavage^1^. They mainly include cytidine base editors (CBEs)^2^ and adenine base editors (ABEs)^3^, which can convert C•G-to-T•A and A•T-to-G•C, respectively. ABEs induces only A-to-G conversions with minimal indel rates in the genome, different from CBEs that induce by-products (e.g., C-to-G and C-to-A conversions) due to activation of base excision repair pathway^4^. In addition to single-base editors, we previously developed a dual-base editor that enables simultaneous A/C conversion by fusing adenine and cytosine deaminases with nickase Cas9^5^. About 203 pathogenic mutations have been identified contain known G-to-A and T-to-C mutations within editing windows that would be potentially corrected by dual base editors^5^. Development of dual base editors is required for advancing the research fields in disease model generation, molecular evolution, lineage tracing, genetic diversity screens and human gene therapy^5–8^.

Several important ABE variants have been developed with improved performances based on the prototype of ABE version. For example, the A-to-G base editing efficiency of ABEs can be improved through optimization of nuclear localization signals (NLS) and codon usage^9,10^. Highly active ABE8e and ABE8s are invented with the recruitment of evolved TadA monomer by engineering the TadA deaminase^11,12^. Adenine deaminase engineering has been also applied to reduce the RNA off-target effects through introducing critical amino acid mutations^13,14^. Recently, we have shown that through introduction of N108Q and L145T substitutions, ABE9 exhibits 1–2 nucleotides editing window, eliminating preferentially cytosine bystander editing and minimizing off-targeting effects on DNA and RNA^15^. However, the A-to-G base editing efficiency, especially near the PAM region, is still limited and impedes its broader applications. For dual base editors, the low activity of adenosine deaminase (TadA-TadA*) makes A/C simultaneous conversions dependent on the limited A-to-G efficiency within the A_6_-A_7_ editing window^5^. Thus, optimization strategies that have been used to improve the A-to-G efficiency can be applied to dual base editors for enhancing A/C simultaneous conversions.

In this study, we develop hyper ABE through fusion of ABE8e with Rad51 DNA-binding domain (Rad51DBD) based on previous success in optimizing CBEs^8^. hyABE exhibits higher A-to-G editing efficiency near the PAM region than that in ABE8e and comparable A-to-G editing efficiency within canonical editing window. We also introduce TadA-8e or TadA-8e/Rad51DBD into A&C-BEmax to create eA&C-BEmax or hyA&C-BEmax, respectively, which improves substantially A/C simultaneous editing efficiencies. Moreover, we show that these optimized base editors efficiently catalyze nucleotide changes in zebrafish embryos and install therapeutic mutations in the *HBG* promoter in human cells. The development of these hyper base editors expands the base editing toolbox and will advance the application of precise gene edition in basic research and clinical therapy.

## Results

### Fusion of ABE8e, not ABEmax, with Rad51DBD increases A-to-G base editing efficiency near the PAM

**Fig. 1:**
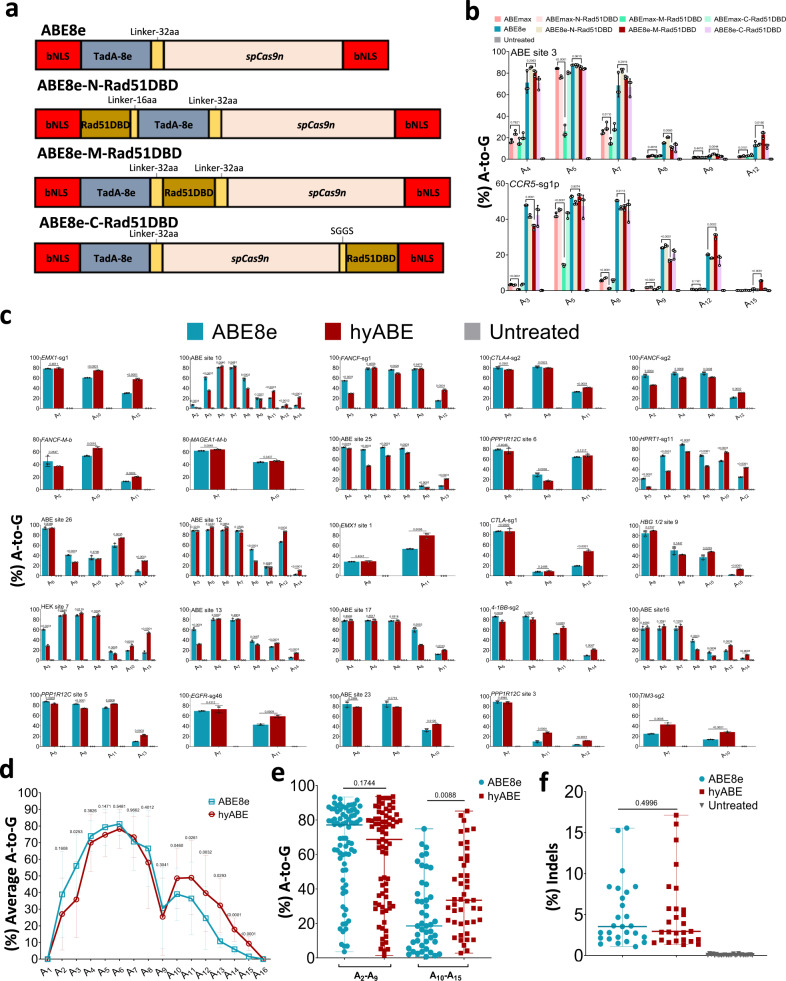
Screening and characterization of hyper ABE. **a** Schematics of the constructs with Rad51DBD fused to ABE8e. bNLS, bipartite nuclear localization signals; TadA-8e, derived from evolved *E.coil* adenosine deaminase; spCas9n, Cas9 D10A; Rad51DBD, single-strand DNA binding domain; Linkers are also shown. **b** The A-to-G base editing efficiency of ABE8e or its fusion constructs at 2 endogenous genomic loci containing multiple As (ABE site 3 and *CCR5*-sg1p) in HEK293T cells. Data are means ± SD (*n* = 3 independent experiments). **c** The A-to-G editing efficiency of ABE8e or hyABE was examined at 25 endogenous genomic loci containing multiple As in HEK293T cells. Data are means ± SD (*n* = 3 independent experiments). **d** Average A-to-G editing efficiency of ABE8e or hyABE at each position within the protospacer across the 27 target sites in **b** and **c**. **e** Summary of the A-to-G editing efficiency for A_2_-A_9_ or A_10_-A_15_ induced by ABE8e or hyABE at the 27 target sites in **b** and **c**. Each data point represents average A-to-G editing efficiency at all As within the activity window of each target site, calculated from 3 independent experiments. **f** Frequency of indels formation by ABE8e and hyABE at 27 endogenous genomic loci in **b**, **c**. Each data point represents average indels frequency at each target site calculated from 3 independent experiments. Significance was tested with two-tailed Student’s *t* test (**b**–**d**) or two-sided paired Wilcoxon rank-sum test (**e**, **f**). Source data are provided as a Source Data file.

Encouraged by previous successes in developing hyper-active cytosine base editors^8^, we sought to optimize adenine base editor through fusion of Rad51DBD. To assess the optimal fusing position, Rad51DBD was fused to the N-terminus, C-terminus of ABEmax, or between TadA-TadA* and Cas9n to generate ABEmax-N-Rad51DBD, ABEmax-C-Rad51DBD, and ABEmax-M-Rad51DBD, respectively (Supplementary Fig. 1). A-to-G conversion efficiency was tested at two endogenous sites containing multiple adenosines (ABE site 3 and *CCR5-*sg1p) in HEK293T cells (Fig. 1b). These constructs were co-transfected corresponding sgRNA into HEK293T cells. Editing outcomes were analyzed by high-throughput amplicon sequencing (HTS) analysis, and we found that none of these constructs showed improved editing efficiency (Fig. 1b). As TadA-8e showed much higher activity and compatibility^11^, we sought to fuse Rad51DBD to ABE8e (Fig. 1a). We found that the A-to-G base editing efficiency of ABE8e-N-Rad51DBD (Rad51DBD at the N-terminus of ABE8e) was increased at ABE site 3 or decreased at *CCR5*-sg1p in comparison with ABE8e (Fig. 1a, b). When Rad51DBD was fused to the C-terminus of ABE8e, the A-to-G base editing efficiencies at both ABE site 3 and *CCR5-*sg1p target sites were decreased (Fig. 1a, b). When Rad51DBD was fused between TadA-8e and Cas9n (ABE8e-M-Rad51DBD), the A-to-G editing efficiency for ABE8e-M-Rad51DBD was increased at positions near the PAM at both target sites (up to 4.4-fold improvement at A_15_ of *CCR5*-sg1p) compared to ABE8e, although the activity at positions A_3_ to A_9_ was comparable (Fig. 1a, b). Thus, we selected ABE8e-M-Rad51DBD for further investigation, and named it hyperactive ABE (hyABE) to highlight this improved editing efficiency.

To unbiasedly profile the characteristics of hyABE, additional 25 endogenous targets were tested in HEK293T cells. Through analyzing the A-to-G editing efficiency of the most efficiently edited position in each target across all 27 target sites, we found that the efficiency for hyABE was 43.0–94.6% (median 80.5%), which was similar to that of ABE8e (24.8–93.3%, median 79.7%) (Fig. 1c and Supplementary Fig. 2a). However, after further analyzing base editing efficiencies at every protospacer position, we found that the major editing window of hyABE (A_2_-A_15_) was slightly wider than that of ABE8e (A_2_-A_12_) (Fig. 1d and Supplementary Fig. 2b). Notably, base-editing efficiencies at positions near the PAM were an average of 48.6% at A_10_, 49.0% at A_11_, 37.3% at A_12_, 25.2% at A_13_, 16.3% at A_14_ and 11.7% at A_15_ for hyABE, respectively, with 1.2-, 1.3-, 1.7-, 2.9-, 3.2- and 7.0-fold improvement over that of ABE8e, suggesting that hyABE has significantly higher editing efficiency at A_10_-A_15_. (Fig. 1d, e and Supplementary Fig. 2b). Furthermore, no significant differences in A-to-G editing efficiency between hyABE and ABE8e at A_2_-A_9_ were observed except for A_3_, although the A-to-G editing efficiency of hyABE was slightly lower than that of ABE8e at some targets (A_3_ and A_8_ in ABE site 10, A_3_-A_8_ in *HPRT1*-sg11, A_3_ in *FANCF*-sg1, A_2_ in *FANCF*-sg2, A_5_ in ABE site 25 and etc.) (Fig. 1c–e). More importantly, hyABE can edit sites that ABE8e can hardly edit, such as A_14_ in ABE site 12, A_15_ in *HBG 1/2* site 9, A_14_ in ABE site 16 and A_12_ in *PPP1R12C* site 3 (Fig. 1c). Finally, we assessed indel formation caused by hyABE at 27 endogenous genomic loci and found that hyABE retained a very low indel rate compared to ABE8e (Fig. 1f). These findings indicate that the optimization for ABE8e, not ABEamx, elevates its base editing efficiency at positions near the PAM.

### Substantially improving dual base editors through introduction of TadA-8e and Rad51DBD

**Fig. 2:**
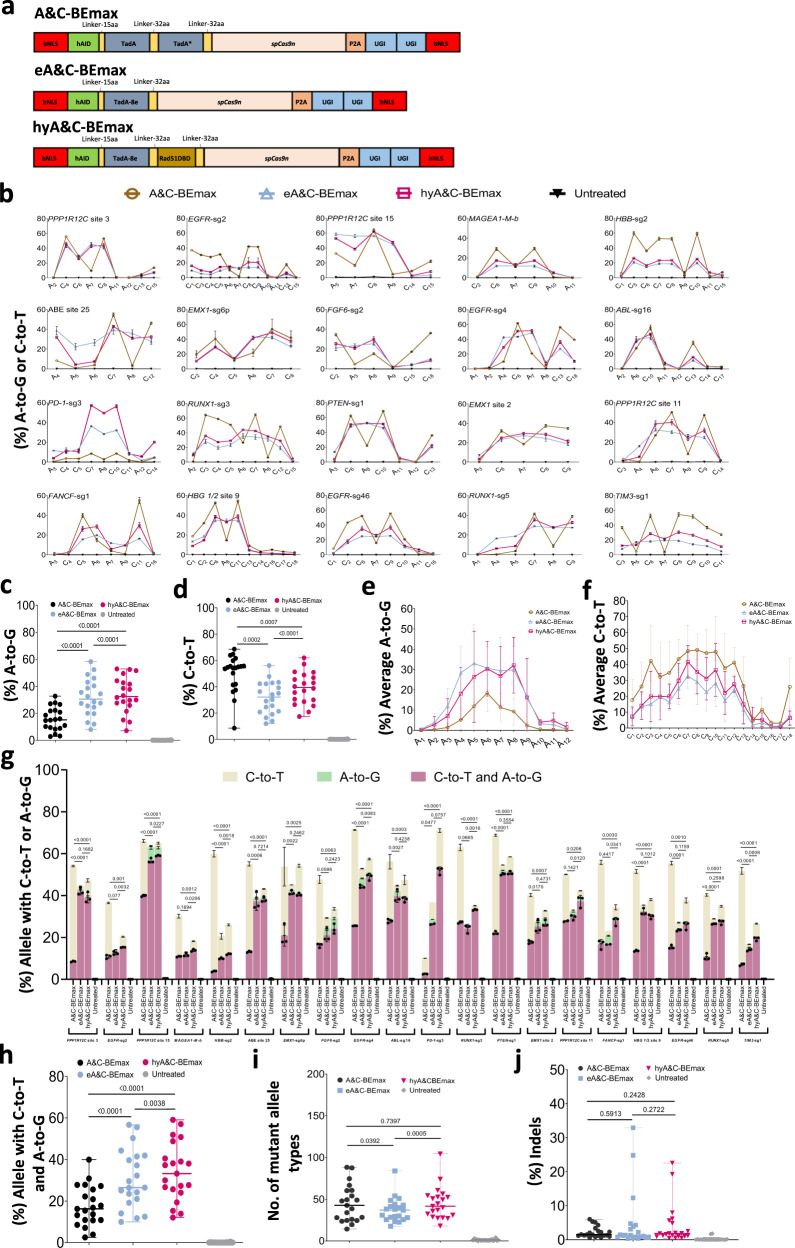
Screening and characterization of hyper A&C-BEmax. **a** Schematics of the constructs with Rad51DBD fused to A&C-BEs. **b** The A-to-G or C-to-T editing efficiency of A&C-BEmax, eA&C-BEmax or hyA&C-BEmax was examined at 20 endogenous target sites in HEK293T cells. Data are means ± SD (*n* = 3 independent experiments). **c** Summary of the A-to-G editing efficiencies for only the most highly edited adenine induced by dual base editor at the 21 target sites in **b** and Supplementary Fig. 3b. **d** Summary of the C-to-T editing efficiencies for only the most highly edited cytosine induced by dual base editors at the 21 target sites in **b** and Supplementary Fig. 3b. **e** Average A-to-G editing efficiency of A&C-BEmax, eA&C-BEmax or hyA&C-BEmax at the same 21 target sites in **b** and Supplementary Fig. 3b. Data are means ± SD (*n* = 3 independent experiments). **f** Average C-to-T editing efficiency of A&C-BEmax, eA&C-BEmax, or hyA&C-BEmax at the same 21 target sites in **b** and Supplementary Fig. 3b. Data are means ± SD (*n* = 3 independent experiments). **g** The allele with A-to-G or C-to-T efficiency of A&C-BEs at 21 endogenous target sites in HEK293T cells. Data are means ± SD (*n* = 3 independent experiments). **h** Frequency of A/C simultaneous conversion induced by A&C-BEmax, eA&C-BEmax, and hyA&C-BEmax at 21 endogenous genomic loci in **b** and Supplementary Fig. 3b. **i** Number of mutant allele types formation by A&C-BEmax, eA&C-BEmax or hyA&C-BEmax at 21 endogenous genomic loci in **b** and Supplementary Fig. 3b. **j** Frequency of indels formation by A&C-BEmax, eA&C-BEmax or hyA&C-BEmax at 21 endogenous genomic loci in **b** and Supplementary Fig. 3b. For **c**, **d**, Each data point represents means at indicated target sites from 3 independent experiments. For **h**–**j**, Each data point represents means at indicated target sites calculated from 3 independent experiments. Significance was tested with two-tailed Student’s *t* test (**e**–**g**) or two-sided paired Wilcoxon rank-sim test (**c**, **d** and **h**–**j**). Source data are provided as a Source Data file.

**Fig. 3:**
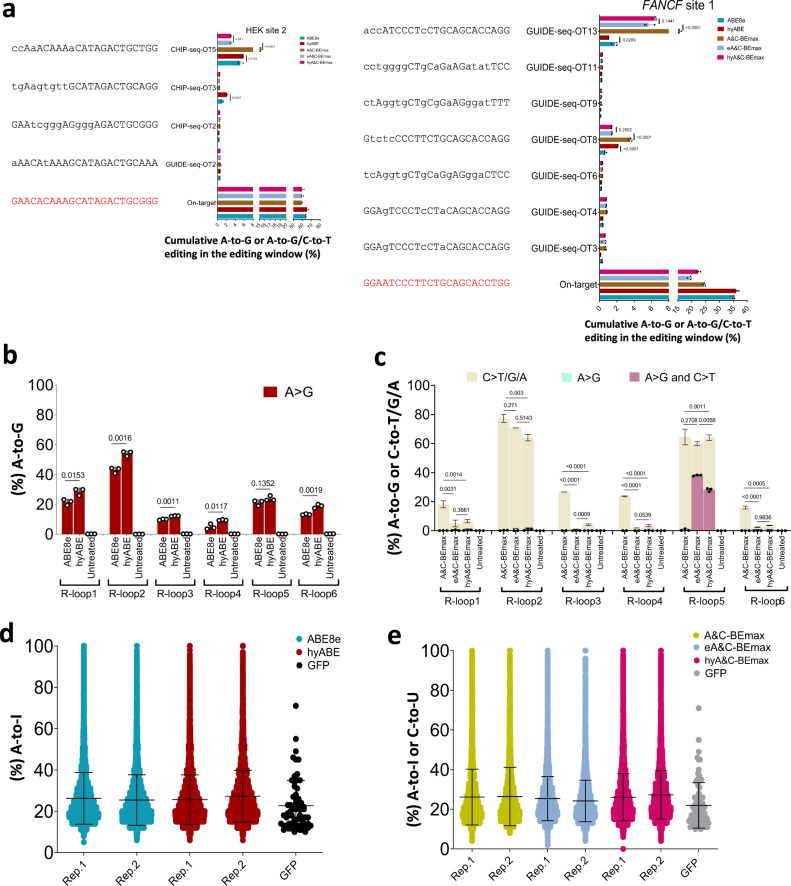
Off-target assessments of hyper ABE and hyper A&C-BEmax. **a** Cas9-dependent DNA on and off-target analysis of the indicated targets (HEK site 2 and *FANCF* site 1) by ABE8e, hyABE, A&C-BEmax, eA&C-BEmax and hyA&C-BEmax in HEK293T cells. Lowercase protospacer sequences represent mismatched bases compared to their corresponding on-target sequences. Data are means ± SD (*n* = 3 independent experiments). **b**, **c** Cas9-independent DNA off-target analysis of ABE8e, hyABE, A&C-BEmax, eA&C-BEmax, and hyA&C-BEmax using the modified orthogonal R-loop assay at each R-loop site with nSaCas9-sgRNA. Data are means ± SD (*n* = 3 independent experiments). **d**, **e** RNA off-target editing activity by ABE8e, hyABE, A&C-BEmax, eA&C-BEmax and hyA&C-BEmax using RNA-seq. Each biological replicate is listed on the bottom. For **a**–**c**, significance was tested by two-tailed Student’s *t* test. Source data are provided as a Source Data file.

We recently developed dual base editor (A&C-BEmax) through fusion of adenine deaminase and cytosine deaminase with Cas9n^5^. However, the low simultaneous A/C base conversion efficiency restrains its wide applications. Since the simultaneous A/C editing efficiency for dual-base editor depends on limited A-to-G efficiency within the A_6_-A_7_ editing window, we sought to optimize A&C-BEmax by improving the A-to-G base editing efficiency. Rad51DBD was fused between AID-TadA-TadA* and Cas9n to generate A&C-BEmax-M-Rad51DBD (Fig. 2a and Supplementary Fig. 3a). After co-transfecting A&C-BEmax-M-Rad51DBD with *CTLA4-*sg2 into HEK293T cells following HTS, C-to-T base editing efficiency of A&C-BEmax-M-Rad51DBD was comparable; however, A-to-G base editing efficiency was decreased in comparison to A&C-BEmax (Supplementary Fig. 3b). Simultaneous A/C conversion efficiency of A&C-BEmax-M-Rad51DBD in same allele was also reduced compared to A&C-BEmax (Supplementary Fig. 3c). One possible reason is the poor compatibility of TadA-TadA* in A&C-BEmax. By replacing TadA-TadA* with TadA-8e in A&C-BEmax, the A-to-G editing efficiency is greatly improved compared with A&C-BEmax, while the C-to-T efficiency near the PAM remained comparable, and C-to-T efficiency in the distal of the PAM was slightly decreased (Fig. 2a and Supplemantary Fig. 3b). Simultaneous A/C conversion efficiency for the enhanced A&C-BEmax (eA&C-BEmax) is 1.8-fold higher than that of A&C-BEmax at *CTLA4*-sg2 site (Supplementary Fig. 3c). To further improve the performance of eA&C-BEmax, we also introduced Rad51DBD into eA&C-BEmax and tested it in HEK293T cells (Fig. 2a). Our results showed that the editing efficiency of A-to-G or C-to-T was similar to that of eA&C-BEmax and simultaneous A/C conversion efficiency for eA&C-BEmax-M-Rad51DBD was 1.9-fold higher than for A&C-BEmax at *CTLA4*-sg2 (Supplementary Fig. 3b, c). Thus, we named eA&C-BEmax-M-Rad51DBD as hyperactive A&C-BEmax (hyA&C-BEmax).

Next, we tested another 20 endogenous targets containing As and Cs in HEK293T cells to unbiasedly profile the characteristics of eA&C-BEmax and hyA&C-BEmax (Fig. 2b). By analyzing the most efficiently edited position of A-to-G or C-to-T across all 21 target sites, we found that the A-to-G editing efficiency for these optimized base editors was substantially increased (Fig. 2b, c and Supplementary Fig. 4). The A-to-G efficiencies for hyA&C-BEmax (median 32.4%) were higher than that of eA&C-BEmax (median 30.5%), both of which were higher than A&C-BEmax (median 15.1%) (Fig. 2c). However, the C-to-T editing activity for these optimizeddual base editors was lower than A&C-BEmax (median 55.5%) (Fig. 2d). Despite that, the C-to-T efficiencies for hyA&C-BEmax (median 39.5%) were higher than eA&C-BEmax (median 32.2%) (Fig. 2d). Through analyzing base editing induced by these dual base editors at every protospacer position, we found that both eA&C-BEmax and hyA&C-BEmax have a similar major A-to-G editing window (A_3_-A_9_), which was wider than A&C-BEmax (A_4_-A_8_) (Fig. 2e and Supplementary Fig. 5a). The average A-to-G efficiencies for eA&C-BEmax and hyA&C-BEmax across the protospacers were substantially improved, with average 1.1~10.1-fold and 1.2~17.7-fold higher than A&C-BEmax, respectively (Fig. 2e and Supplementary Fig. 6a). Moreover, the average A-to-G efficiency for eA&C-BEmax was slightly higher than hyA&C-BEmax at positions A_3_-A_7._ Interestingly, the results were just the opposite at positions A_8_-A_12_ (Fig. 2e and Supplementary Fig. 6a). We also found that A&C-BEmax, eA&C-BEmax and hyA&C-BEmax have a similar major C-to-T editing window (C_1_-C_15_) (Fig. 2f and Supplementary Fig. 5b). However, the average C-to-T efficiencies across almost all the protospacers for eA&C-BEmax and hyA&C-BEmax were reduced compared to A&C-BEmax, in which hyA&C-BEmax exhibited the slightly higher C-to-T average efficiency than eA&C-BEmax at positions C_6_-C_10_ (Fig. 2f and Supplementary Fig. 6b).

By further analyzing simultaneous A/C conversion efficiency, we found that at most of the 21 tested target sites, the simultaneous A/C conversion efficiency was greatly improved, exhibiting 10.0–55.5% (median 26.4%) and 12.1–57.0% (median 33.2%) for eA&C-BEmax and hyA&C-BEmax, respectively, compared with 2.5–30.9% (median 22.2%) for A&C-BEmax (Fig. 2g, h). At 9 of 21 tested target sites, hyA&C-BEmax exhibited higher efficiency than eA&C-BEmax, and for the remaining 12 targets, hyA&C-BEmax had similar or slightly low efficiencies (Fig. 2g and Supplementary Fig. 3c). Next, we analyzed the correlation of sequence and simultaneous A/C conversion. hyA&C-BEmax tended to have the higher A/C simultaneous conversion efficiency than eA&C-BEmax, when editable As were at position A_8_-A_12_ and editable Cs were at position C_6_-C_10_ concurrently (Fig. 2b, g, h and Supplementary Fig. 6c). In addition, hyA&C-BEmax generated similar mutant allele types to A&C-BEmax but mutant allele types for eA&C-BEmax are lower than hyA&C-BEmax and A&C-BEmax (Fig. 2i). hyA&C-BEmax and eA&C-BEmax did not generate more indels than A&C-BEmax (Fig. 2j). We also further compared the editing efficiency of hyA&C-BEmax with the mix of hyABE and hyAID-BE4max at 15 targets in HEK293T cells. The results showed that, at 8 of 15 targets, hyA&C-BEmax exhibited the higher A/C simultaneous editing efficiency than the mix of hyABE and hyAID-BE4max. At the remaining 7 targets, hyA&C-BEmax had similar or lower editing efficiencies for simultaneous A/C conversion than the mix of hyABE and hyAID-BE4max (Supplementary Fig. 7a, b). Overall, hyA&C-BEmax induced the higher simultaneous A/C editing efficiency than the mix of hyABE and hyAID-BE4max (Supplementary Fig. 7c).

It has been shown that ABEs have cytosine deaminase activity in the confined TC*N sequence context, suggesting that ABE enables simultaneous A/C conversion^16^. Our findings also indicate that ABE8e induces simultaneous A/C conversion across four targets containing a TC*N motif, but with an editing efficiency much lower than hyA&C-BEmax (Supplementary Fig. 8a, b).

### Off-target evaluation for hyper base editors

To assess the Cas9-dependent potential DNA off-target activity of hyABE, eA&C-BEmax and hyA&C-BEmax, 28 off-target sites in total – 19 of which were from 3 previously known Cas9 off-target sites (HEK site 2, HEK site 3 and *FANCF* site 1) identified by GUIDE-seq or ChIP-seq^2^ and 9 were predicted off-target sites from *CCR5-*sg1p using Cas-OFFinder program^17^ were examined (Fig. 3a and Supplementary Fig. 9). The results showed that hyABE and ABE8e displayed very low editing efficiencies at 5 of 28 these off-target sites (HEK site 2-ChIP-seq-OT3, HEK site 2-ChIP-seq-OT5, HEK site 3-GUIDE-seq-OT3, *FANCF* site 1-GUIDE-seq-OT8 and -OT13), and at 3 of 5 off-target sites, hyABE exhibited no higher efficiency than ABE8e (Fig. 3a and Supplementary Fig. 9). Similarly, A&C-BEmax induced very low editing efficiencies at 5 of 28 off-target sites. And 3 of 5, these off-target sites (HEK site 2-ChIPseq-OT5, *FANCF* site 1-GUIDE-seq-OT8 and -OT13), eA&C-BEmax and hyA&C-BEmax showed very low efficiency and at the remaining 2 off-target sites (HEK site 3-GUIDE-seq-OT5 and CHIP-seq-OT4), and no off-target editing for eA&C-BEmax and hyA&C-BEmax was observed (Fig. 3a and Supplementary Fig. 9).

We next evaluated the Cas9-independent DNA off-target effects for hyper BEs using a modified orthogonal R-loop assay^18^. The data showed that hyABE induced significantly higher Cas9-independent A-to-G DNA off-target efficiencies than ABE8e at 5 of the 6 tested R-loop sites (Fig. 3b and Supplementary Fig. 10a). Interestingly, both eA&C-BEmax and hyA&C-BEmax induced lower Cas9-independent DNA off-target editing than A&C-BEmax at almost all of the 6 tested R-loop sites. At 4 of 6 tested R-loop sites, the Cas9-independent DNA off-target editing events by these two dual base editors were close to background (Fig. 3c and Supplementary Fig. 10b). This is likely due to the reduced cytosine deaminase activity of eA&C-BEmax and hyA&C-BEmax (Fig. 3c and Supplementary Fig. 10b). Since these dual base editors primarily induced Cas9-independent C-to-T/G/A DNA off-targets, suggesting that the Cas9-independent DNA off-target editing was mainly attributed to cytosine deaminase (Fig. 3c and Supplementary Fig. 10b).

Since adenine base editors have also been reported to generate numerous unpredictable A-to-I off-target RNA editing events^13,14^, we further evaluated RNA off-target effects using RNA-Seq after sorting the top 15% GFP positive HEK293T cells treated by these optimized base editors. The results showed that the average RNA off-target editing efficiency between hyABE and ABE8e was similar, though hyABE induced slightly more RNA off-target events than ABE8e (Fig. 3d). Similarly, A&C-BEmax, eA&C-BEmax, and hyA&C-BEmax exhibited comparable off-target editing efficiencies and there were slightly more RNA off-target events for eA&C-BEmax and hyA&C-BEmax than for A&C-BEmax were observed (Fig. 3e). Despite some Cas9-independent DNA off-target mutations, hyABE, eA&C-BEmax and hyA&C-BEmax show great potential, especially as these issues could be rapidly solved with the engineering of adenosine and cytidine deaminases. Collectively, the reported findings demonstrate that hyABE, eA&C-BEmax, and hyA&C-BEmax are efficient programmable tools for targeted base editing.

### Efficiently install therapeutic mutations and generate zebrafish mutants with hyper BEs

**Fig. 4:**
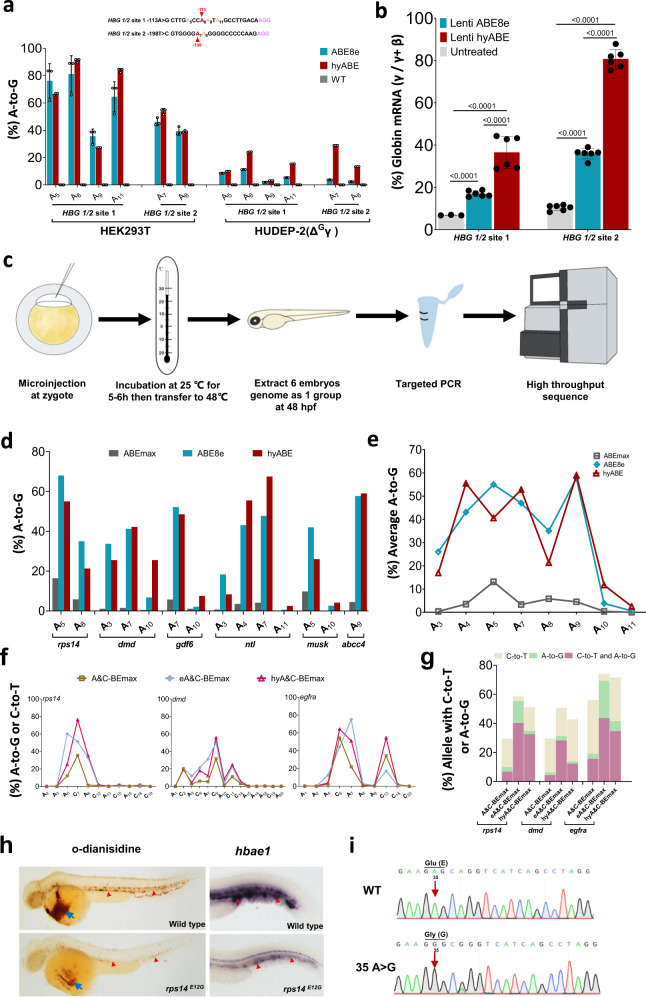
Efficiently install therapeutic mutations and generate zebrafish mutants with hyper BEs. **a** The comparison of base editing efficiency by ABE8e and hyABE at *HBG 1/2* −113 A-to-G or −198 T-to-C in HEK293T cells and HUDEP-2 (Δ^G^γ) cells. Data are means ± SD (*n* = 3 independent experiments). **b** Comparison of γ-globin mRNA expression relative to β-like globin mRNA via hyABE treatment in HUDEP-2 (Δ^G^γ) cells after differentiation. Data are means ± SD (*n* = 3 for one untreated group and *n* = 6 for the other untreated or treated group). *P* value was determined by two-tailed Student’s *t*-test. **c** The flow chart for generating zebrafish mutants with hyABE and hyA&C-BEmax. **d** The A-to-G base editing efficiency of hyABE, ABE8e or ABEmax at endogenous target sites for *ntl*, *dmd*, *musk*, *rps14*, *gdf6* and *abcc4* in zebrafish embryos. **e** The average A-to-G editing efficiency of hyABE, ABE8e, or ABEmax across protospacers at the endogenous targets shown in **b**. **f** The A-to-G or C-to-T editing efficiency of A&C-BEmax, eA&C-BEmax or hyA&C-BEmax was examined at 3 endogenous target sites in zebrafish embryo. **g** The composition of A&C-BEmax, eA&C-BEmax and hyA&C-BEmax base editing products at 3 endogenous zebrafish genomic loci. The individual data are shown as yellow (only C-to-T), green (only A-to-G) and plum purple (simultaneous C-to-T and A-to-G) columns. **h** The hematopoietic phenotypes of *rps14*^E12G^ embryos. O-dianisidine staining exhibits a reduction of erythrocytes at the yolk sac (blue arrow) and the trunk region (red triangle) in *rps14*^E12G^ embryos compared to wild-type (WT) control embryos at 48 hpf (left). WISH analysis for *hbae1* expression (red triangle) in *rps14*^E12G^ mutant embryos and WT control embryos at 72 hpf (right). **i** Sanger sequencing chromatograms of DNA from a WT embryo and a single F0 *rps14*^E12G^ embryo carrying the A to G conversion at the nucleotide 35 (arrow) that causes Glu (E) to Gly (G) substitution (Red arrow). Source data are provided as a Source Data file.

To investigate the potential of the developed BEs for gene therapy, we focused on well-known β-hemoglobinopathy, which can be treated by reactivation of fetal hemoglobin (HbF)^19^. Natural mutations, such as −113 A > G, −198 T > C and −114 or −115 C > T in the promoter of *HBG1/HBG2*, can reactivate the expression of γ-globin, causing high-level HbF expression^19^. −113 A > G, −198 T > C and −114 or −115 C > T in the promoter of *HBG 1/2* reactivate the expression of γ-globin through generating de novo binding sites for the activator *GATA1*^20^ and *KLF1/EKLF*^21^, as well as disrupting the binding motif of repressors *BCL11A*^22^, respectively. Natural mutations could alleviate anemic symptoms in β-thalassemia patients^19^. To test the ability of these optimized base editors to introduce these therapeutic mutations, we attempted to design sgRNAs targeting −113 (A_8_) or the complementary strand base (A_7_) of −198 in the promoter of the *HBG 1/2* gene using hyABE. After co-transfecting hyABE and *HBG 1/2* site 1 or *HBG 1/2* site 2 sgRNA in HEK293T cells, we found that hyABE induced higher base editing efficiencies than ABE8e at the two target sites (91.0% vs. 81.2 % at −113, 53.9% vs. 46.1% at −198), though some bystander editing events between hyABE and ABE8e were also observed (Fig. 4a). We previously reported that A&C-BEmax could induce high *HBG* reactivation through combining −113 A > G with −114 or −115 C > T mutation in human cells^5^. Thus, we targeted −113 A > G and −114 or −115 C > T in the promoter of *HBG1/HBG2* using our optimized dual base editors. Our observations showed both eA&C-BEmax and hyA&C-BEmax induced much higher simultaneous conversion for −113 A > G and −114 or −115 C > T (33.8% and 29.5%) than A&C-BEmax (13.5%), and similar editing efficiency between eA&C-BEmax and hyA&C-BEmax were also observed (Supplementary Fig. 11–12). We next employed an erythroid precursor cell line HUDEP-2 (Δ^G^γ) to evaluate the editing efficiency and biology function of *HBG 1/2* by the hyper BEs. Lentivirus-packaged hyABE containing *HBG 1/2* site 1 or *HBG 1/2* site 2 sgRNA were delivered to the HUDEP-2 (Δ^G^γ) cells (Supplementary Fig. 13). We observed that hyABE induced higher editing efficiency than ABE8e at desired base pairs, with 24.1% vs. 11.3% −113 A-to-G and 29.0% vs. 3.7% −198 T-to-C conversion, respectively (Fig. 4a). After 7 days HUDEP-2 (Δ^G^γ) cell differentiation, higher γ-globin mRNA levels were observed in both *HBG 1/2* site 1 and *HBG 1/2* site 2 edited by hyABE compared to ABE8e (Fig. 4b). These results suggest that these developed highly active base editors may provide an efficient and alternative platform for gene therapy in thalassemia.

To evaluate whether the hyper BEs induce efficiently base editing in zebrafish embryos, hyABE, ABE8e or ABEmax was co-microinjected with each of previously tested sgRNAs (*ntl*, *dmd*, *gdf6*, *musk, abcc4* or *rps14*)^23,24^ into zebrafish embryos at the one-cell stage. We collected pools of 6 injected embryos that developed at 48 h post fertilization (hpf) and extracted genomic DNA to conduct HTS (Fig. 4c). We showed that both hyABE and ABE8e exhibited highly efficient editing within the canonical editing window (A_3_-A_9_) over different target sites compared to ABEmax that had been previously tested in zebrafish^23^ (Fig. 4d, e). The editing efficiency (average 16.9 −59.0%) for hyABE at A_3_-A_9_ was comparable to ABE8e (average 26–57.7%) (Fig. 4d, e). Moreover, the A-to-G conversion efficiency in hyABE (2.5–11.7%) was higher at the positions near the PAM (A_10_-A_11_) than that in ABE8e (0.6–3.8%) (Fig. 4d, e), consistent with the results in HEK293T cells. We also tested the toxicity of ABE mRNAs and found overexpression of hyABE or ABE8e caused growth defects or morphological changes at high dosages (400 ng μl^−1^) but not at normal dosages (100 ng μl^−1^−200 ng μl^−1^) used for our base editor injections (Supplementary Fig. 14 and Supplementary Table 1). Next, we assessed the editing capacity of dual base editors eA&C-BEmax, hyA&C-BEmax and A&C-BEmax at 5 endogenous targets (*rps14, dmd, egfra, gdf6* and *pspc1*). We found that the As or Cs within editing window at *rps14, dmd* or *egfra* were efficiently converted by eA&C-BEmax and hyA&C-BEmax (Fig. 4f, g and Supplementary Fig. 15). However, the As at the other 2 targets (*gdf6* and *pspc1*) were edited at low efficiencies by hyA&C-BEmax or A&C-BEmax compared to eA&C-BEmax, while the Cs at these two loci were comparably converted among the dual base editors (Supplementary Fig. 15, 16a), suggesting the sequence preference for different dual base editors in editing As or Cs. Overall, both eA&C-BEmax and hyA&C-BEmax exhibited the higher A/C simultaneous conversion than A&C-BEmax at these targets (Fig. 4g Supplementary Fig. 16b). Taken together, these findings demonstrate that hyper BEs, including dual base editors, can induce base pair conversions in zebrafish embryos.

It has been shown that the 35 A > G mutation in the gene for *ribosomal protein S14* (*rps14*), a candidate gene for 5q-syndrome, a distinct form of mylodysplastic syndrome (MDS), causes erythroid failure and anemia in zebrafish, similar to what is observed in human patients^25^. To evaluate the effects of hyper BEs in generating anemic disease models in zebrafish, we microinjected zebrafish embryos at the one-cell stage with hyABE mRNA and 35 A > G sgRNA. About 90% of the injected embryos survived at 48 hpf (Supplementary Table 2). Notably, 41.9% injected F0 embryos displayed a marked reduction of erythrocytes using o-dianisidine staining (Supplementary Fig. 17a, b), whereas 32.3% F0 embryos exhibited a moderate o-dianisidine reduction and 25.8% F0 embryos showed wild-type like phenotypes (Supplementary Fig. 17a, b). Whole mount in situ hybridization (WISH) further indicated a severe reduction in *hemoglobin alpha embryonic 1* (*hbae1*) in 40.0% of injected F0 embryos at 72 hpf, while 36.7% F0 embryos exhibited a moderate *hbae1* reduction (Supplementary Fig. 17a, b). These findings reveal markedly high efficiency for A-G conversion with hyABE in zebrafish F0 embryos. By contrast, application of the zebrafish codon-optimized ABE7.10 (zABE7.10) induced the anemic MDS phenotype of *rps14*^*E12G*^ only in F1 germline transmission embryos^23^. The A-G conversion that induced E12G missense mutation in single F0 embryos was validated by Sanger sequence analysis (Fig. 4h, i). We found that 13.9% of injected F0 embryos showed 35 A > G complete mutation, while 59.5% and 26.6% F0 embryos displayed high mosaic and low mosaic 35 A > G mutation, respectively (Supplementary Table. 3). Conversely, embryos injected with ABEmax/*rps14* sgRNA failed to show 35 A > G complete mutation or high mosaic mutation (Supplementary Table 3). Overall, the degree of *rps14* mutagenesis by hyABE correlates with the severity of reduced erythrocytes and *hbae1* expression (Supplementary Fig. 17c, d). These findings illustrate that hyABE is a powerful base editing tool for generating animal disease models.

## Discussion

In this study, we have developed three base editors: hyABE, eA&C-BEmax, and hyA&C-BEmax. hyABE exhibits a higher activity at positions (A_10_–A_15_) near the PAM than ABE8e, but has the comparable editing efficiency at positions (A_2_–A_9_) relative to ABE8e. This is slightly different from the characteristics of hyCBEs which have higher base editing efficiencies across all protospacers. One possible reason is the inherent properties of the TadA-8e enzyme, which may possess high single-stranded DNA binding capacity. In addition, ABE8e displays much higher efficiency within its editing window than ABEmax^11^, which has a similar editing activity towards the PAM as CP-ABEmax^26^. We can fully speculate that hyABE should have higher activity than CP-ABEmax across all protospacers. Although hyABE is desirable for the proximal of the PAM region, it would bring more bystander editing. Future base editor engineering might alleviate its defects in bystander editing activity.

We find that both eA&C-BEmax and hyA&C-BEmax substantially elevate simultaneous A/C conversion efficiency, but the efficiency improvement of hyA&C-BEmax relative to eA&C-BEmax is much lower than that of eA&C-BEmax relative to A&C-BEmax, suggesting that the introduction of TadA-8e with high compatibility plays a key role in efficiency improvement. Moreover, the average A-to-G efficiencies for eA&C-BEmax and hyA&C-BEmax across the protospacers were increased, but the C-to-T conversions for eA&C-BEmax and hyA&C-BEmax were reduced compared to A&C-BEmax. This is likely due to the difference in the ability of cytosine deaminase and adenosine deaminase to competitively bind single-stranded DNA, in which the binding capacity of AID to single-stranded DNA might be much higher than that of TadA-TadA* but weaker than that of TadA-8e. Fusing Rad51DBD to eA&C-BEmax further improves simultaneous A/C conversion efficiency, overcoming the limitation on dependence of the positions of narrow editing window_._ Through this mechanism, other similar dual-base editors with poor activity, such as SPACE^6^, Target-ACEmax^7^ and STEMEs^8^, can also be modified into the corresponding highly efficient version.

Off-target evaluation showed hyABE had some off-target events, including Cas9-independent DNA off-target and RNA off-target events (Fig. 3), which are mainly caused by TadA-8e. Future experiments for TadA-8e engineering and mutation introduction, such as V106W, are required to alleviate the Cas9-independent DNA/RNA off-target editing for hyABE^11^. hyA&C-BEmax and eA&C-BEmax induced Cas9-independent C-to-T/G/A DNA off-target and A-to-I RNA off-target, which might be due to AID and TadA-8e, respectively. Thus, both TadA-8e and AID engineering and introduction of mutations that reduce C-to-T/G/A DNA off-target in AID-BEs or decrease A-to-I RNA off-target in TadA-8e would be able to alleviate the off-target problems for both hyA&C-BEmax and eA&C-BEmax.

Our study has demonstrated that hyper adenine and dual base editors induce effectively nucleotide conversions in zebrafish embryos. We observed that hyper adenine or dual base editors catalyzed nucleotide substitutions at different target sites with various efficiencies, suggesting the sequence preference by these base editors in different genomic loci. In comparison to human HEK293T cells, the editing efficiencies of hyper BEs are still relatively low in zebrafish embryos. This might be due to species-specific issues associated with nucleotide editing tools. Zebrafish embryos undergo rapid cell division with diverse cell differentiation during development, which may somehow decrease base editing efficiencies. Nevertheless, the anemic phenotype in the *rps14*^*E12G*^ mutation introduced by hyABE can be observed even in zebrafish F0 embryos, reflecting impressively high efficiencies for generating disease models. Given that zebrafish codon optimization of base editors improves A-G conversion^23^, future experiments are required to further improve the editing efficiency of hyper BEs in zebrafish embryos.

In summary, we have developed two hyper active base editors: hyABE with increased A-to-G efficiency near the PAM and eA&C-BEmax/hyA&C-BEmax with substantially improved A/C simultaneous conversion efficiency. These hyper BEs provide great promise and future application for disease model generation and potential gene therapy.

## Methods

### Ethical statement

Our research complies with all relevant ethical regulations, and all Zebrafish experiments conformed to the regulations and approved by Institutional Animal Care and Use Committees (IACUCs) of East China Normal University.

#### Plasmid construction

The primers and DNA sequences used in this research can be both found in Supplementary Data 1–3 and in Supplementary Note 3, 4. Rad51DBD (amino acids 1–114) was amplified from hyBE4max, which we donated to Addgene (#157942). ABEmax (#112095) and ABE8e (#138489) were purchased from Addgene. A&C-BEmax (#157947) or Lenti ABE7.10-N-AIDmax (#157949) comes from our lab. PCR was performed using PrimeSTAR® Max DNA Polymerase (TaKaRa, code no. R045A) or KOD-Plus-Neo DNA Polymerase (Toyobo, code no. KOD-401). Serial ABEs or A&C-BEs plasmids generated in this article were constructed using ClonExpress MultiS One Step Cloning Kit (Vazyme). sgRNA expression plasmids were constructed as described previously^5^. Briefly, oligonucleotides listed in Supplementary Data 1 were denatured at 95 °C for 5 min followed by slow cooling to room temperature. Annealed oligonucleotides were ligated into BbsI-linearized U6-sgRNA(sp)-EF1α-GFP for sgRNA expression (Thermo Fisher Scientific).

#### Human cell culture

HEK293T (ATCC CRL-3216) cell lines were cultivated in Dulbecco’s Modified Eagle’s medium (DMEM, Gibco) with 10% (vol/vol) fetal bovine serum (FBS, Gibco) and 1% Penicillin-Streptomycin (Gibco) antibiotic mix. HUDEP-2 cells were maintained and expanded in serum-free expansion medium (Stem Cell Technologies) supplemented with human Stem Cell Factor (SCF, 50 ng ml^−1^, PeproTech), erythropoietin (EPO, 3 IU ml^−1^, PeproTech), dexamethasone (1 µM, Sigma), doxycycline (1 µg ml^−1^, Takara Bio) and 2% penicillin–streptomycin (Gibco). All cell lines used were maintained at 37 °C, 5% CO_2_ in the incubator.

#### Cell transfection and genomic DNA extraction

For DNA base editing experiments, HEK293T cells were seeded into 24-well plates and transfected at approximately 80% confluency. Next, a mixture of 3 μl polyethyleneimine (PEI, Polysciences), 1 μg plasmid DNA (750 ng ABEs or A&C-BEs expression plasmid and 250 ng sgRNA expression plasmid) and serum-free medium were added to the cells. After 3 days, transfected cells were digested with 0.25% trypsin (Gibco) and then genomic DNA was isolated using the QuickExtract™ DNA Extraction Solution (QE09050, Epicenter) according to the manufacturer’s instructions. For RNA off-target analysis, HEK293T cells were seeded into 10-cm dishes and transfected with 30 μg of Cas9n-P2A-GFP, ABE8e-P2A-GFP, hyABE-P2A-GFP, A&C-BEmax, eA&C-BEmax and hyA&C-BEmax using PEI at approximately 80% confluency. After 3 days, transfected cells were washed with phosphate-buffered saline and digested with 0.25% trypsin (Gibco) for fluorescence-activated cell sorting (FACS). FACS was carried out on a FACSAriaIII (BD Biosciences) using FACSDiva version 8.0.2 (BD Biosciences). Cells were gated on their population via forward/sideward scatter after doublet exclusion (Supplementary Note 1, 2). Then, cells (300,000 to 400,000 cells) with top 15% GFP signal were collected, and total mRNA was extracted using RNAiso Plus (Takara).

#### Enhanced orthogonal R-loop assay

An enhanced orthogonal R-loop assay was used in this study for Cas9-independent DNA off-target analysis, which replaced dSaCas9-sgRNA plasmid with nSaCas9-sgRNA plasmid at each R-loop site. For transfection, a mixture of 3 μl polyethyleneimine (PEI, Polysciences), 850 ng plasmid DNA (250 ng SpCas9 sgRNA plasmid, 300 ng base editor plasmid (ABE8e, hyABE, A&C-BEmax, eA&C-BEmax or hyA&C-BEmax) and 300 ng nSaCas9 containing sgRNA plasmid were added to the cells. After 3 days, transfected cells were digested with 0.25% trypsin (Gibco) for sorting and then genomic DNA was isolated using the QuickExtract™ DNA Extraction Solution (QE09050, Epicenter) according to the manufacturer’s instructions.

### RNA sequencing (RNA-Seq) experiments

RNA sequencing experiments were performed as previously described^5,8^. In brief, 3 μg RNA per sample was used for library construction. Sequencing libraries were constructed using a NEBNext Ultra RNA Library Prep Kit for Illumina (NEB) according to manufacturer’s instructions. TruSeq PE Cluster Kit v3-cBot-HS (Illumina) were used to barcode each sample with unique dual index after libraries quality assess using Agilent Bioanalyzer 2100 system. Then, the RNA-seq libraries were sequenced on an Illumina HiSeq platform and 125 bp/150 bp paired-end reads were obtained.

### RNA sequence variant calling and quality control

RNA sequence variant calling and quality control were performed as previously described^5,8^. In brief, raw data of FASTQ format were first processed using in-house Perl scripts. First, clean data were obtained by removing reads containing adapters and low-quality bases were trimmed with Trimmomatic. The Q20, Q30, and GC contents of the clean data were also evaluated. An index of the reference genome was built using HISAT2 version 2.0.5 and paired-end clean reads were aligned to the reference genome (Ensemble GRCh38) using HISAT2 version 2.0.5. Single-nucleotide polymorphism calling were performed using GATK (version 4.0) software. Variant loci in base editor overexpression groups were filtered to exclude sites without high-confidence reference genotype calls in the control group. The read coverage for a given single-nucleotide variant in a control group should be greater than the 90th percentile of the read coverage across all single-nucleotide variants in the corresponding overexpression group. These loci were also required to have a consensus of at least 99% of reads containing the reference allele in the control groups. RNA editing events in GFP controls were filtered to include only loci with ten or more reads and with greater than 0% of reads containing alternate alleles. Base edits labeled as “A-to-I” or C-to-U if they were A-to-I edits called on the positive strand and T-to-C edits from the negative strand or they were C-to-U edits called on the positive strand and G-to-A edits from the negative strand.

#### HUDEP-2 cell differentiation

HUDEP-2 cell differentiation were performed as previously described^5,8^. Erythroid differentiation media (IMDM supplemented with 2% human blood type AB plasma (SeraCare), 1% L-glutamine, 2 IU ml^−1^ heparin, 10 µg ml^−1^ recombinant human insulin, 3 IU ml^−1^ EPO, 330 μg ml^−1^ holo-human transferrin (Sigma-Aldrich), 100 ng ml^−1^ SCF, 1 μg ml^−1^ doxycycline and 2% penicillin–Streptomycin) were used to differentiate HUDEP-2 cells. After 8 days differentiation, cells were harvested for total mRNA preparation.

### mRNA preparation and quantitative PCR for HUDEP-2 cells

RNAiso Plus (Takara) were used total mRNA extraction for both HEK293T cells and HUDEP-2 cells according to the manufacturer’s instructions. Isolated HEK293T mRNA are sent directly to the company (Cipher Gene LLC) for RNA-seq. Isolated HUDEP-2 cells mRNA was reverse transcribed using HiScript II Q RT SuperMix (Vazyme). qPCR was performed on the QuantiStudio 3 real-time PCR system (ABI), and γ -globin mRNA expression levels were calculated by a percentage (γ/γ + β) in HUDEP-2(Δ ^G^ γ) cells. qPCR primers are listed in Supplementary Table 5.

Lentivirus production and transduction of cell lines. Lentivirus production was performed as previously described^27^. Briefly, HEK293T cells were seeded into a 10-cm dish 1 d before transfection. At approximately 85% confluency, cells were co-transfected with 10-μg transfer plasmid (Lenti ABE8e or Lenti hyABE), 5 μg pMD2.G and 7.5 μg psPAX2. Virus-containing supernatant was harvested at 48 h and 72 h after transfection. Supernatant was centrifuged at 4000 g for 10 min at 4 °C to precipitate cell debris, filtered by passing through a 0.45-mm low-protein binding membrane (Millipore) and then centrifuged at 25,000 g for 2.5 h at 4 °C to concentrate the lentivirus^27^.

#### Lentiviral titration

Virus stock was diluted via five serial ten-fold dilutions with DMEM (10% FBS). For each viral construct, 1 × 10^4^ HEK293T cells were first digested and suspended. Cells were spun down, resuspended with the diluted virus (100 μl) and seeded into 96-well plates. Control cells were resuspended with DMEM (10% FBS) only. Three days after transduction, cells were analyzed by checking the EGFP fluorescence via Fortessa Flow Cell Analyzer (BD Biosciences). Virus titration was calculated as follows: titer (TU per ml) = cell number × (%) EGFP × 10^3^ per virus stock volume (μl).

#### Zebrafish husbandary and breeding

Zebrafish (*Danio rerio*) of the AB strain were raised and maintained at 28 °C. About 50 pairs of male and female zebrafish (age from 4 months to 12 months) were used for crossing and generating embryos. Embryos were raised under a 14–10 h light-dark cycle in E3 medium, which consisted of: 5 mM NaCl, 0.17 mM KCl, 0.33 mM CaCl_2_, 0.33 mM MgSO_4_.

#### Preparation of mRNAs and sgRNAs and Microinjection in zebrafish

sgRNAs with the modification of phosphorothioation and methoxy group were synthesized by GenScript. (Nanjing, China) (Supplementary Table 4). mRNA preparation was performed as previously described^8^. Briefly, the T7 promoter was introduced into ABEs and dual base editor template by PCR using primers T7-mRNA (ABEmax/ABE8e/hyABE)-F/R (Supplementary Data 2). ABEs and dual base editor mRNA were transcribed in vitro using mMESSAGE mMACHINE T7 Kit (Invitrogen) and purified using a MEGAclear Kit (Invitrogen)^8^. Zebrafish embryo microinjection was performed as previously described^24^. A 2 nl mixture of ABEs mRNA (100 ng μl^−1^) and sgRNA (200 ng μl^−1^) or dual base editor mRNA (200 ng μl^−1^) and gRNA (200 ng μl^−1^) was co-injected into one-cell stage wild-type embryos. Injected embryos were incubated at 25 °C for 5 h and then transfer to 28 °C. Embryos that developed normally at 48 h were collected in groups. Genomic DNA was isolated using the QuickExtract™ DNA Extraction Solution (QE09050, Epicenter) according to the manufacturer’s instructions.

#### O-dianisitine staining

O-dianisidine staining was performed as previously described^23^.Briefly, embryos at 48 hpf were first anesthetized with Tricane and then fixed with 4% paraformaldehyde (PFA) at room temperature for 2 h. They were then washed three times with PBS (5 min each) to remove PFA. The embryos were then stained with 0.6 mg/mL o-dianisidine (Sigma Aldrich) in an o-dianisidine staining solution (40% ethanol, 0.65% H_2_O_2_, 10 mmol/L Na-Acetate) for 30 min in dark and followed by four PBS washeses (5 min each). Then, the embryos were incubated into a bleach solution (1% KOH, 3% H_2_O_2_) for 20 min to remove pigmentation. Images of stained embryos were taken immediately after rinsing with PBS. Finally, genotypes were confirmed in all samples by HTS.

#### Whole-mount in situ hybridization

WISH was carried out as previously described^28,29^. In brief, Digoxigenin (Roche)-labeled *hbael* probe was synthesized by T7 RNA polymerase (invitrogen) and used at a concentration of 2 ng/μl diluted with hybridization buffer. The hybridization was performed overnight at 65 °C. Hybridized probes were detected by 1:5000 dilution of anti-Digoxigenin-AP Fabfragment (Roche) and visualized by NBT/BCIP (Roche) substrate reacting at 37 °C.

#### High-throughput DNA sequencing and data analysis

On- and off-target genomic regions of interest were amplified by PCR with flanking high-throughput sequencing primer pairs listed in Supplementary Data 2, 3. PCR amplification was carried out with KOD-Plus-Neo DNA Polymerase according to the manufacturer’s instructions with 100–150 ng of genomic DNA as a template. And site-specific primers containing an adapter sequence (forward 5′-GGAGTGAGTACGGTGTGC-3′; backward 5′-GAGTTGGATGCTGGATGG-3′) at the 5′ end were used for PCR to prepare high-throughput sequencing (HTS) libraries. The products were then subjected to a second-round PCR using primers with different barcode sequences. Then, PCR products with different tags were pooled together for deep sequencing using the Illumina HiSeq platform. For the batch analysis of FASTQ files, the reference sequences were set to full-length and analyzed as previously described^5^.The alleles containing combined (C-to-T and A-to-G) or exclusive (only C-to-T or A-to-G) conversions and indels were quantified using BE-Analyzer^30^ or CRISPResso2^31^ or custom script in Supplementary Software.

#### Statistics and reproducibility

Three biologically independent replicates performed on different days were used to calculate means and SD unless stated otherwise. All bar plots and figures were generated using Prism 9.3 (GraphPad). An unpaired two-tailed Student’s t-test performed in GraphPad Prism 9.3 (GraphPad Software) was used to determine the significance of the differences between two groups. Specific *P* values are indicated in the figure captions. *P* < 0.05 was considered significant. RNA-seq data were analyzed using Trim Galore (version 0.6.6), STAR (version 2.7.1a), SAMtools (version 1.14), Picard MarkDuplicates module (version 2.23.9) software. FACS data was analyzed using FlowJo v.10.

### Reporting summary

Further information on research design is available in the Nature Portfolio Reporting Summary linked to this article.

## Supplementary information

Supplementary information

Reporting Summary

Description of Additional Supplementary Files

Supplementary Data 1

Supplementary Data 2

Supplementary Data 3

Supplementary Software

## Data Availability

The raw high-throughput sequencing data generated in this study have been deposited in the NCBI sequence Read Archive database under PRJNA820131, PRJNA820322, PRJNA899988, and PRJNA925829. RNA-seq data have been deposited in the NCBI sequence Read Archive database under accession code PRJNA818975. Plasmids used in this work will be available on Addgene (#196096, #196097, #196098, #196099, #196100, #196101, #196102). Source data are provided with this paper.

## Source data

Source Data

## Code availability

The custom script to analyze simultaneous A-to-G and C-to-T conversions induced by dual base editors is in https://zenodo.org/record/7635813#.Y-otL5PgQUs, and in the Supplementary Software file: there are no access restrictions.
