# Supplementary material for: Improving adenine and dual base editors through introduction of TadA-8e and Rad51DBD
Source: Nat Commun. 2023 Mar 3;14:1224. doi: 10.1038/s41467-023-36887-1 (PMC9984408; doi:10.1038/s41467-023-36887-1)
Supplement: Supplementary file 1 — Supplementary information [file 41467_2023_36887_MOESM1_ESM.pdf]

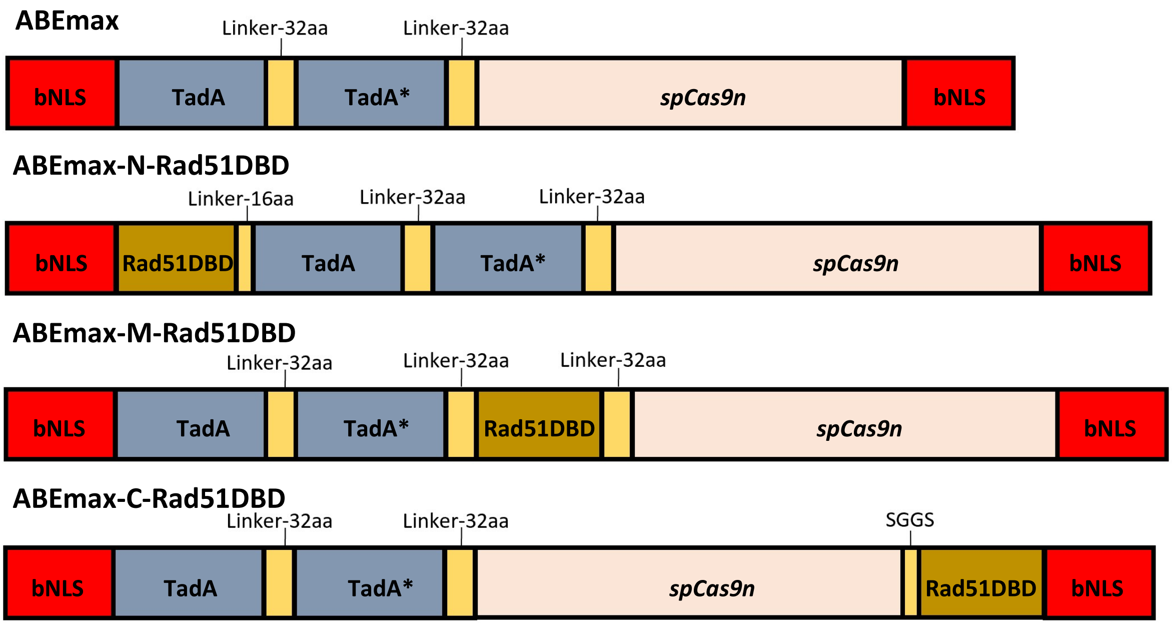

Supplementary Fig. 1 Schematics of the constructs with Rad51DBD fused to ABEmax. bNLS, bipartite nuclear localization signals; TadA, derived *E.coil* adenosine deaminase; TadA*, derived from evolved *E.coil* adenosine deaminase; spCas9n, Cas9 D10A; Rad51DBD, single-strand DNA-binding domain; Linkers are also shown.

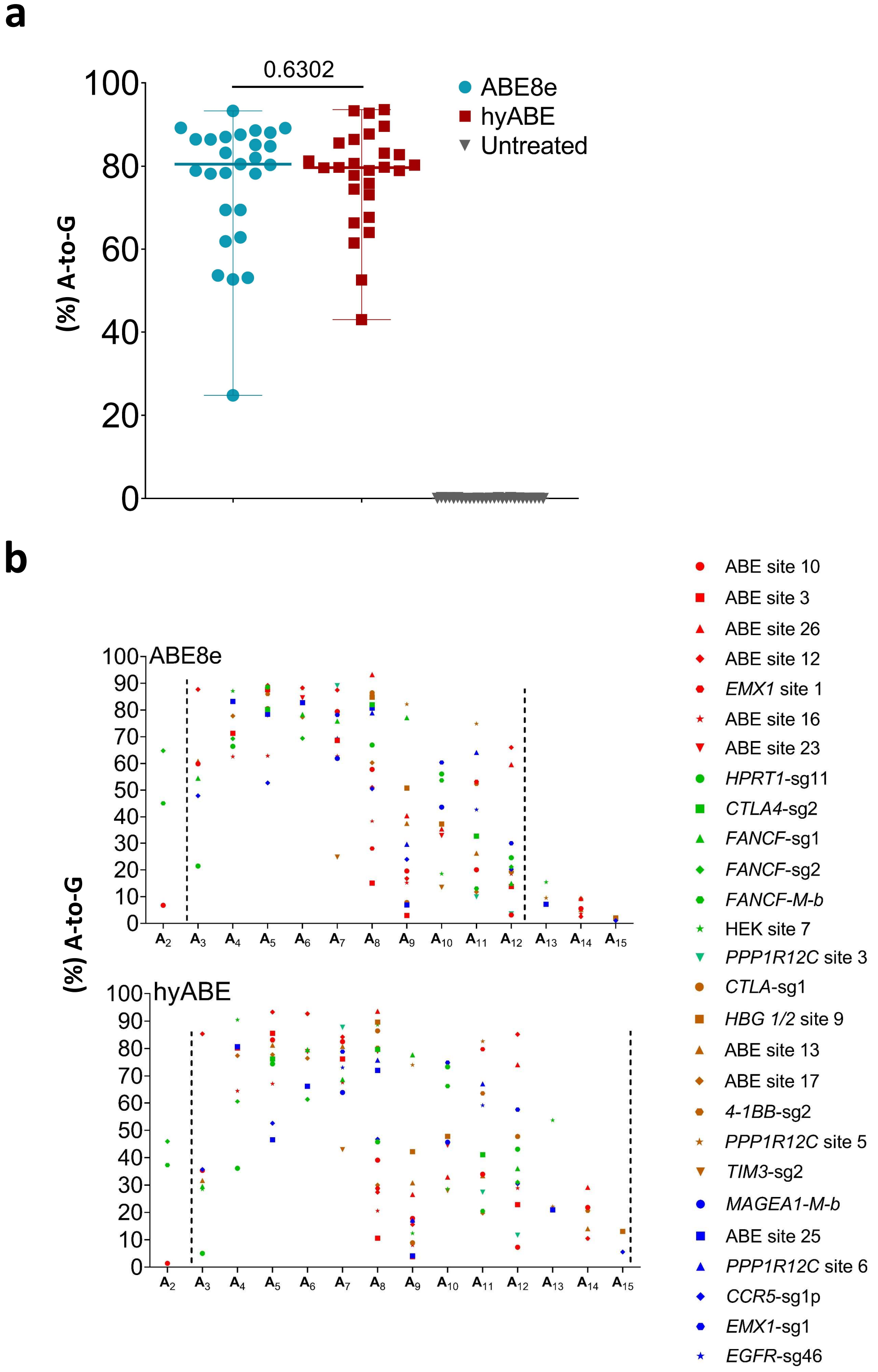

Supplementary Fig. 2 Comparison of the editing efficiency and editing window between ABE8e and hyABE. a, Comparison of A-to-G base editing efficiencies for only the most highly edited adenine induced by ABE8e and hyABE at 27 target sites in HEK293T cells. Each data point represents the average of the highest editing efficiency at each target site calculated from 3 independent experiments. Error bars and *P* value are derived from these 27 data points. *P* value was determined by paired two-sided Wilcoxon rank-sum test. **b,** Comparison of A-to-G base editing window of ABE8e and hyABE at 27 target sites in HEK293T cells. Data point represent means from three independent experiments. Source data are provided with this paper.

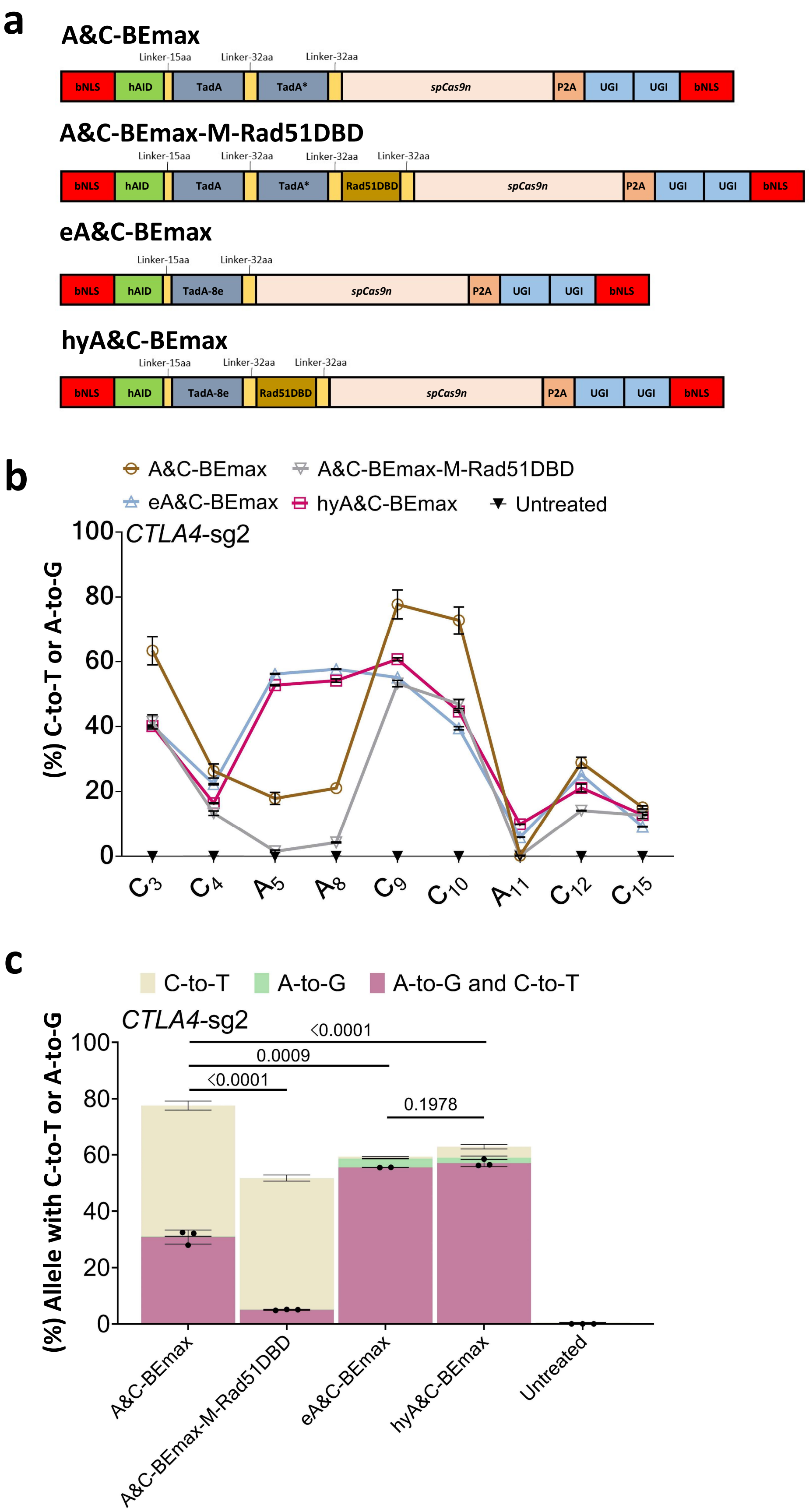

Supplementary Fig. 3 Screening of hyper A&C-BEmax. **a,** Schematics of the constructs with Rad51DBD fused to A&C-BEs. bNLS, bipartite nuclear localization signals; TadA, derived *E.coil* adenosine deaminase, TadA* or TadA-8e, derived from evolved *E.coil* adenosine deaminase; spCas9n, Cas9 D10A; UGI, Uracil DNA glycosylase inhibitor; Rad51DBD, single-strand DNA-binding domain; Linkers are also shown. **b,** The A-to-G or C-to-T editing efficiency of A&C-BEs at *CTLA4*-sg2 in HEK293T cells. Data are means ± SD (n = 3 independent experiments). **c,** Frequency of A/C simultaneous conversion induced by A&C-BEs at *CTLA4*-sg2 in HEK293T cells. Data are means ± SD (n = 3 independent experiments). All statistical testing was performed using two-tailed Student's t-test. Source data are provided with this paper.

A&C-BEmax vs. eA&C-BEmax

Site	p-value	t-value	Significance
PPP1R12C site 3 A2	0.3739	1.000	p>0.05
PPP1R12C site 3 C4	0.0005	10.24	***
PPP1R12C site 3 C6	0.1823	1.612	p>0.05
PPP1R12C site 3 A7	<0.0001	37.12	****
PPP1R12C site 3 C8	0.0002	12.53	***
PPP1R12C site 3 A11	0.0018	7.425	**
PPP1R12C site 3 A12	0.0075	5.000	**
PPP1R12C site 3 C13	0.0092	4.723	**
PPP1R12C site 3 C15	<0.0001	23.41	****
EGFR-sg2 C1	<0.0001	77.01	****
EGFR-sg2 C3	<0.0001	95.89	****
EGFR-sg2 C4	<0.0001	43.23	****
EGFR-sg2 C5	<0.0001	40.53	****
EGFR-sg2 A6	0.0239	3.546	*
EGFR-sg2 A7	<0.0001	22.35	****
EGFR-sg2 C8	<0.0001	42.25	****
EGFR-sg2 C9	<0.0001	70.15	****
EGFR-sg2 A10	0.0137	4.202	*
EGFR-sg2 A11	>0.9999	0.000	p>0.05
EGFR-sg2 C13	0.0001	15.36	***
EGFR-sg2 C15	0.0399	3.000	*
PPP1R12C site 15 A5	<0.0001	24.21	****
PPP1R12C site 15 A7	<0.0001	27.53	****
PPP1R12C site 15 C8	0.0033	6.283	**
PPP1R12C site 15 A9	<0.0001	71.80	****
PPP1R12C site 15 C14	0.0002	12.36	***
PPP1R12C site 15 C15	<0.0001	16.49	****
MAGEA1-M-b C2	0.0058	5.376	**
MAGEA1-M-b C6	<0.0001	17.89	****
MAGEA1-M-b A7	0.1376	1.853	p>0.05
MAGEA1-M-b C9	<0.0001	19.64	****
MAGEA1-M-b A10	<0.0001	24.73	****
MAGEA1-M-b A11	0.2302	1.414	p>0.05
HBB-sg2 C1	<0.0001	24.73	****
HBB-sg2 C5	<0.0001	27.98	****
HBB-sg2 C6	<0.0001	24.58	****
HBB-sg2 C7	<0.0001	24.28	****
HBB-sg2 C8	<0.0001	26.17	****
HBB-sg2 A9	<0.0001	16.10	****
HBB-sg2 C10	<0.0001	34.27	****
HBB-sg2 A11	<0.0001	21.11	****
HBB-sg2 C15	<0.0001	17.58	****
ABE site 25 A4	0.0003	12.08	***
ABE site 25 A5	0.0003	11.30	***
ABE site 25 A6	0.0002	12.58	***
ABE site 25 C7	0.0040	5.955	**
ABE site 25 A8	0.0001	15.16	***
ABE site 25 C12	0.0005	10.57	***
EMX1-sg6p C2	0.0264	3.437	*
EMX1-sg6p C4	0.1059	2.081	p>0.05
EMX1-sg6p C5	0.3761	0.9950	p>0.05
EMX1-sg6p A6	0.0019	7.322	**
EMX1-sg6p C7	0.2168	1.465	p>0.05
EMX1-sg6p C8	0.1123	2.029	p>0.05
FGF6-sg2 C2	0.0015	7.710	**
FGF6-sg2 A3	0.0001	15.37	***
FGF6-sg2 A8	0.0006	9.909	***
FGF6-sg2 A9	0.0005	10.55	***
FGF6-sg2 C15	<0.0001	32.55	****
FGF6-sg2 C18	<0.0001	89.57	****
EGFR-sg4 A2	0.0006	9.723	***
EGFR-sg4 A4	<0.0001	64.89	****
EGFR-sg4 C6	<0.0001	46.90	****
EGFR-sg4 A7	<0.0001	47.60	****
EGFR-sg4 A8	<0.0001	37.24	****
EGFR-sg4 A9	<0.0001	25.98	****
EGFR-sg4 C13	<0.0001	119.8	****
EGFR-sg4 C18	<0.0001	156.5	****
ABL-sg16 A2	0.0006	9.827	***
ABL-sg16 A6	0.0032	6.319	**
ABL-sg16 C10	0.0068	5.132	**
ABL-sg16 A11	<0.0001	17.20	****
ABL-sg16 A12	0.1583	1.732	p>0.05
ABL-sg16 C13	<0.0001	16.41	****
ABL-sg16 C14	0.0036	6.144	**
ABL-sg16 C17	0.0142	4.159	*
PD-1-sg3 A3	<0.0001	223.0	****
PD-1-sg3 C4	0.0016	24.71	**
PD-1-sg3 C5	0.0001	87.21	***
PD-1-sg3 C7	<0.0001	124.3	****
PD-1-sg3 A8	0.0001	95.26	***
PD-1-sg3 C10	0.0001	92.17	***
PD-1-sg3 C11	0.0005	46.17	***
PD-1-sg3 A12	0.0010	31.00	**
PD-1-sg3 C14	0.0044	15.00	**
RUNX1-sg3 A2	<0.0001	41.68	****
RUNX1-sg3 C3	<0.0001	21.24	****
RUNX1-sg3 C4	<0.0001	28.35	****
RUNX1-sg3 C5	<0.0001	21.31	****
RUNX1-sg3 A6	0.0099	4.613	**
RUNX1-sg3 C7	<0.0001	15.90	****
RUNX1-sg3 A8	0.0002	13.37	***
RUNX1-sg3 C12	<0.0001	21.74	****
RUNX1-sg3 C15	<0.0001	45.61	****
PTEN-sg1 A3	<0.0001	41.68	****
PTEN-sg1 C6	<0.0001	26.62	****
PTEN-sg1 A8	<0.0001	57.17	****
PTEN-sg1 C10	<0.0001	34.11	****
PTEN-sg1 A11	<0.0001	65.82	****
PTEN-sg1 A12	0.1161	2.000	p>0.05
PTEN-sg1 C13	<0.0001	56.06	****
EMX1 site 2 A3	0.0001	14.94	***
EMX1 site 2 C6	0.0068	5.129	**
EMX1 site 2 A7	0.0101	4.593	*
EMX1 site 2 C8	0.0030	6.440	**
EMX1 site 2 C9	0.0001	14.42	***
PPP1R12C site 11 C3	0.0012	12.28	**
PPP1R12C site 11 A4	<0.0001	28.27	****
PPP1R12C site 11 A6	0.0107	5.700	*
PPP1R12C site 11 C7	0.0002	20.69	***
PPP1R12C site 11 A8	0.0002	23.73	***
PPP1R12C site 11 C9	0.0002	24.26	***
PPP1R12C site 11 C14	0.0015	11.38	**
FANCF-sg1 A3	0.0001	25.49	***
FANCF-sg1 C4	0.0094	5.963	**
FANCF-sg1 C5	0.0005	16.18	***
FANCF-sg1 A6	0.0376	3.567	*
FANCF-sg1 A7	0.0003	18.45	***
FANCF-sg1 A9	<0.0001	34.41	****
FANCF-sg1 C11	0.0005	16.12	***
FANCF-sg1 C16	<0.0001	29.43	****
HBG 1/2 site 9 C1	0.0002	13.59	***
HBG 1/2 site 9 C2	<0.0001	28.78	****
HBG 1/2 site 9 C6	<0.0001	17.81	****
HBG 1/2 site 9 A8	<0.0001	19.91	****
HBG 1/2 site 9 C11	0.0001	15.31	***
HBG 1/2 site 9 C13	0.0002	13.45	***
HBG 1/2 site 9 C14	0.0005	10.45	***
HBG 1/2 site 9 C16	0.0001	14.84	***
HBG 1/2 site 9 C17	<0.0001	23.06	****
HBG 1/2 site 9 C18	0.0052	5.539	**
EGFR-sg46 C1	<0.0001	62.58	****
EGFR-sg46 C2	<0.0001	156.1	****
EGFR-sg46 C6	<0.0001	81.50	****
EGFR-sg46 A7	0.0001	15.19	***
EGFR-sg46 C9	<0.0001	104.5	****
EGFR-sg46 C10	<0.0001	39.19	****
EGFR-sg46 A11	<0.0001	36.68	****
EGFR-sg46 C16	<0.0001	50.00	****
RUNX1-sg5 A1	0.0006	9.827	***
RUNX1-sg5 A4	<0.0001	171.1	****
RUNX1-sg5 A5	<0.0001	37.24	****
RUNX1-sg5 C7	<0.0001	24.36	****
RUNX1-sg5 A8	<0.0001	25.03	****
RUNX1-sg5 C9	<0.0001	17.31	****
TIM3-sg1 C3	<0.0001	28.68	****
TIM3-sg1 A5	<0.0001	21.64	****
TIM3-sg1 C6	<0.0001	23.84	****
TIM3-sg1 A7	<0.0001	27.42	****
TIM3-sg1 C8	<0.0001	22.02	****
TIM3-sg1 C9	<0.0001	23.37	****
TIM3-sg1 C10	<0.0001	22.52	****
TIM3-sg1 C11	<0.0001	32.29	****

A&C-BEmax vs. hyA&C-BEmax

Site	p-value	t-value	Significance
PPP1R12C site 3 A2	0.3739	1.000	p>0.05
PPP1R12C site 3 C4	0.0022	7.002	**
PPP1R12C site 3 C6	0.0052	5.534	**
PPP1R12C site 3 A7	<0.0001	32.00	****
PPP1R12C site 3 C8	0.0050	5.585	**
PPP1R12C site 3 A11	0.0358	3.111	*
PPP1R12C site 3 A12	0.1550	1.750	p>0.05
PPP1R12C site 3 C13	0.0226	3.606	*
PPP1R12C site 3 C15	0.0002	13.26	***
EGFR-sg2 C1	<0.0001	36.49	****
EGFR-sg2 C3	<0.0001	58.42	****
EGFR-sg2 C4	<0.0001	37.01	****
EGFR-sg2 C5	<0.0001	39.19	****
EGFR-sg2 A6	0.0018	7.419	**
EGFR-sg2 A7	<0.0001	34.99	****
EGFR-sg2 C8	0.0043	5.850	**
EGFR-sg2 C9	0.0042	5.888	**
EGFR-sg2 A10	<0.0001	33.07	****
EGFR-sg2 A11	0.5185	0.7071	p>0.05
EGFR-sg2 C13	0.0003	11.83	***
EGFR-sg2 C15	0.0147	4.111	*
PPP1R12C site 15 A5	<0.0001	52.26	****
PPP1R12C site 15 A7	<0.0001	20.09	****
PPP1R12C site 15 C8	0.1701	1.671	p>0.05
PPP1R12C site 15 A9	<0.0001	72.45	****
PPP1R12C site 15 C14	0.0005	10.21	***
PPP1R12C site 15 C15	0.0004	10.70	***
MAGEA1-M-b C2	0.0100	4.608	**
MAGEA1-M-b C6	0.0002	13.45	***
MAGEA1-M-b A7	0.0074	5.011	**
MAGEA1-M-b C9	<0.0001	16.51	****
MAGEA1-M-b A10	0.0001	15.03	***
MAGEA1-M-b A11	0.2302	1.414	p>0.05
HBB-sg2 C1	0.0003	11.57	***
HBB-sg2 C5	<0.0001	26.29	****
HBB-sg2 C6	<0.0001	26.48	****
HBB-sg2 C7	<0.0001	22.24	****
HBB-sg2 C8	<0.0001	24.65	****
HBB-sg2 A9	0.0005	10.07	***
HBB-sg2 C10	<0.0001	33.59	****
HBB-sg2 A11	<0.0001	52.97	****
HBB-sg2 C15	0.0002	12.70	***
ABE site 25 A4	<0.0001	58.26	****
ABE site 25 A5	<0.0001	16.44	****
ABE site 25 A6	0.0003	12.04	***
ABE site 25 C7	0.0006	9.989	***
ABE site 25 A8	<0.0001	41.35	****
ABE site 25 C12	0.0001	15.13	***
EMX1-sg6p C2	0.0317	3.239	*
EMX1-sg6p C4	0.1335	1.879	p>0.05
EMX1-sg6p C5	0.9719	0.03742	p>0.05
EMX1-sg6p A6	0.0031	6.402	**
EMX1-sg6p C7	0.6020	0.5655	p>0.05
EMX1-sg6p C8	0.5469	0.6573	p>0.05
FGF6-sg2 C2	0.0007	9.355	***
FGF6-sg2 A3	<0.0001	17.59	****
FGF6-sg2 A8	0.0009	8.848	***
FGF6-sg2 A9	0.2071	1.504	p>0.05
FGF6-sg2 C15	<0.0001	22.06	****
FGF6-sg2 C18	<0.0001	118.4	****
EGFR-sg4 A1	0.3739	1.000	p>0.05
EGFR-sg4 A2	0.0012	8.285	**
EGFR-sg4 A4	<0.0001	25.52	****
EGFR-sg4 C6	0.0001	15.41	***
EGFR-sg4 A7	0.0001	31.11	***
EGFR-sg4 A8	0.0004	10.97	***
EGFR-sg4 A9	0.0029	6.500	**
EGFR-sg4 C13	<0.0001	16.38	****
EGFR-sg4 C18	<0.0001	66.60	****
ABL-sg16 A2	0.1161	2.000	p>0.05
ABL-sg16 A6	0.0031	6.373	**
ABL-sg16 C10	0.0143	4.146	*
ABL-sg16 A11	<0.0001	17.05	****
ABL-sg16 C13	0.0002	13.91	***
ABL-sg16 C14	0.0046	5.731	**
ABL-sg16 C17	0.0012	8.133	**
PD-1-sg3 A3	0.0007	14.33	***
PD-1-sg3 C4	0.0320	3.800	*
PD-1-sg3 C5	0.0100	5.834	*
PD-1-sg3 C7	<0.0001	174.6	****
PD-1-sg3 A8	<0.0001	542.2	****
PD-1-sg3 C10	<0.0001	39.56	****
PD-1-sg3 C11	0.0079	6.337	**
PD-1-sg3 A12	0.0001	27.38	***
PD-1-sg3 C14	0.0001	24.52	***
RUNX1-sg3 A2	<0.0001	42.09	****
RUNX1-sg3 C3	<0.0001	40.71	****
RUNX1-sg3 C4	<0.0001	53.08	****
RUNX1-sg3 C5	<0.0001	139.9	****
RUNX1-sg3 A6	<0.0001	74.10	****
RUNX1-sg3 C7	<0.0001	39.04	****
RUNX1-sg3 A8	<0.0001	173.2	****
RUNX1-sg3 C12	<0.0001	155.3	****
RUNX1-sg3 C15	<0.0001	39.24	****
PTEN-sg1 A3	<0.0001	28.64	****
PTEN-sg1 C6	<0.0001	22.80	****
PTEN-sg1 A8	<0.0001	52.02	****
PTEN-sg1 C10	<0.0001	35.07	****
PTEN-sg1 A11	<0.0001	158.0	****
PTEN-sg1 A12	0.0013	8.000	**
PTEN-sg1 C13	<0.0001	49.37	****
EMX1 site 2 A3	0.0003	11.60	***
EMX1 site 2 C6	0.0021	7.059	**
EMX1 site 2 A7	0.0002	13.64	***
EMX1 site 2 C8	0.0024	6.826	**
EMX1 site 2 C9	0.0002	12.57	***
PPP1R12C site 11 C3	<0.0001	40.11	****
PPP1R12C site 11 A4	0.0004	17.13	***
PPP1R12C site 11 A6	0.0159	4.937	*
PPP1R12C site 11 C7	0.0181	4.709	*
PPP1R12C site 11 A8	0.0016	11.08	**
PPP1R12C site 11 C9	0.0031	8.747	**
PPP1R12C site 11 C14	0.0125	5.389	*
FANCF-sg1 C4	0.0016	7.616	**
FANCF-sg1 C5	0.0020	7.216	**
FANCF-sg1 A6	0.0008	9.140	***
FANCF-sg1 A7	0.0151	4.082	*
FANCF-sg1 A9	<0.0001	92.98	****
FANCF-sg1 C11	0.0005	10.11	***
FANCF-sg1 C16	0.0101	4.589	*
HBG 1/2 site 9 C1	<0.0001	35.26	****
HBG 1/2 site 9 C2	<0.0001	92.86	****
HBG 1/2 site 9 C6	0.0001	15.05	***
HBG 1/2 site 9 A8	<0.0001	29.82	****
HBG 1/2 site 9 C11	0.0002	12.77	***
HBG 1/2 site 9 C13	0.0002	12.66	***
HBG 1/2 site 9 C14	0.0008	9.250	***
HBG 1/2 site 9 C16	<0.0001	15.66	****
HBG 1/2 site 9 C17	<0.0001	22.14	****
HBG 1/2 site 9 C18	0.0018	7.359	**
EGFR-sg46 C1	<0.0001	65.41	****
EGFR-sg46 C2	<0.0001	36.43	****
EGFR-sg46 C6	0.0005	10.39	***
EGFR-sg46 A7	0.0011	8.441	**
EGFR-sg46 C9	0.0005	10.40	***
EGFR-sg46 C10	<0.0001	19.88	****
EGFR-sg46 A11	<0.0001	30.67	****
EGFR-sg46 C16	<0.0001	47.00	****
RUNX1-sg5 A1	0.0013	8.000	**
RUNX1-sg5 A4	<0.0001	28.93	****
RUNX1-sg5 A5	0.0005	10.52	***
RUNX1-sg5 C7	0.0049	5.625	**
RUNX1-sg5 A8	<0.0001	19.27	****
RUNX1-sg5 C9	0.0036	6.123	**
TIM3-sg1 C3	<0.0001	23.74	****
TIM3-sg1 A5	<0.0001	17.79	****
TIM3-sg1 C6	0.0001	14.61	***
TIM3-sg1 A7	<0.0001	23.40	****
TIM3-sg1 C8	0.0002	13.14	***
TIM3-sg1 C9	0.0001	14.06	***
TIM3-sg1 C10	0.0002	13.49	***
TIM3-sg1 C11	<0.0001	21.20	****

eA&C-BEmax vs. hyA&C-BEmax

Site	p-value	t-value	Significance
PPP1R12C site 3 A2	>0.9999	1.000	p>0.05
PPP1R12C site 3 C4	0.2458	1.359	p>0.05
PPP1R12C site 3 C6	0.3717	1.005	p>0.05
PPP1R12C site 3 A7	0.0760	2.380	p>0.05
PPP1R12C site 3 C8	0.1452	1.806	p>0.05
PPP1R12C site 3 A11	0.9001	0.1336	p>0.05
PPP1R12C site 3 A12	0.6530	0.4851	p>0.05
PPP1R12C site 3 C13	0.1000	2.132	p>0.05
PPP1R12C site 3 C15	0.2105	1.490	p>0.05
EGFR-sg2 C1	0.0006	9.849	***
EGFR-sg2 C3	<0.0001	18.41	****
EGFR-sg2 C4	0.0002	13.46	***
EGFR-sg2 C5	0.0008	9.171	***
EGFR-sg2 A6	0.0406	2.983	*
EGFR-sg2 A7	0.2695	1.281	p>0.05
EGFR-sg2 C8	0.1255	1.932	p>0.05
EGFR-sg2 C9	0.1365	1.859	p>0.05
EGFR-sg2 A10	0.0517	2.744	p>0.05
EGFR-sg2 A11	0.5185	0.7071	p>0.05
EGFR-sg2 C13	0.0105	4.540	*
EGFR-sg2 C15	0.0161	4.000	*
PPP1R12C site 15 A5	0.0078	4.939	**
PPP1R12C site 15 A7	0.0002	12.62	***
PPP1R12C site 15 C8	0.0014	7.903	**
PPP1R12C site 15 A9	0.0271	3.408	*
PPP1R12C site 15 C14	0.0021	7.089	**
PPP1R12C site 15 C15	0.0066	5.185	**
MAGEA1-M-b C2	0.7096	0.4000	p>0.05
MAGEA1-M-b C6	0.0004	11.16	***
MAGEA1-M-b A7	0.0627	2.560	p>0.05
MAGEA1-M-b C9	0.0007	9.338	***
MAGEA1-M-b A10	0.2005	1.531	p>0.05
MAGEA1-M-b A11	>0.9999	0.000	p>0.05
HBB-sg2 C1	0.0117	4.400	*
HBB-sg2 C5	0.0007	9.507	***
HBB-sg2 C6	0.0127	4.297	*
HBB-sg2 C7	0.0006	10.05	***
HBB-sg2 C8	0.0009	8.982	***
HBB-sg2 A9	0.0138	4.186	*
HBB-sg2 C10	0.0007	9.468	***
HBB-sg2 A11	<0.0001	30.25	****
HBB-sg2 C15	<0.0001	15.59	****
ABE site 25 A4	0.0535	2.710	p>0.05
ABE site 25 A5	0.0007	9.486	***
ABE site 25 A6	0.0005	10.44	***
ABE site 25 C7	0.2073	1.503	p>0.05
ABE site 25 A8	0.0862	2.265	p>0.05
ABE site 25 C12	0.0563	2.662	p>0.05
EMX1-sg6p C2	0.0945	2.182	p>0.05
EMX1-sg6p C4	0.0931	2.196	p>0.05
EMX1-sg6p C5	0.1483	1.788	p>0.05
EMX1-sg6p A6	0.4022	0.9362	p>0.05
EMX1-sg6p C7	0.0847	2.281	p>0.05
EMX1-sg6p C8	0.1067	2.075	p>0.05
FGF6-sg2 C2	0.0794	2.339	p>0.05
FGF6-sg2 A3	0.1417	1.827	p>0.05
FGF6-sg2 A8	0.0672	2.494	p>0.05
FGF6-sg2 A9	0.1439	1.814	p>0.05
FGF6-sg2 C15	0.7719	0.3103	p>0.05
FGF6-sg2 C18	0.0007	9.470	***
EGFR-sg4 A1	0.0004	11.00	***
EGFR-sg4 A2	0.0132	4.243	*
EGFR-sg4 A4	0.0001	14.97	***
EGFR-sg4 C6	0.0005	10.41	***
EGFR-sg4 A7	0.0689	2.470	p>0.05
EGFR-sg4 A8	0.0008	9.045	***
EGFR-sg4 A9	0.3911	0.9608	p>0.05
EGFR-sg4 C13	0.0014	7.844	**
EGFR-sg4 C18	0.0769	2.369	p>0.05
ABL-sg16 A2	0.0006	9.839	***
ABL-sg16 A6	0.3381	1.087	p>0.05
ABL-sg16 C10	0.0621	2.568	p>0.05
ABL-sg16 A11	0.0169	3.941	*
ABL-sg16 A12	>0.9999	0.000	p>0.05
ABL-sg16 C13	0.0006	9.960	***
ABL-sg16 C14	0.6640	0.4682	p>0.05
ABL-sg16 C17	0.0837	2.291	p>0.05
PD-1-sg3 A3	<0.0001	31.82	****
PD-1-sg3 C4	0.5091	0.7474	p>0.05
PD-1-sg3 C5	0.1430	1.973	p>0.05
PD-1-sg3 C7	<0.0001	68.01	****
PD-1-sg3 A8	<0.0001	109.9	****
PD-1-sg3 C10	0.0003	19.88	***
PD-1-sg3 C11	0.4494	0.8676	p>0.05
PD-1-sg3 A12	0.0003	19.18	***
PD-1-sg3 C14	0.0002	23.33	***
RUNX1-sg3 A2	0.0234	3.570	*
RUNX1-sg3 C3	0.0083	4.853	**
RUNX1-sg3 C4	0.0046	5.723	**
RUNX1-sg3 C5	0.0116	4.410	*
RUNX1-sg3 A6	0.0111	4.464	*
RUNX1-sg3 C7	0.0107	4.512	*
RUNX1-sg3 A8	0.1710	1.666	p>0.05
RUNX1-sg3 C12	0.0019	7.244	**
RUNX1-sg3 C15	0.0002	12.73	***
PTEN-sg1 A3	<0.0001	31.03	****
PTEN-sg1 C6	0.0037	6.062	**
PTEN-sg1 A8	0.8559	0.1936	p>0.05
PTEN-sg1 C10	0.0016	7.603	**
PTEN-sg1 A11	<0.0001	22.00	****
PTEN-sg1 A12	0.0132	4.243	*
PTEN-sg1 C13	<0.0001	17.52	****
EMX1 site 2 A3	0.0014	7.895	**
EMX1 site 2 C6	0.3666	1.017	p>0.05
EMX1 site 2 A7	0.3514	1.054	p>0.05
EMX1 site 2 C8	0.1092	2.054	p>0.05
EMX1 site 2 C9	0.0880	2.246	p>0.05
PPP1R12C site 11 C3	0.0039	6.008	**
PPP1R12C site 11 A4	<0.0001	22.37	****
PPP1R12C site 11 A6	0.0371	3.075	*
PPP1R12C site 11 C7	0.0053	5.516	**
PPP1R12C site 11 A8	0.0605	2.593	p>0.05
PPP1R12C site 11 C9	0.0094	4.686	**
PPP1R12C site 11 C14	0.0114	4.427	*
FANCF-sg1 A3	0.0002	22.81	***
FANCF-sg1 C4	0.7888	2.2928	p>0.05
FANCF-sg1 C5	0.0114	5.580	*
FANCF-sg1 A6	0.0072	6.565	**
FANCF-sg1 A7	0.0011	12.55	**
FANCF-sg1 A9	0.5993	0.5855	p>0.05
FANCF-sg1 C11	0.0078	6.368	**
FANCF-sg1 C16	0.9290	0.09682	p>0.05
HBG 1/2 site 9 C1	0.0001	15.10	***
HBG 1/2 site 9 C2	0.0018	7.416	**
HBG 1/2 site 9 C6	0.0346	3.149	*
HBG 1/2 site 9 A8	0.3014	1.186	p>0.05
HBG 1/2 site 9 C11	0.0691	2.467	p>0.05
HBG 1/2 site 9 C13	0.0274	3.395	*
HBG 1/2 site 9 C14	0.0257	3.464	*
HBG 1/2 site 9 C16	0.0572	2.646	p>0.05
HBG 1/2 site 9 C17	0.0065	5.196	**
HBG 1/2 site 9 C18	0.0257	3.464	*
EGFR-sg46 C1	0.0048	5.657	**
EGFR-sg46 C2	0.0114	4.435	*
EGFR-sg46 C6	0.0038	6.048	**
EGFR-sg46 A7	0.2840	1.236	p>0.05
EGFR-sg46 C9	0.0032	6.305	**
EGFR-sg46 C10	0.0074	5.008	**
EGFR-sg46 A11	0.0004	11.26	***
RUNX1-sg5 A1	0.0031	6.364	**
RUNX1-sg5 A4	<0.0001	46.97	****
RUNX1-sg5 A5	<0.0001	32.24	****
RUNX1-sg5 C7	0.0019	7.247	**
RUNX1-sg5 A8	0.6977	0.4176	p>0.05
RUNX1-sg5 C9	0.0026	6.719	**
TIM3-sg1 C3	0.0004	11.07	***
TIM3-sg1 A5	0.0066	0.0066	**
TIM3-sg1 C6	0.0007	9.377	***
TIM3-sg1 A7	0.1263	1.926	p>0.05
TIM3-sg1 C8	0.0004	10.68	***
TIM3-sg1 C9	0.0002	12.42	***
TIM3-sg1 C10	0.0006	9.871	***
TIM3-sg1 C11	0.0002	12.59	***

Supplementary Fig. 4 Unpaired two-sided t-test p-values and t-values for A&C-BEs editing at all tested target sites. Unpaired two-sided t-test p-values and t-values for A&C-BEmax, eA&C-BEmax and hyA&C-BEmax editing at 20 target sites in human cells. ns (not significant), $P \geq 0.05$; *$P < 0.05$; **$P < 0.01$; ***$P < 0.001$; ****$P < 0.0001$. Data are means ± SD (n = 3 independent experiments).

a

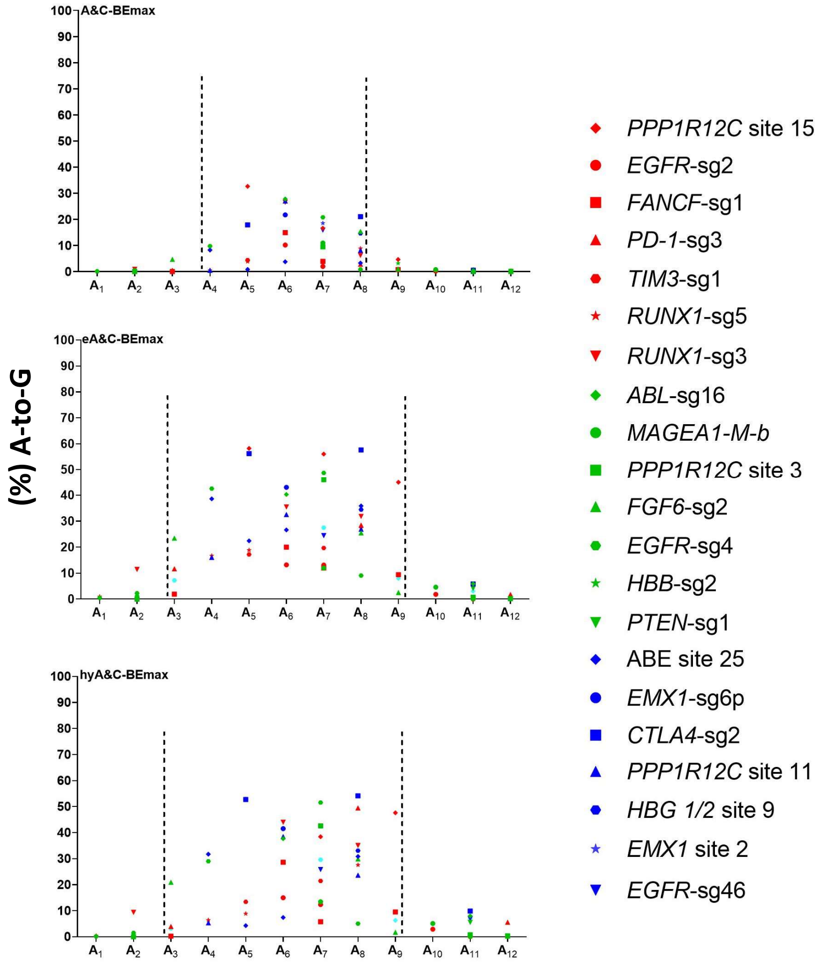

b

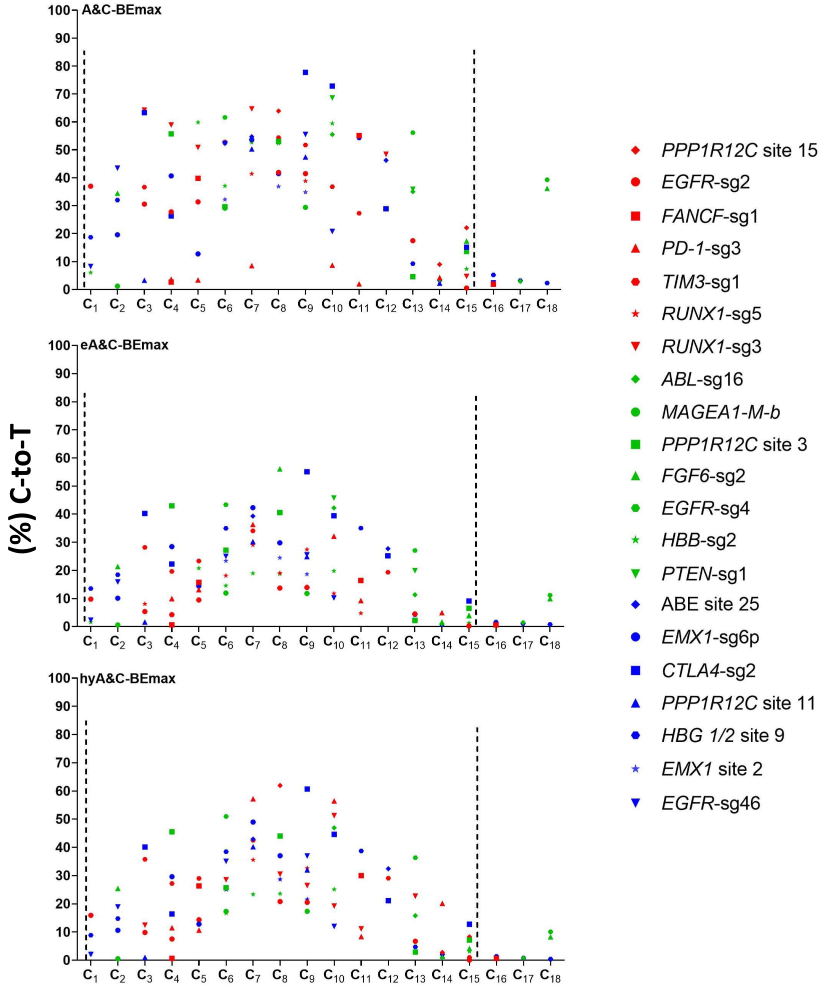

Supplementary Fig. 5 Comparison of the editing window A&C-BEmax, eA&C-BEmax and hyA&C-BEmax. a, Comparison of A-to-G base editing window of A&C-BEmax, eA&C-BEmax and hyA&C-BEmax at 21 target sites in HEK293T cells. Data points represent means from three independent experiments. **b,** Comparison of C-to-T base editing window of A&C-BEmax, eA&C-BEmax and hyA&C-BEmax at 21 target sites in HEK293T cells. Data point represent means from three independent experiments. Source data are provided with this paper.

a

A&C-BEmax vs. eA&C-BEmax				A&C-BEmax vs. hyA&C-BEmax				eA&C-BEmax vs. hyA&C-BEmax			
Site	p-value	t-value	Significance	Site	p-value	t-value	Significance	Site	p-value	t-value	Significance
A1	0.0002	5.677	***	A1	0.0237	2.666	*	A1	0.0031	3.868	***
A2	0.0236	2.432	*	A2	0.0466	2.108	*	A2	0.6222	0.4998	p>0.05
A3	0.0010	3.885	***	A3	0.0457	2.124	*	A3	0.2036	1.314	p>0.05
A4	<0.0001	5.689	****	A4	0.0046	3.167	**	A4	0.0618	1.968	p>0.05
A5	0.0014	3.569	**	A5	0.0369	2.191	*	A5	0.4024	0.8508	p>0.05
A6	0.0003	3.930	***	A6	0.0021	3.303	**	A6	0.9452	0.06918	p>0.05
A7	<0.0001	5.275	****	A7	<0.0001	4.968	****	A7	0.5359	0.6233	p>0.05
A8	<0.0001	7.700	****	A8	<0.0001	7.519	****	A8	0.5193	0.6490	p>0.05
A9	0.0125	2.733	*	A9	0.0200	2.509	*	A9	0.9459	0.06863	p>0.05
A10	0.0019	4.184	**	A10	<0.0001	6.745	****	A10	0.3633	0.9525	p>0.05
A11	<0.0001	5.508	****	A11	<0.0001	5.901	****	A11	0.0416	2.098	*
A12	0.0888	1.789	p>0.05	A12	0.0552	2.030	p>0.05	A12	0.1484	1.500	p>0.05

b

A&C-BEmax vs. eA&C-BEmax				A&C-BEmax vs. hyA&C-BEmax				eA&C-BEmax vs. hyA&C-BEmax			
Site	p-value	t-value	Significance	Site	p-value	t-value	Significance	Site	p-value	t-value	Significance
C1	0.0138	2.675	*	C1	0.0202	2.504	*	C1	0.8350	0.2108	p>0.05
C2	0.0070	2.912	**	C2	0.0126	2.664	*	C2	0.7980	0.2584	p>0.05
C3	0.0007	3.833	***	C3	0.0040	3.142	**	C3	0.4042	0.8474	p>0.05
C4	0.0416	2.113	*	C4	0.0356	2.177	*	C4	0.9591	0.05167	p>0.05
C5	0.0011	3.605	**	C5	0.0058	2.952	**	C5	0.1303	1.533	p>0.05
C6	<0.0001	5.723	****	C6	0.0002	4.057	***	C6	0.1156	1.604	p>0.05
C7	0.0004	3.868	***	C7	0.1167	1.605	p>0.05	C7	0.0035	3.106	**
C8	<0.0001	5.411	****	C8	0.0005	3.798	***	C8	0.1524	1.459	p>0.05
C9	<0.0001	6.194	****	C9	0.0003	3.904	***	C9	0.0266	2.292	*
C10	0.0023	3.281	**	C10	0.0692	1.869	p>0.05	C10	0.0948	1.714	p>0.05
C11	0.0174	2.605	*	C11	0.0479	2.101	*	C11	0.3906	0.8776	p>0.05
C12	0.0003	4.765	***	C12	0.0014	3.855	**	C12	0.1453	1.536	p>0.05
C13	0.0044	3.050	**	C13	0.0334	2.218	*	C13	0.3680	0.9125	p>0.05
C14	0.0055	3.075	**	C14	0.7084	0.3781	p>0.05	C14	0.1406	1.524	p>0.05
C15	<0.0001	4.671	****	C15	0.0014	3.425	**	C15	0.1148	1.613	p>0.05
C16	0.0022	3.688	**	C16	0.0008	4.107	***	C16	0.7220	0.3626	p>0.05
C17	<0.0001	9.183	****	C17	<0.0001	16.40	****	C17	0.0128	3.024	*
C18	0.0080	3.028	**	C18	0.0053	3.221	**	C18	0.6366	0.4817	p>0.05

c

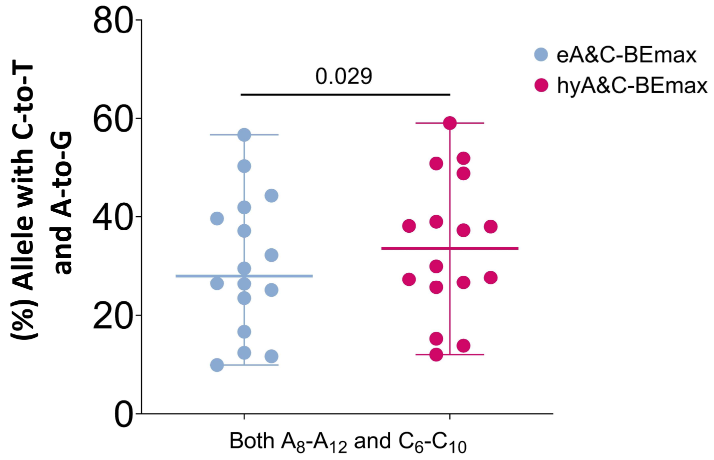

Supplementary Fig. 6 Comparison of the editing efficiency among A&C-BEmax, eA&C-BEmax and hyA&C-BEmax. a, Unpaired two-sided t-test p-values and t-values for A&C-BEmax, eA&C-BEmax and hyA&C-BEmax average A-to-G base editing at every protospacer position across 21 target sites in HEK293T cells. ns (not significant), $P \geq 0.05$; *$P < 0.05$; **$P < 0.01$; ***$P < 0.001$; ****$P < 0.0001$. Data are means ± SD (n = 3 independent experiments). **b,** Unpaired two-sided t-test p-values and t-values for A&C-BEmax, eA&C-BEmax and hyA&C-BEmax average C-to-T base editing at every protospacer position across 21 target sites in HEK293T cells. ns (not significant), $P \geq 0.05$; *$P < 0.05$; **$P < 0.01$; ***$P < 0.001$; ****$P < 0.0001$. Data are means ± SD (n = 3 independent experiments). **c,** The simultaneous A-to-G and C-to-T editing efficiency induced by eA&C-BEmax and hyA&C-BEmax when editable As were at position A_8-A_{12} and editable Cs were at position C_6-C_{10} at 16 of 21 endogenous target sites in HEK293T cells. Each data point represents average conversion frequency at each target site calculated from 3 independent experiments. All statistical testing was performed using paired two-sided Wilcoxon rank-sum. Source data are provided with this paper

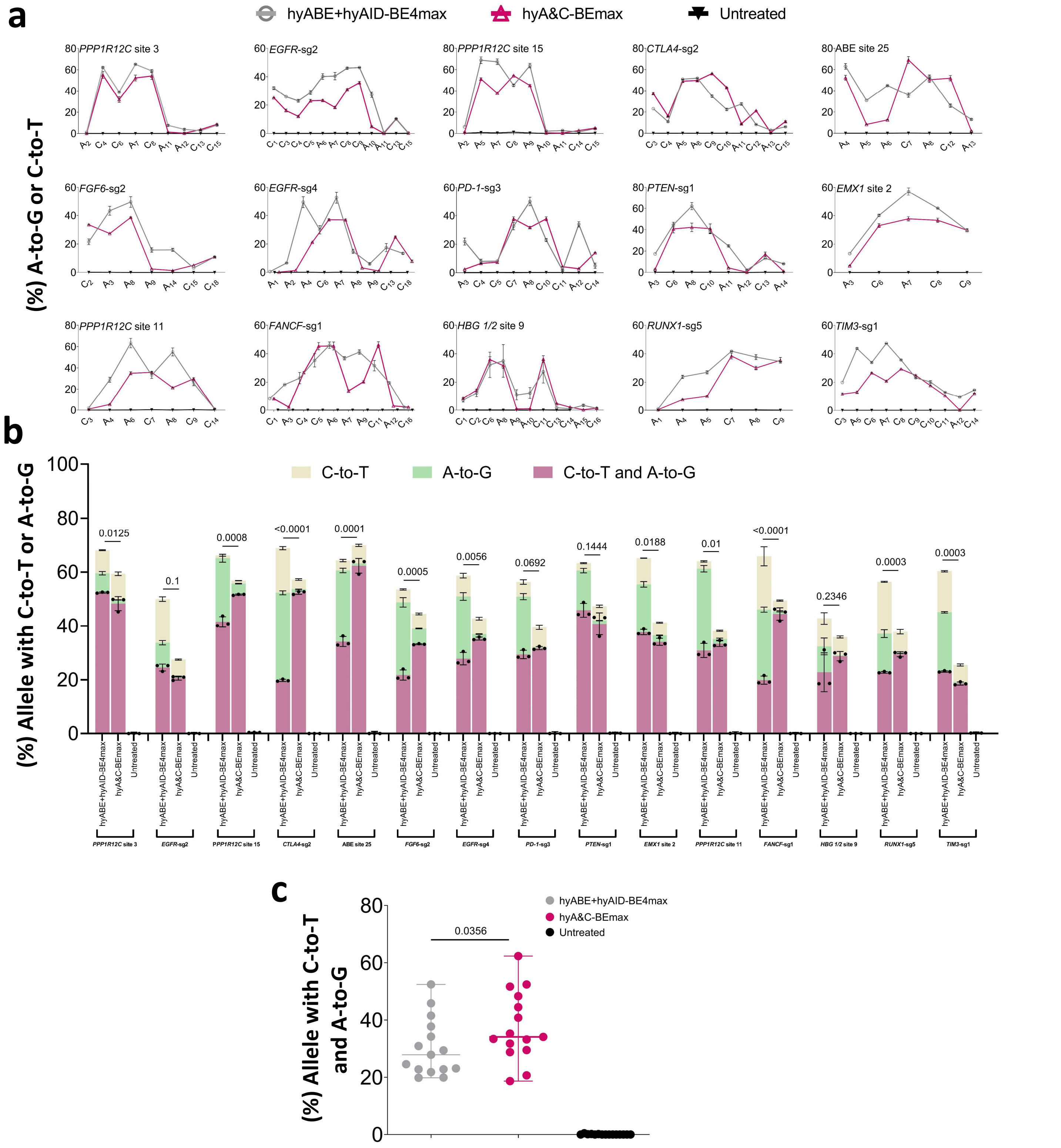

Supplementary Fig. 7 Comparison of the editing efficiency induced by hyA&C-BEmax and the mixture of hyABE and hyAID-BE4max. a, The A-to-G or C-to-T editing efficiency of hyA&C-BEmax and the mixture of hyABE and hyAID-BE4max was examined at 15 endogenous target sites in HEK293T cells. Data are means ± SD (n = 3 independent experiments). **b,** The allele with A-to-G or C-to-T efficiency of hyA&C-BEmax and the mixture of hyABE and hyAID-BE4max at 15 endogenous target sites in HEK293T cells. Data are means ± SD (n = 3 independent experiments). **c,** Frequency of A/C simultaneous conversion induced by hyA&C-BEmax and the mixture of hyABE and hyAID-BE4max at 15 endogenous genomic loci. Each data point represents average conversion frequency at each target site calculated from 3 independent experiments. For **b**, statistical testing was performed using two-tailed Student's t test. For **c**, statistical testing was performed using paired two-sided Wilcoxon rank-sum. Source data are provided with this paper.

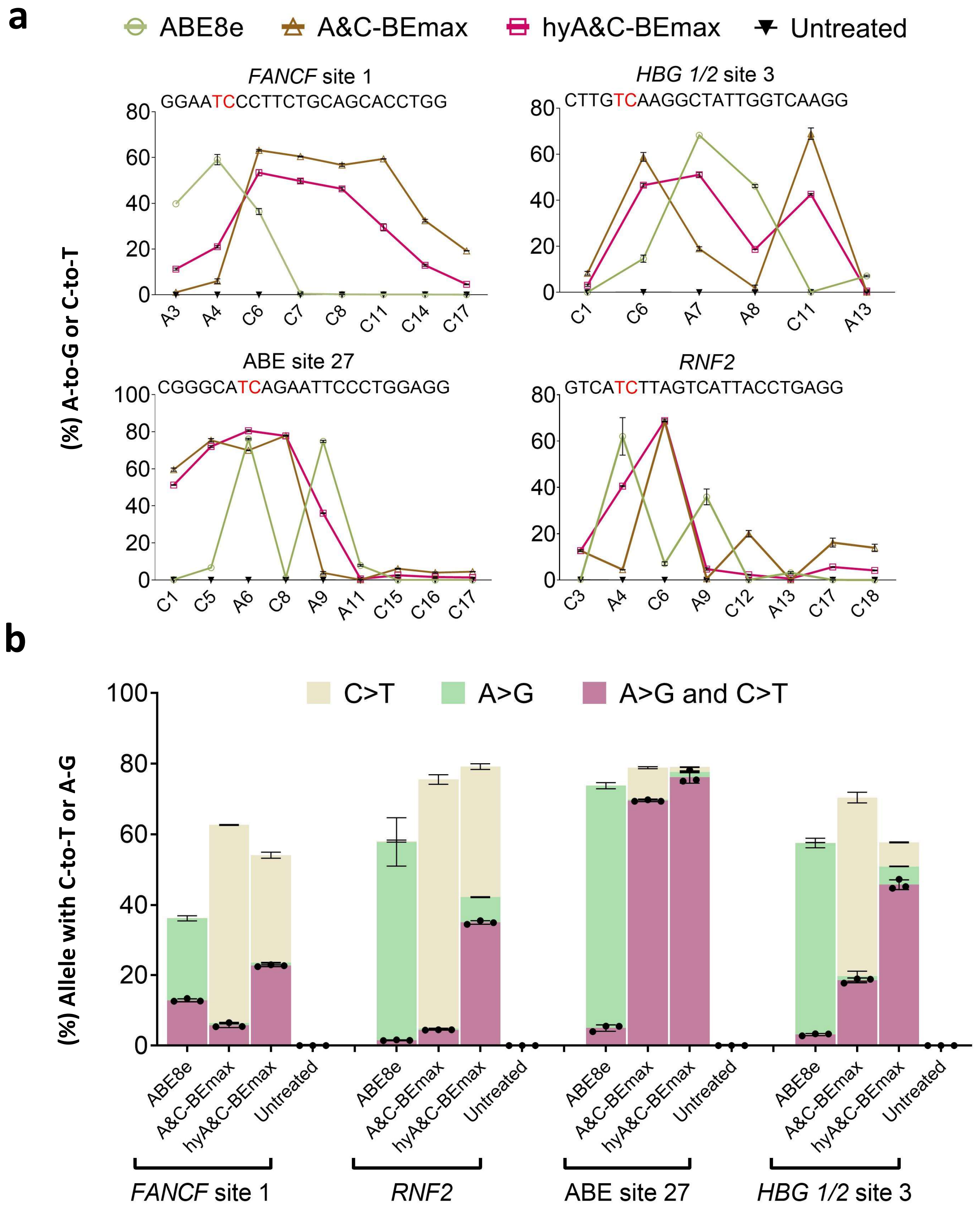

Supplementary Fig. 8 Characterization of hyA&C-BEmax in HEK293T cells. **a,** The A-to-G or C-to-T editing efficiency of ABE8e, A&C-BEmax or hyA&C-BEmax at 4 endogenous target sites containing TC motif in HEK293T cells. Data are means ± SD (n = 3 independent experiments). **b,** The allele with A-to-G or C-to-T efficiency of ABE8e, A&C-BEmax or hyA&C-BEmax at 4 endogenous target sites containing TC motif in HEK293T cells. Data are means ± SD (n = 3 independent experiments). Source data are provided with this paper.

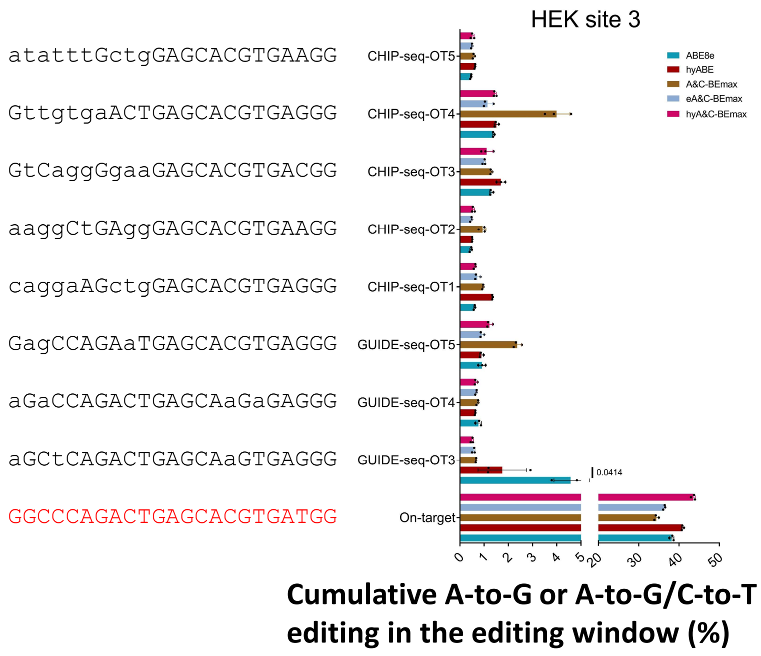

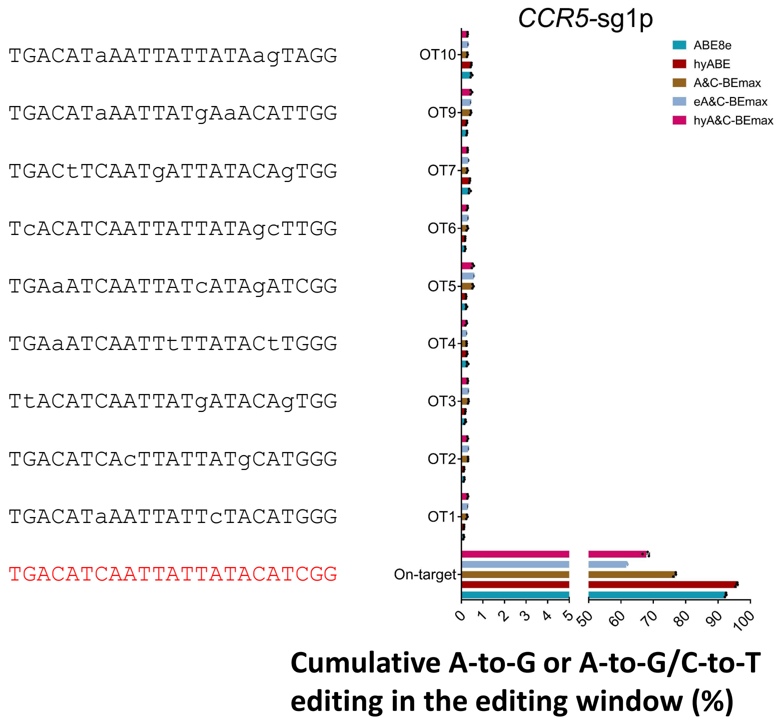

Supplementary Fig. 9 Cas9-dependent off-target assessments of hyABE and hyA&C-BEmax. Cas9-dependent DNA on and off-target analysis of the indicated targets (HEK site 3 and *CCR5*-sg1p) by ABE8e, hyABE, A&C-BEmax, eA&C-BEmax and hyA&C-BEmax in HEK293T cells. Lowercase protospacer sequences represent mismatched bases compared to their corresponding on-target sequences. All statistical testing was performed using two-tailed Student's t-test. Data are means ± SD (n = 3 independent experiments). Statistical source data are provided with the paper.

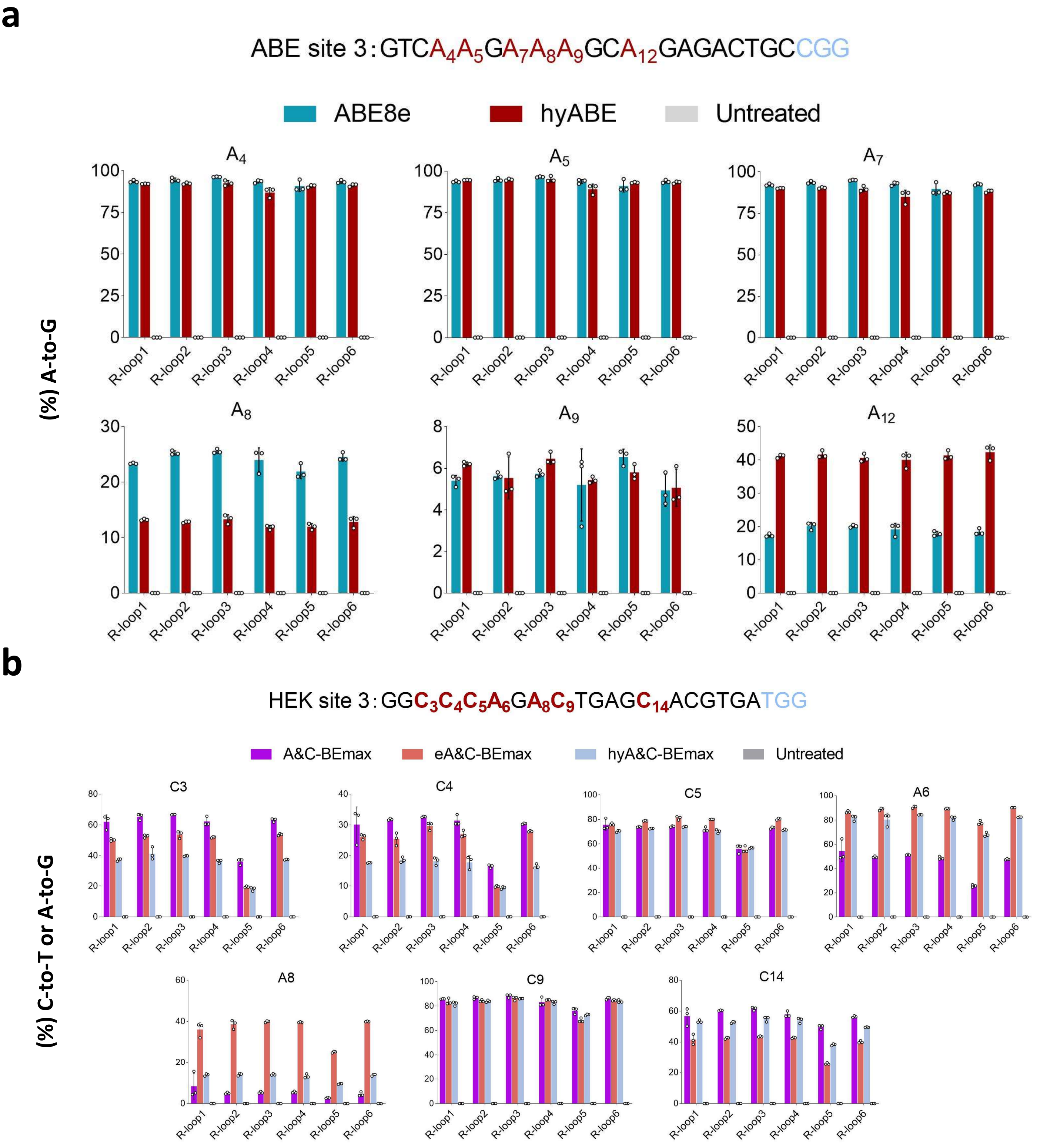

Supplementary Fig. 10 Cas9-independent off-target assessment in the R-loop assay. a, On-target base editing indued by ABE8e and hyABE using the modified orthogonal R-loop assay at each R-loop site with nSaCas9-sgRNA plasmid. Data are means ± SD (n = 3 independent experiments). **b,** On-target base editing indued by A&C-BEmax, eA&C-BEmax and hyA&C-BEmax using the modified orthogonal R-loop assay at each R-loop site with nSaCas9-sgRNA plasmid. Data are means ± SD (n = 3 independent experiments). Source data are provided with this paper.

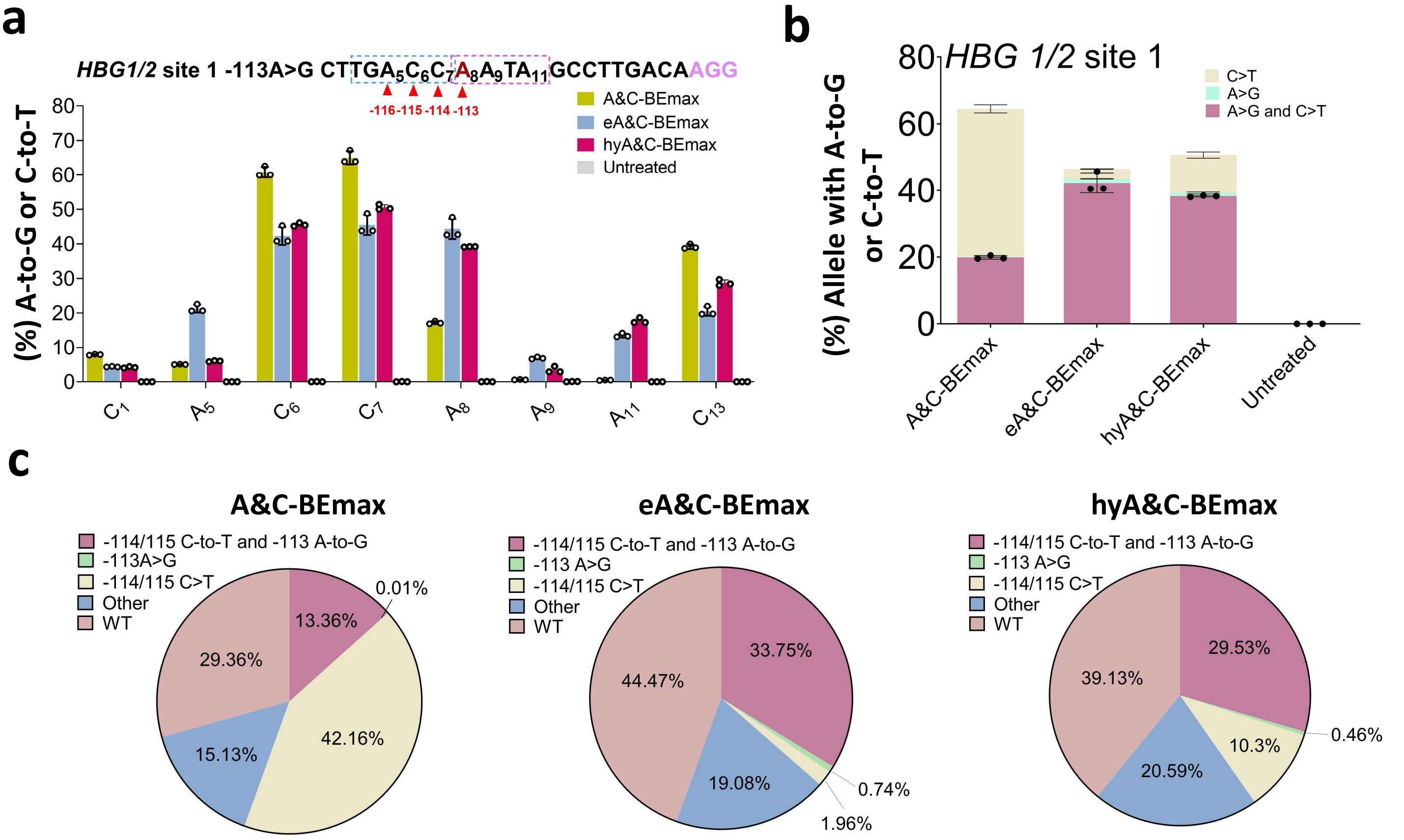

Supplementary Fig. 11 Efficient editing of *HBG* promoter by A&C-BEs in HEK293T cells. a, Base editing efficiency at the *HBG1* and *HBG2* promoter site in HEK293T cells transfected with plasmids of A&C-BEs and sgRNAs. Data are means ± SD (n = 3 independent experiments). **b,** The allele with A-to-G or C-to-T efficiency of A&C-BEmax, eA&C-BEmax and hyA&C-BEmax in HEK293T cells. Data are means ± SD (n = 3 independent experiments). **c,** Allele ratio distribution of *BCL11A* binding site disruption (corresponding to -114 or -115 C-to-T) or *GATA1* site creation (corresponding to -113 A-to-G) in A&C-BEs treated in HEK293T cells. Data are means ± SD (n = 3 independent experiments). Source data are provided with this paper

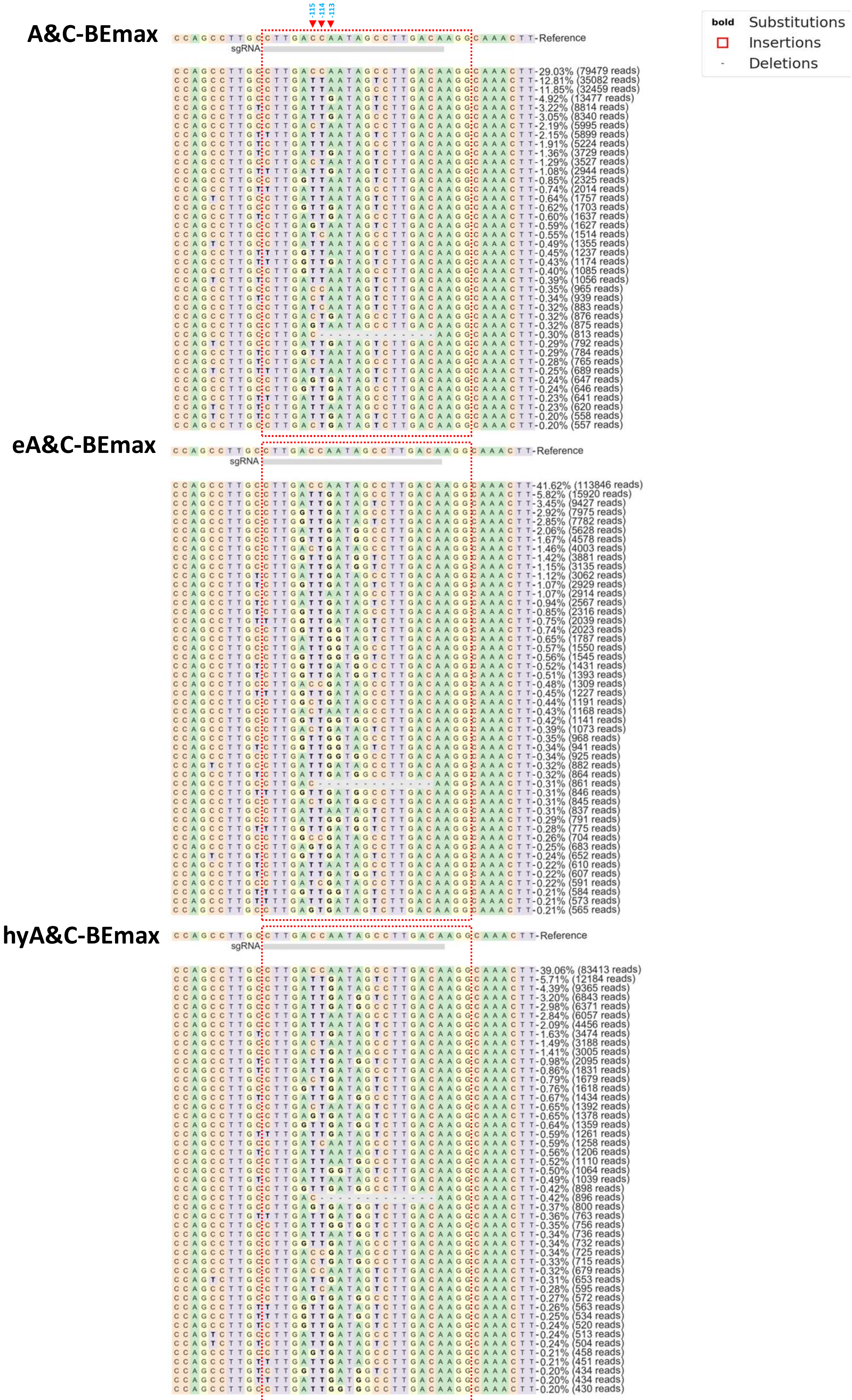

Supplementary Fig. 12 Allele table for *HBG 1/2* site 1 in HEK293T cells after A&C-BEmax, eA&C-BEmax and hyA&C-BEmax transfection. Target site allele is boxed in red dashed line. The percentile and sequencing reads of each allele at one representative of three independent experiments are listed on the right.

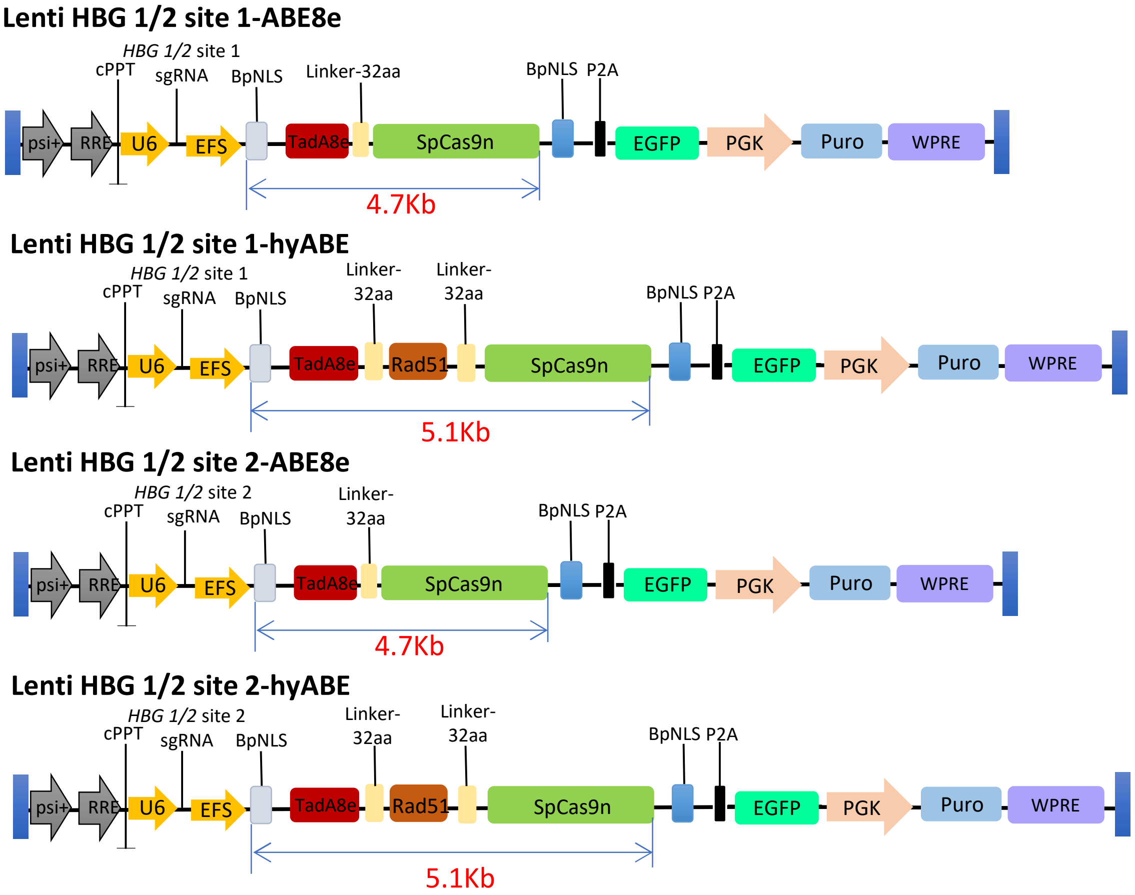

Supplementary Fig. 13 Schematic representation of lentivirus constructs for HUDEP-2 ($\Delta^{G}\gamma$) infection. Psi+, Psi packaging signal; RRE, Rev response element; cPPT, central polypurine tract; EFS, elongation factor 1a short promoter; BpNLS, bipartite nuclear localization signals; TadA8e, derived from evolved *E.coil* adenosine deaminase; spCas9n, Cas9 D10A; P2A, 2A self-cleaving peptide; Puro, puromycin selection marker; WPRE, post-transcriptional regulatory element; EF1α, elongation factor 1a promoter; EGFP, a maker for FACS; Linker length (in amino acids) are also shown.

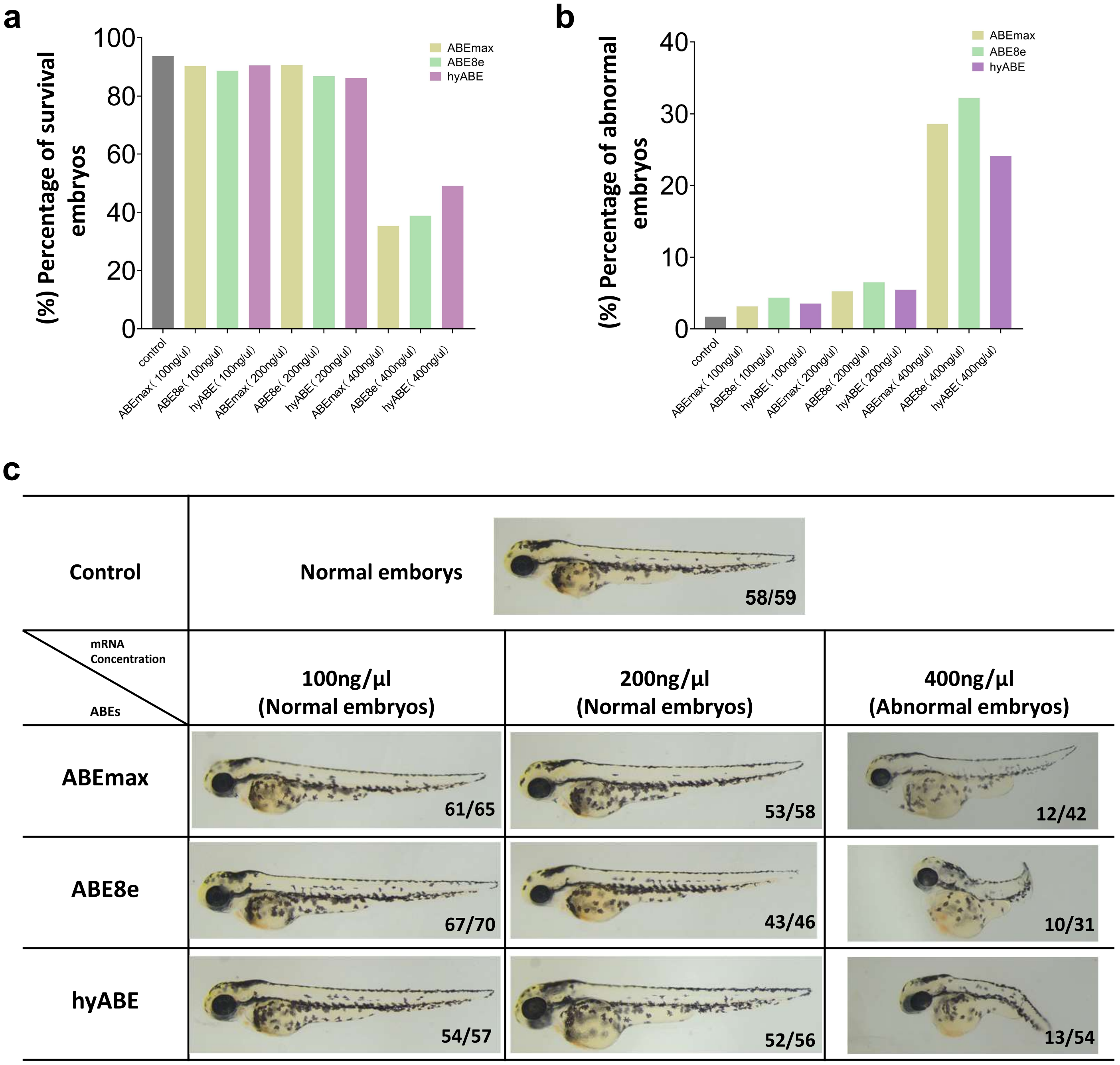

Supplementary Fig. 14 Percentages of survival and abnormal embryos in ABE-injected embryos. a, Survival rates of embryos injected with ABEmax, ABE8e or hyABE at a series of concentrations. **b**, Percentages of abnormal embryos injected with ABEmax, ABE8e or hyABE at a series of concentrations. **c,** Phenotypes of embryos injected with ABEmax, ABE8e or hyABE. The numbers in the bottom right corners indicate the number of embryos exhibiting the phenotype out of total injected embryos. Injection: 100ng/µl, 200ng/µl and 400ng/µl. Examination: 48 hpf. Source data are provided with this paper.

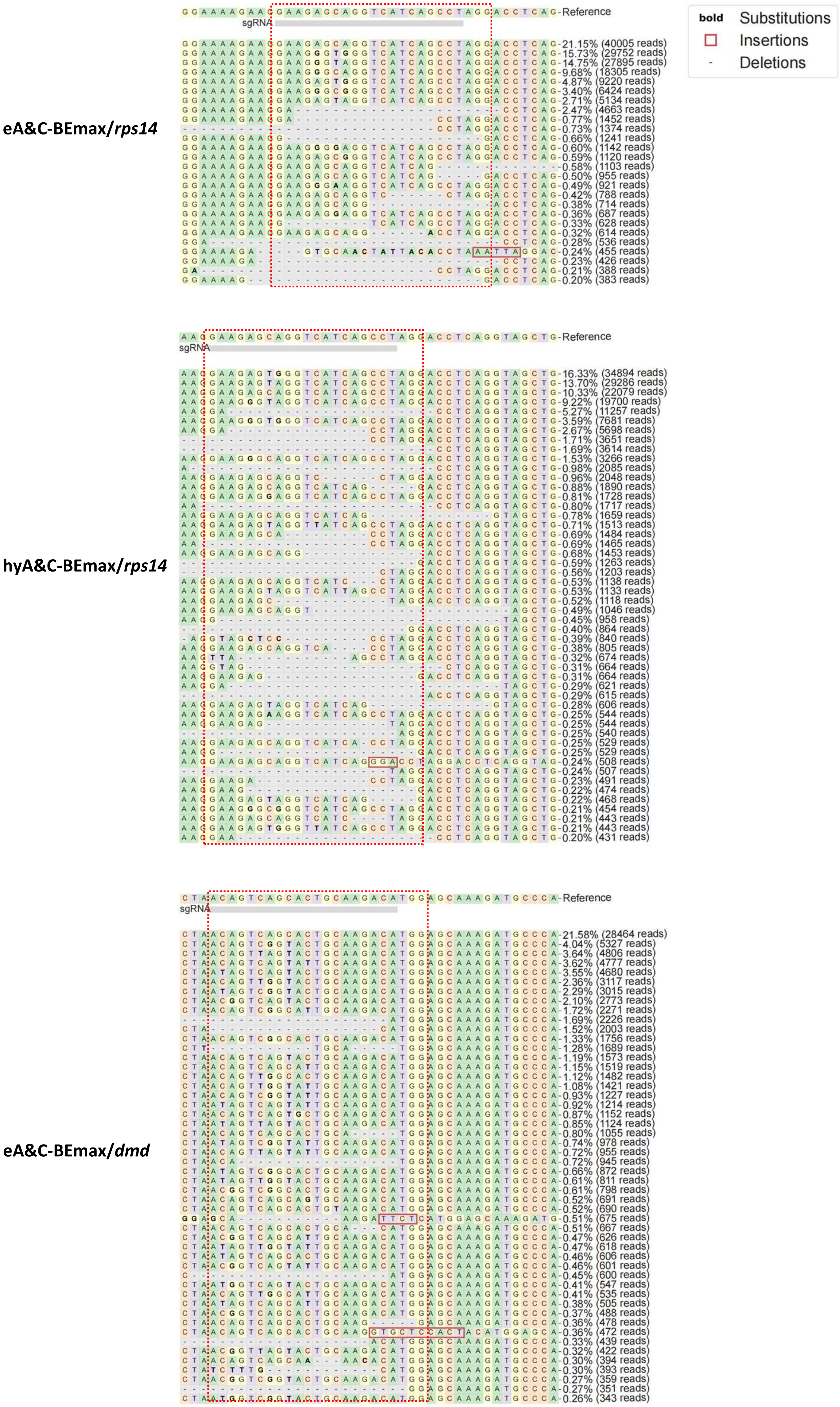

eA&C-BEmax/rps14
hyA&C-BEmax/rps14
eA&C-BEmax/dmd
bold Substitutions
Insertions
Deletions
Reference
sgRNA

hyA&C-BEmax/*dmd*

eA&C-BEmax/*egfra*

hyA&C-BEmax/*egfra*

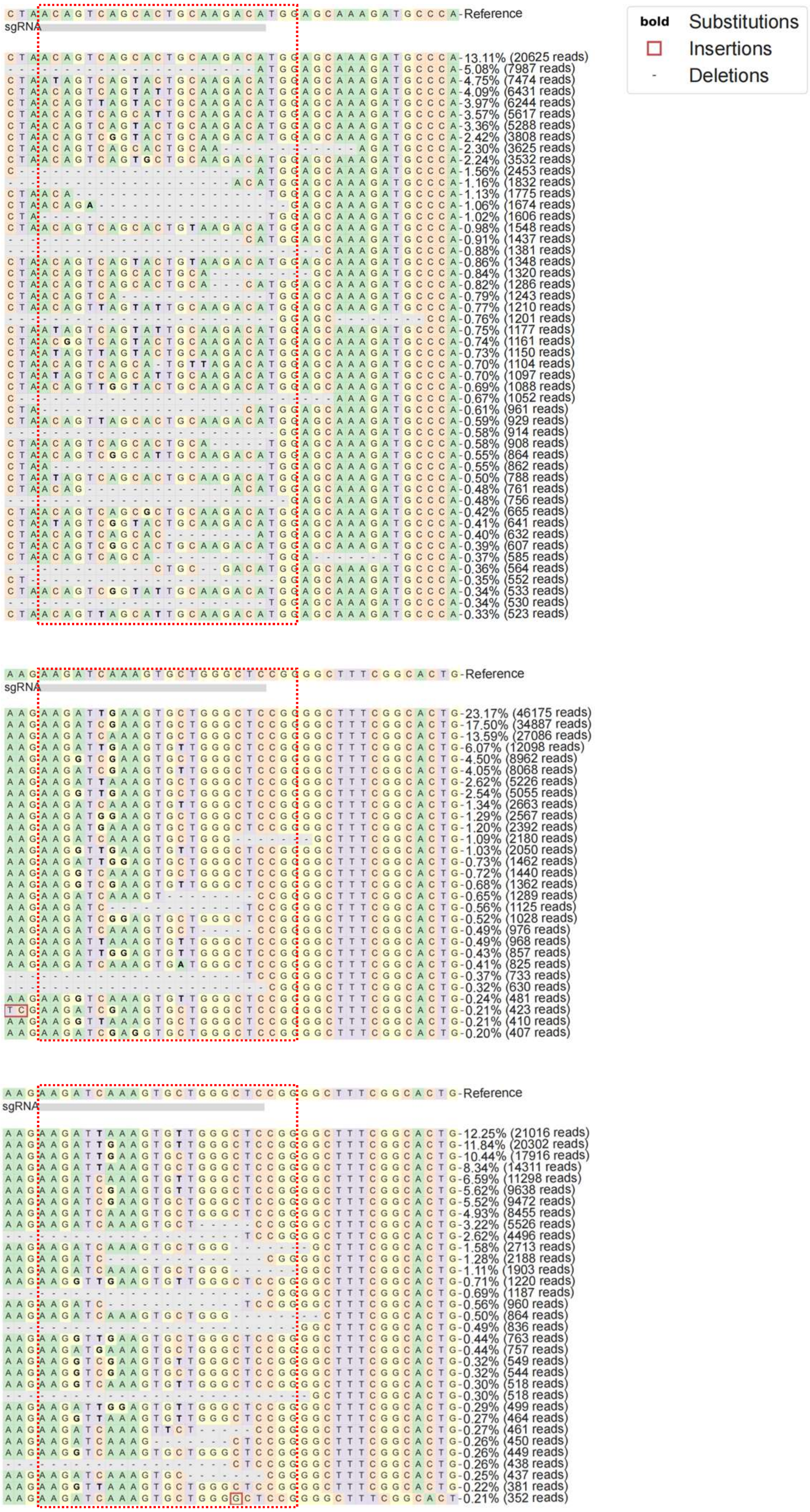

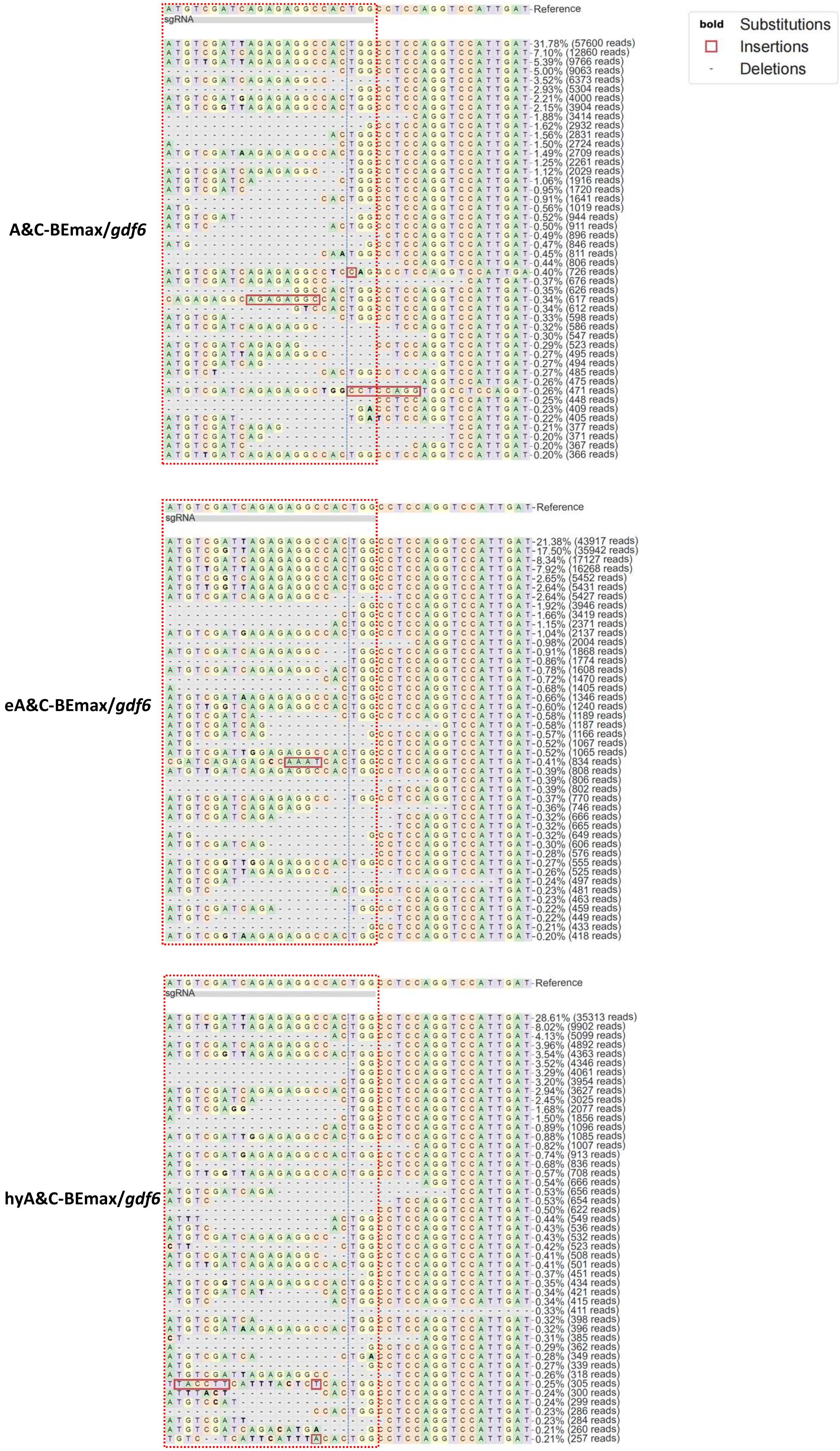
A&C-BEmax/gdf6
eA&C-BEmax/gdf6
hyA&C-BEmax/gdf6
Reference
sgRNA
bold Substitutions
Insertions
- Deletions

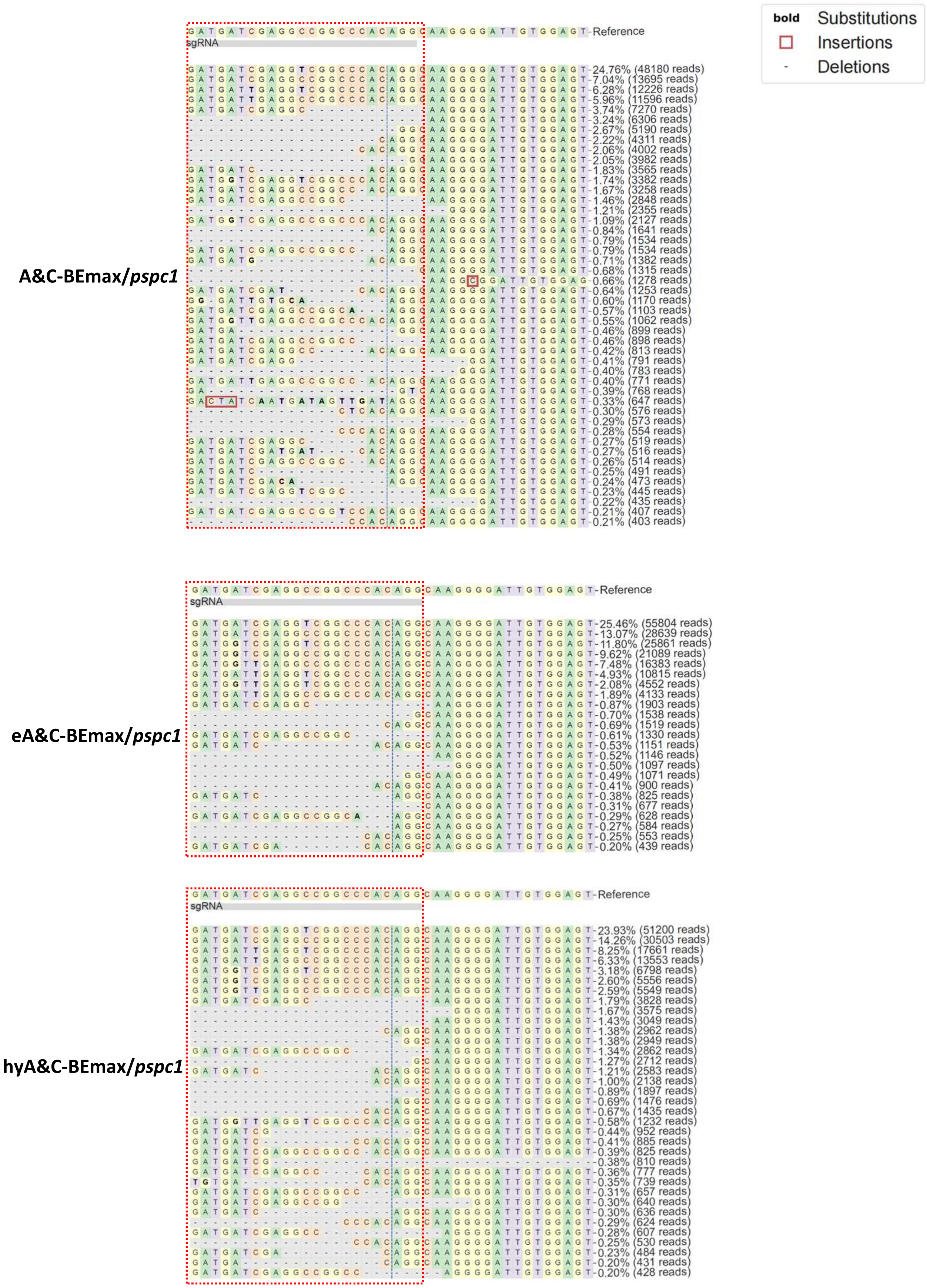

Supplementary Fig. 15 High-throughput sequencing indicates A-G and/or C-T substitutions and other allele types (insertion and deletion) for *rps14*, *dmd*, *egrfa*, *gdf6* and *pspc1* loci in zebrafish embryos injected with eA&C-BEmax, hyA&C-BEmax or A&C-BEmax. Target site allele is boxed in red dashed line.

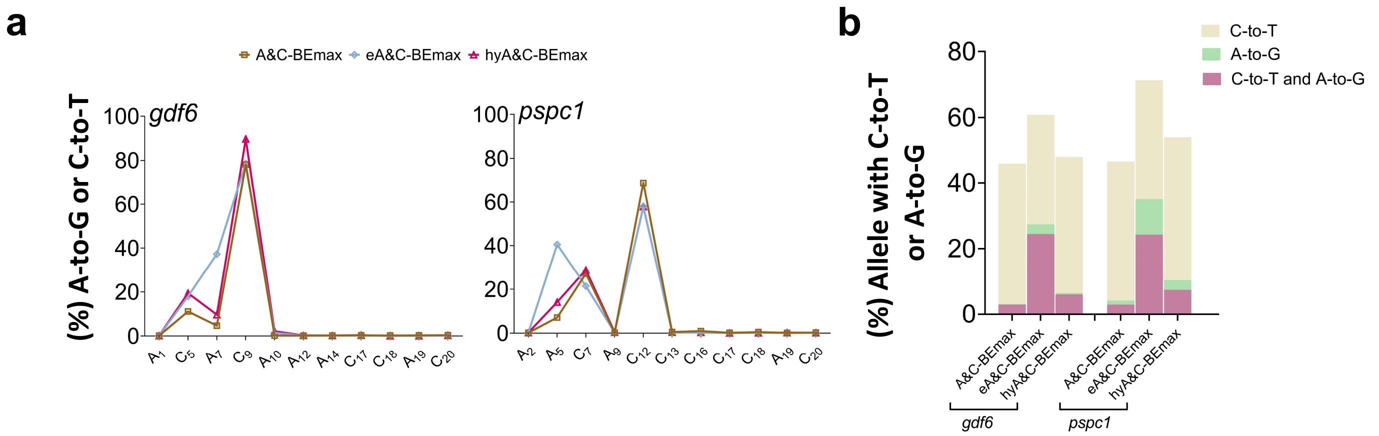

Supplementary Fig. 16 The editing efficiency of A&C-BEs in zebrafish embryos. a, The A-to-G or C-to-T editing efficiency of A&C-BEmax, eA&C-BEmax or hyA&C-BEmax were examined at *gdf6* and *pspc1* target sites in zebrafish embryo. **b,** The composition of A&C-BEmax, eA&C-BEmax and hyA&C-BEmax base editing products at the two loci. The individual data are shown as yellow (only C-to-T), green (only A-to-G) and plum purple (simultaneous C-to-T and A-to-G) columns. Source data are provided with this paper.

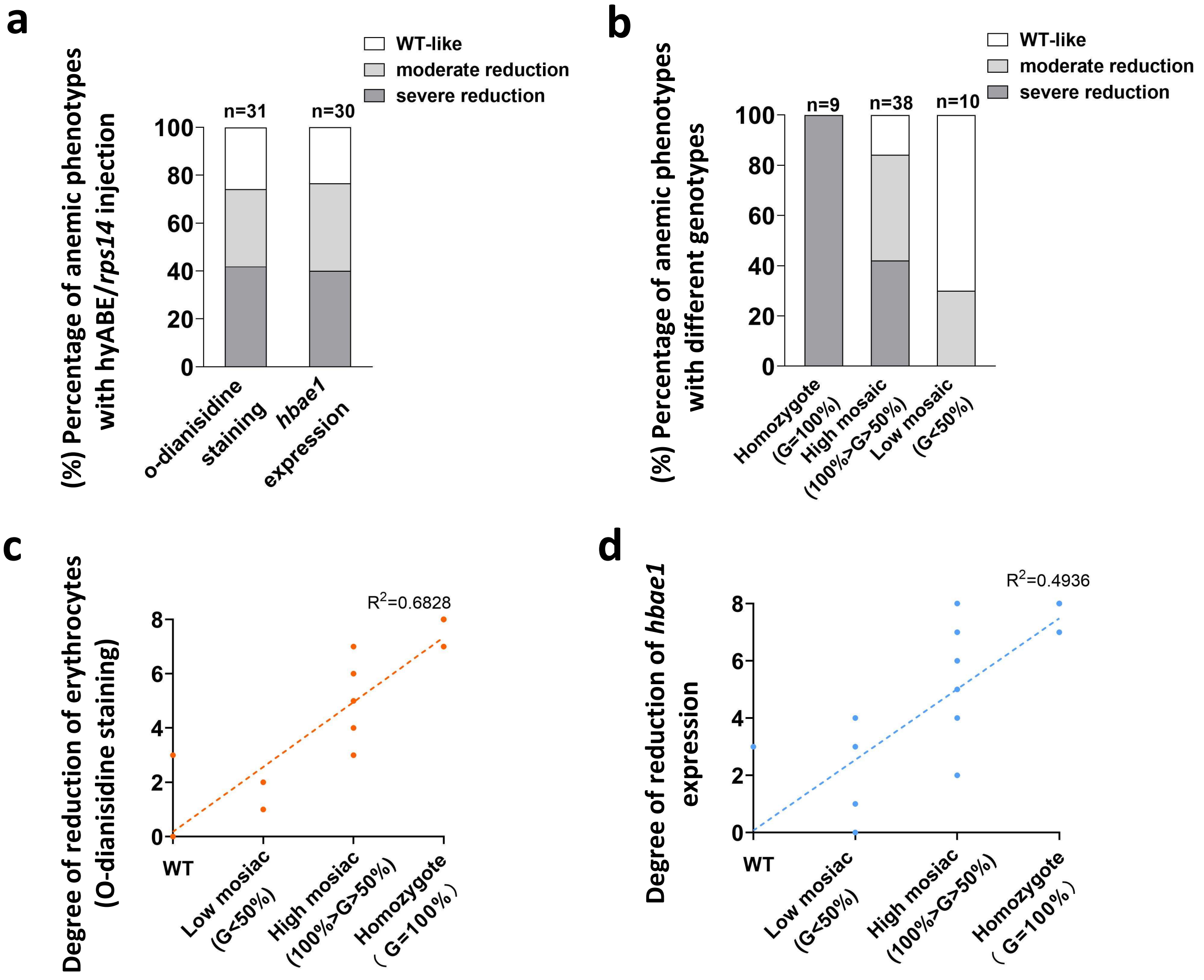

Supplementary Fig. 17 Morphological phenotypes and genotypes of embryos with hyABE/*rps14* injection. a, The stacked columns indicate the percentages of WT-like (white), moderate (light grey) and severe reduction (medium grey) of o-dianisidine or *hbae1* expression in zebrafish embryos injected with hyABE/*rps14* sgRNA. **b,** The stacked columns indicate the percentages of WT-like (white), moderate (light grey) and severe (medium grey) anemic phenotypes in zebrafish embryos with different genotypes. **c,** The linear relation between editing efficiency (X-axis) and the degree of the reduction of the erythrocytes (Y-axis). Number 0 to 8 represents different degrees of phenotype. **d,** The linear relation between editing efficiency (X-axis) and the degree of the reduction of *hbae1* expression (Y-axis). Number 0 to 8 represents different degrees of phenotype. Source data are provided with this paper.

Supplementary Note 1. FACS gating examples for GFP cell sorting conditions in adenine base editors treated group.

HEK293T Negative Control (Untreated)

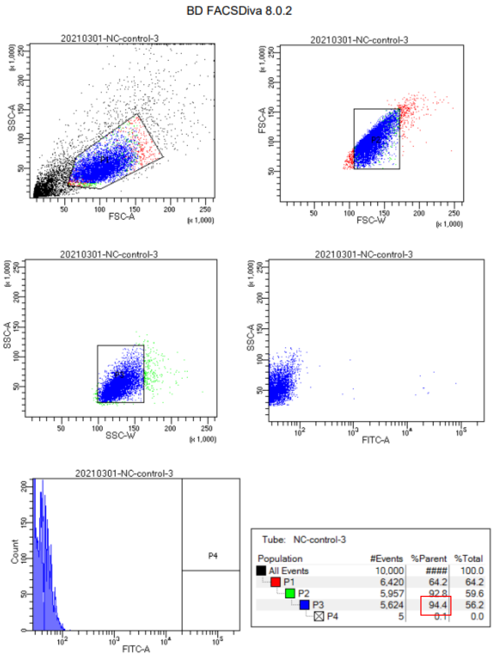

HEK293T Positive Control (Cas9n-P2A-GFP)

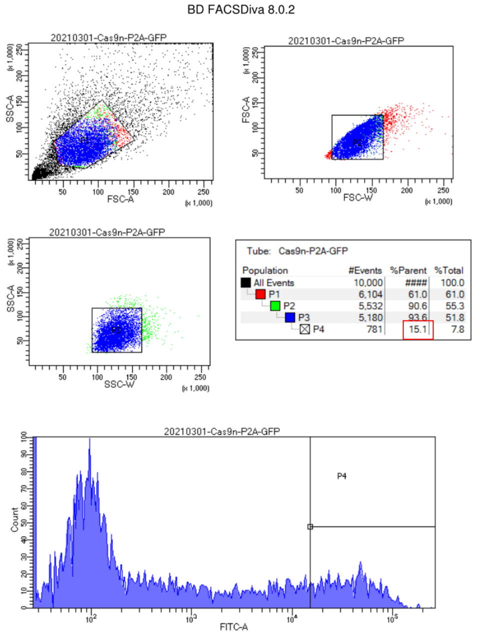

ABE8e-P2A-GFP

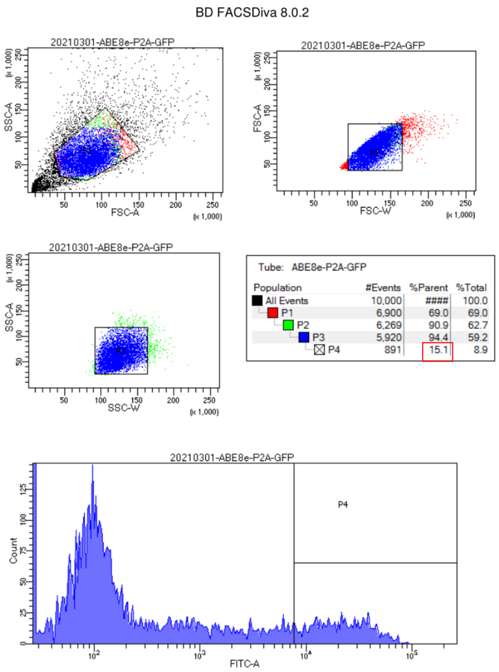

hyABE-P2A-GFP

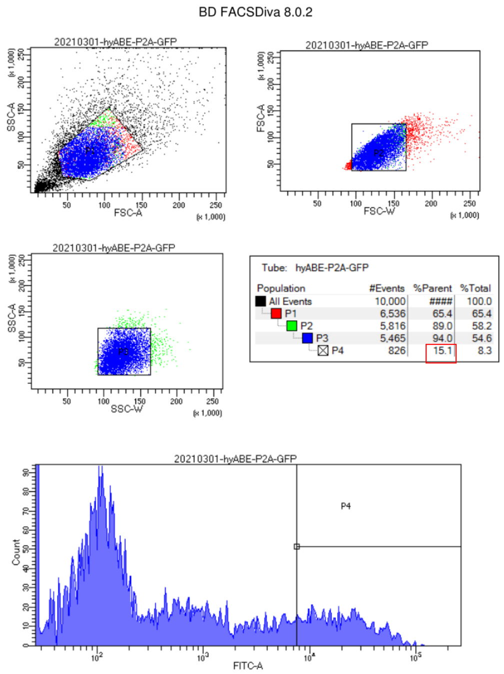

Supplementary Note 2. FACS gating examples for GFP cell sorting conditions in dual base editors treated group.

A&C-BEmax-P2A-GFP

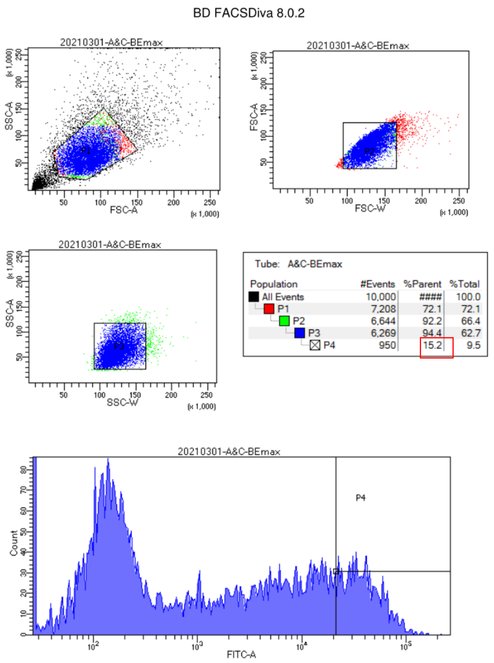

eA&C-BEmax-P2A-GFP

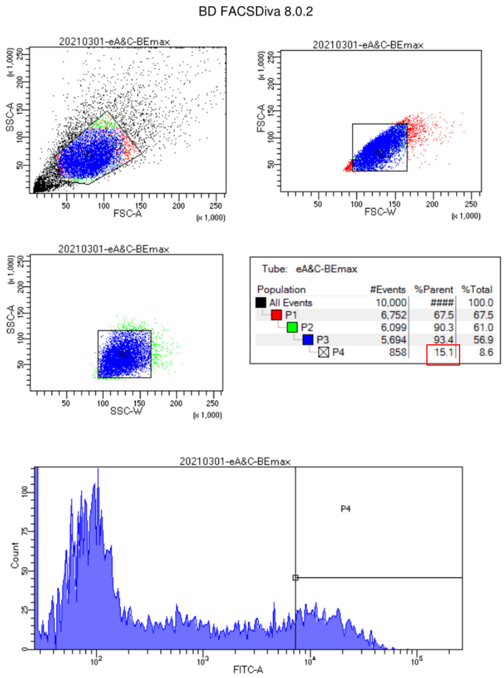

hyA&C-BEmax-P2A-GFP

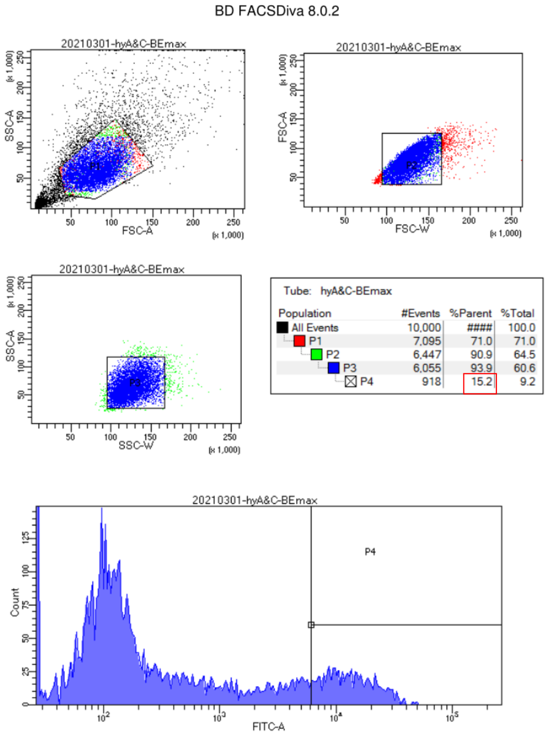

Supplementary Note 3. DNA sequences used for cell transfection in this study. With base editor sequences, bNLS sequences are in green, TadA, TadA* or TadA8e are in cyan, linker sequences are in yellow, Cas9 nickase sequences are in red, P2A/T2A sequences are in purple, GFP sequences are in dark green, Rad51DBD sequences are in brown, hAID sequences are in dark red, UGI sequences are in blue.

Linker-15aa: EAAAKEAAAKEAAAK
GAGGCCGCCGCCAAGGAAGCTGCCGCCAAGGAGGCCGCCGCCAAG
Linker-16aa: SGSETPGTSESATPES
AGCGGCAGCGAGACTCCCGGGACCTCAGAGTCCGCCACACCCGAAAGT
Linker-32aa: SGGSSGGSSGSETPGTSESATPESSGGSSGGS
TCCGGAGGATCTAGCGGAGGCTCCTCTGGCTCTGAGACACCTGGCACAAGCGAGAGCGCAACACCTGAAA
GCAGCGGGGGCAGCAGCGGGGGGTCA
Linker: SGGS
TCTGGCGGCTCA

ABEmax
ATGAAACGGACAGCCGACGGAAGCGAGTTCGAGTCACCAAAGAAGAAGCGGAAAGTCTCTGAAGTCGAG
TTTAGCCACGAGTATTGGATGAGGCACGCACTGACCCTGGCAAAGCGAGCATGGGATGAAAGAGAAGTCC
CCGTGGGCGCCGTGCTGGTGCACAACAATAGAGTGATCGGAGAGGGATGGAACAGGCCAATCGGCCGCCA
CGACCCTACCGCACACGCAGAGATCATGGCACTGAGGCAGGGAGGCCTGGTCATGCAGAATTACCGCCTGA
TCGATGCCACCCTGTATGTGACACTGGAGCCATGCGTGATGTGCGCAGGAGCAATGATCCACAGCAGGATC
GGAAGAGTGGTGTTCGGAGCACGGGACGCCAAGACCGGCGCAGCAGGCTCCCTGATGGATGTGCTGCAC
CACCCCGGCATGAACCACCGGGTGGAGATCACAGAGGGAATCCTGGCAGACGAGTGCGCCGCCCTGCTGA
GCGATTTCTTTAGAATGCGGAGACAGGAGATCAAGGCCCAGAAGAAGGCACAGAGCTCCACCGACTCTGG
AGGATCTAGCGGAGGATCCTCTGGAAGCGAGACACCAGGCACAAGCGAGTCCGCCACACCAGAGAGCTCC
GGCGGCTCCTCCGGAGGATCCTCTGAGGTGGAGTTTTCCCACGAGTACTGGATGAGACATGCCCTGACCCT
GGCCAAGAGGGCACGCGATGAGAGGGAGGTGCCTGTGGGAGCCGTGCTGGTGCTGAACAATAGAGTGAT
CGGCGAGGGCTGGAACAGAGCCATCGGCCTGCACGACCCAACAGCCCATGCCGAAATTATGGCCCTGAGA
CAGGGCGGCCTGGTCATGCAGAACTACAGACTGATTGACGCCACCCTGTACGTGACATTCGAGCCTTGCGT
GATGTGCGCCGGCGCCATGATCCACTCTAGGATCGGCCGCGTGGTGTTTGGCGTGAGGAACGCAAAAACC
GGCGCCGCAGGCTCCCTGATGGACGTGCTGCACTACCCCGGCATGAATCACCGCGTCGAAATTACCGAGGG
AATCCTGGCAGATGAATGTGCCGCCCTGCTGTGCTATTTCTTTCGGATGCCTAGACAGGTGTTCAATGCTCAG
AAGAAGGCCCAGAGCTCCACCGACTCCGGAGGATCTAGCGGAGGCTCCTCTGGCTCTGAGACACCTGGCA
CAAGCGAGAGCGCAACACCTGAAAGCAGCGGGGGCAGCAGCGGGGGGTCAGACAAGAAGTACAGCATC
GGCCTGGCCATCGGCACCAACTCTGTGGGCTGGGCCGTGATCACCGACGAGTACAAGGTGCCCAGCAAGA
AATTCAAGGTGCTGGGCAACACCGACCGGCACAGCATCAAGAAGAACCTGATCGGAGCCCTGCTGTTCGA
CAGCGGCGAAACAGCCGAGGCCACCCGGCTGAAGAGAACCGCCAGAAGAAGATACACCAGACGGAAGA
ACCGGATCTGCTATCTGCAAGAGATCTTCAGCAACGAGATGGCCAAGGTGGACGACAGCTTCTTCCACAGA
CTGGAAGAGTCCTTCCTGGTGGAAGAGGATAAGAAGCACGAGCGGCACCCCATCTTCGGCAACATCGTGG
ACGAGGTGGCCTACCACGAGAAGTACCCCACCATCTACCACCTGAGAAAGAAACTGGTGGACAGCACCGA
CAAGGCCGACCTGCGGCTGATCTATCTGGCCCTGGCCCACATGATCAAGTTCCGGGGCCACTTCCTGATCGA
GGGCGACCTGAACCCCGACAACAGCGACGTGGACAAGCTGTTCATCCAGCTGGTGCAGACCTACAACCAG
CTGTTCGAGGAAAACCCCATCAACGCCAGCGGCGTGGACGCCAAGGCCATCCTGTCTGCCAGACTGAGCA

AGAGCAGACGGCTGGAAAATCTGATCGCCCAGCTGCCCGGCGAGAAGAAGAATGGCCTGTTCGGAAACCT
GATTGCCCTGAGCCTGGGCCTGACCCCCAACTTCAAGAGCAACTTCGACCTGGCCGAGGATGCCAAACTGC
AGCTGAGCAAGGACACCTACGACGACGACCTGGACAACCTGCTGGCCCAGATCGGCGACCAGTACGCCGA
CCTGTTTCTGGCCGCCAAGAACCTGTCCGACGCCATCCTGCTGAGCGACATCCTGAGAGTGAACACCGAGA
TCACCAAGGCCCCCCTGAGCGCCTCTATGATCAAGAGATACGACGAGCACCACCAGGACCTGACCCTGCTG
AAAGCTCTCGTGCGGCAGCAGCTGCCTGAGAAGTACAAAGAGATTTTCTTCGACCAGAGCAAGAACGGCT
ACGCCGGCTACATTGACGGCGGAGCCAGCCAGGAAGAGTTCTACAAGTTCATCAAGCCCATCCTGGAAAA
GATGGACGGCACCGAGGAACTGCTCGTGAAGCTGAACAGAGAGGACCTGCTGCGGAAGCAGCGGACCTT
CGACAACGGCAGCATCCCCCACCAGATCCACCTGGGAGAGCTGCACGCCATTCTGCGGCGGCAGGAAGAT
TTTTACCCATTCCTGAAGGACAACCGGGAAAAGATCGAGAAGATCCTGACCTTCCGCATCCCCTACTACGTG
GGCCCTCTGGCCAGGGGAAACAGCAGATTCGCCTGGATGACCAGAAAGAGCGAGGAAACCATCACCCCCT
GGAACTTCGAGGAAGTGGTGGACAAGGGCGCTTCCGCCCAGAGCTTCATCGAGCGGATGACCAACTTCGA
TAAGAACCTGCCCAACGAGAAGGTGCTGCCCAAGCACAGCCTGCTGTACGAGTACTTCACCGTGTATAACG
AGCTGACCAAAGTGAAATACGTGACCGAGGGAATGAGAAAGCCCGCCTTCCTGAGCGGCGAGCAGAAAA
AGGCCATCGTGGACCTGCTGTTCAAGACCAACCGGAAAGTGACCGTGAAGCAGCTGAAAGAGGACTACTT
CAAGAAAATCGAGTGCTTCGACTCCGTGGAAATCTCCGGCGTGGAAGATCGGTTCAACGCCTCCCTGGGCA
CATACCACGATCTGCTGAAAATTATCAAGGACAAGGACTTCCTGGACAATGAGGAAAACGAGGACATTCTG
GAAGATATCGTGCTGACCCTGACACTGTTTGAGGACAGAGAGATGATCGAGGAACGGCTGAAAACCTATGC
CCACCTGTTCGACGACAAAGTGATGAAGCAGCTGAAGCGGCGGAGATACACCGGCTGGGGCAGGCTGAG
CCGGAAGCTGATCAACGGCATCCGGGACAAGCAGTCCGGCAAGACAATCCTGGATTTCCTGAAGTCCGAC
GGCTTCGCCAACAGAAACTTCATGCAGCTGATCCACGACGACAGCCTGACCTTTAAAGAGGACATCCAGAA
AGCCCAGGTGTCCGGCCAGGGCGATAGCCTGCACGAGCACATTGCCAATCTGGCCGGCAGCCCCGCCATTA
AGAAGGGCATCCTGCAGACAGTGAAGGTGGTGGACGAGCTCGTGAAAGTGATGGGCCGGCACAAGCCCG
AGAACATCGTGATCGAAATGGCCAGAGAGAACCAGACCACCCAGAAGGGACAGAAGAACAGCCGCGAGA
GAATGAAGCGGATCGAAGAGGGCATCAAAGAGCTGGGCAGCCAGATCCTGAAAGAACACCCCGTGGAAA
ACACCCAGCTGCAGAACGAGAAGCTGTACCTGTACTACCTGCAGAATGGGCGGGATATGTACGTGGACCAG
GAACTGGACATCAACCGGCTGTCCGACTACGATGTGGACCATATCGTGCCTCAGAGCTTTCTGAAGGACGAC
TCCATCGACAACAAGGTGCTGACCAGAAGCGACAAGAACCGGGGCAAGAGCGACAACGTGCCCTCCGAA
GAGGTCGTGAAGAAGATGAAGAACTACTGGCGGCAGCTGCTGAACGCCAAGCTGATTACCCAGAGAAAGT
TCGACAATCTGACCAAGGCCGAGAGAGGCGGCCTGAGCGAACTGGATAAGGCCGGCTTCATCAAGAGACA
GCTGGTGGAAACCCGGCAGATCACAAAGCACGTGGCACAGATCCTGGACTCCCGGATGAACACTAAGTAC
GACGAGAATGACAAGCTGATCCGGGAAGTGAAAGTGATCACCCTGAAGTCCAAGCTGGTGTCCGATTTCCG
GAAGGATTTCCAGTTTTACAAAGTGCGCGAGATCAACAACTACCACCACGCCCACGACGCCTACCTGAACG
CCGTCGTGGGAACCGCCCTGATCAAAAAGTACCCTAAGCTGGAAAGCGAGTTCGTGTACGGCGACTACAAG
GTGTACGACGTGCGGAAGATGATCGCCAAGAGCGAGCAGGAAATCGGCAAGGCTACCGCCAAGTACTTCT
TCTACAGCAACATCATGAACTTTTTCAAGACCGAGATTACCCTGGCCAACGGCGAGATCCGGAAGCGGCCT
CTGATCGAGACAAACGGCGAAACCGGGGAGATCGTGTGGGATAAGGGCCGGGATTTTGCCACCGTGCGG
AAAGTGCTGAGCATGCCCCAAGTGAATATCGTGAAAAAGACCGAGGTGCAGACAGGCGGCTTCAGCAAAG
AGTCTATCCTGCCCAAGAGGAACAGCGATAAGCTGATCGCCAGAAAGAAGGACTGGGACCCTAAGAAGTA
CGGCGGCTTCGACAGCCCCACCGTGGCCTATTCTGTGCTGGTGGTGGCCAAAGTGGAAAAGGGCAAGTCC
AAGAAACTGAAGAGTGTGAAAGAGCTGCTGGGGATCACCATCATGGAAAGAAGCAGCTTCGAGAAGAATC
CCATCGACTTTCTGGAAGCCAAGGGCTACAAAGAAGTGAAAAAGGACCTGATCATCAAGCTGCCTAAGTAC
TCCCTGTTCGAGCTGGAAAACGGCCGGAAGAGAATGCTGGCCTCTGCCGGCGAACTGCAGAAGGGAAAC
GAACTGGCCCTGCCCTCCAAATATGTGAACTTCCTGTACCTGGCCAGCCACTATGAGAAGCTGAAGGGCTCC

CCCGAGGATAATGAGCAGAAACAGCTGTTTGTGGAACAGCACAAGCACTACCTGGACGAGATCATCGAGCA
GATCAGCGAGTTCTCCAAGAGAGTGATCCTGGCCGACGCTAATCTGGACAAAGTGCTGTCCGCCTACAACA
AGCACCGGGATAAGCCCATCAGAGAGCAGGCCGAGAATATCATCCACCTGTTTACCCTGACCAATCTGGGA
GCCCCTGCCGCCTTCAAGTACTTTGACACCACCATCGACCGGAAGAGGTACACCAGCACCAAAGAGGTGCT
GGACGCCACCCTGATCCACCAGAGCATCACCGGCCTGTACGAGACACGGATCGACCTGTCTCAGCTGGGAG
GTGACTCTGGCGGCTCAAAAAGAACCGCCGACGGCAGCGAATTCGAGCCCAAGAAGAAGAGGAAAGTCG
GAAGCGGAGCTACTAACTTCAGCCTGCTGAAGCAGGCTGGAGACGTGGAGGAGAACCCTGGACCTATGGT
GAGCAAGGGCGAGGAGCTGTTCACCGGGGTGGTGCCCATCCTGGTCGAGCTGGACGGCGACGTAAACGG
CCACAAGTTCAGCGTGTCCGGCGAGGGCGAGGGCGATGCCACCTACGGCAAGCTGACCCTGAAGTTCATC
TGCACCACCGGCAAGCTGCCCGTGCCCTGGCCCACCCTCGTGACCACCCTGACCTATGGAGTGCAGTGCTTC
AGCCGCTACCCCGACCACATGAAGCAGCACGACTTCTTCAAGTCCGCCATGCCCGAAGGCTACGTCCAGGA
GCGCACCATCTTCTTCAAGGACGACGGCAACTACAAGACCCGCGCCGAGGTGAAGTTCGAGGGCGACACC
CTGGTGAACCGCATCGAGCTGAAGGGCATCGACTTCAAGGAGGACGGCAACATCCTGGGGCACAAGCTGG
AGTACAACTACAACAGCCACAACGTCTATATCATGGCCGACAAGCAGAAGAACGGCATCAAGGTGAACTTC
AAGATCCGCCACAACATCGAGGACGGCAGCGTGCAGCTCGCCGACCACTACCAGCAGAACACCCCCATCG
GCGACGGCCCCGTGCTGCTGCCCGACAACCACTACCTGAGCACCCAGTCCGCCCTGAGCAAAGACCCCAAC
GAGAAGCGCGATCACATGGTCCTGCTGGAGTTCGTGACCGCCGCCGGGATCACTCTCGGCATGGACGAGCT
GTACAAG

ABEmax-N-Rad51DBD

ATGAAACGGACAGCCGACGGAAGCGAGTTCGAGTCACCAAAGAAGAAGCGGAAAGTCATGGCAATGCAG
ATGCAGCTTGAAGCAAATGCAGATACTTCAGTGGAAGAAGAAAGCTTTGGCCCACAACCCATTTCACGGTT
AGAGCAGTGTGGCATAAATGCCAACGATGTGAAGAAATTGGAAGAAGCTGGATTCCATACTGTGGAGGCTG
TTGCCTATGCGCCAAAGAAGGAGCTAATAAATATTAAGGGAATTAGTGAAGCCAAAGCTGATAAAATTCTGG
CTGAGGCAGCTAAATTAGTTCCAATGGGTTTCACCACTGCAACTGAATTCCACCAAAGGCGGTCAGAGATCA
TACAGATTACTACTGGCTCCAAAGAGCTTGACAAACTACTTCAAAGCGGCAGCGAGACTCCCGGGACCTCA
GAGTCCGCCACACCCGAAAGTTCTGAAGTCGAGTTTAGCCACGAGTATTGGATGAGGCACGCACTGACCCT
GGCAAAGCGAGCATGGGATGAAAGAGAAGTCCCCGTGGGCGCCGTGCTGGTGCACAACAATAGAGTGATC
GGAGAGGGATGGAACAGGCCAATCGGCCGCCACGACCCTACCGCACACGCAGAGATCATGGCACTGAGGC
AGGGAGGCCTGGTCATGCAGAATTACCGCCTGATCGATGCCACCCTGTATGTGACACTGGAGCCATGCGTGA
TGTGCGCAGGAGCAATGATCCACAGCAGGATCGGAAGAGTGGTGTTCGGAGCACGGGACGCCAAGACCG
GCGCAGCAGGCTCCCTGATGGATGTGCTGCACCACCCCGGCATGAACCACCGGGTGGAGATCACAGAGGG
AATCCTGGCAGACGAGTGCGCCGCCCTGCTGAGCGATTTCTTTAGAATGCGGAGACAGGAGATCAAGGCCC
AGAAGAAGGCACAGAGCTCCACCGACTCTGGAGGATCTAGCGGAGGATCCTCTGGAAGCGAGACACCAG
GCACAAGCGAGTCCGCCACACCAGAGAGCTCCGGCGGCTCCTCCGGAGGATCCTCTGAGGTGGAGTTTTC
CCACGAGTACTGGATGAGACATGCCCTGACCCTGGCCAAGAGGGCACGCGATGAGAGGGAGGTGCCTGTG
GGAGCCGTGCTGGTGCTGAACAATAGAGTGATCGGCGAGGGCTGGAACAGAGCCATCGGCCTGCACGACC
CAACAGCCCATGCCGAAATTATGGCCCTGAGACAGGGCGGCCTGGTCATGCAGAACTACAGACTGATTGAC
GCCACCCTGTACGTGACATTCGAGCCTTGCGTGATGTGCGCCGGCGCCATGATCCACTCTAGGATCGGCCGC
GTGGTGTTTGGCGTGAGGAACGCAAAAACCGGCGCCGCAGGCTCCCTGATGGACGTGCTGCACTACCCCG
GCATGAATCACCGCGTCGAAATTACCGAGGGAATCCTGGCAGATGAATGTGCCGCCCTGCTGTGCTATTTCT
TTCGGATGCCTAGACAGGTGTTCAATGCTCAGAAGAAGGCCCAGAGCTCCACCGACTCCGGAGGATCTAGC
GGAGGCTCCTCTGGCTCTGAGACACCTGGCACAAGCGAGAGCGCAACACCTGAAAGCAGCGGGGGCAGC
AGCGGGGGGTCA--**Cas9n(Same as Cas9n in ABEmax)**--TCTGGCGGCTCAAAAAGAACCGCCGACG

GCAGCGAATTCGAGCCCAAGAAGAAGAGGAAAGTCGGAAGCGGAGCTACTAACTTCAGCCTGCTGAAGC
AGGCTGGAGACGTGGAGGAGAACCCTGGACCT--GFP**(Same as GFP in ABEmax)**

ABEmax-M-Rad51DBD

ATGAAACGGACAGCCGACGGAAGCGAGTTCGAGTCACCAAAGAAGAAGCGGAAAGTCTCTGAAGTCGAG
TTTAGCCACGAGTATTGGATGAGGCACGCACTGACCCTGGCAAAGCGAGCATGGGATGAAAGAGAAGTCC
CCGTGGGCGCCGTGCTGGTGCACAACAATAGAGTGATCGGAGAGGGATGGAACAGGCCAATCGGCCGCCA
CGACCCTACCGCACACGCAGAGATCATGGCACTGAGGCAGGGAGGCCTGGTCATGCAGAATTACCGCCTGA
TCGATGCCACCCTGTATGTGACACTGGAGCCATGCGTGATGTGCGCAGGAGCAATGATCCACAGCAGGATC
GGAAGAGTGGTGTTCGGAGCACGGGACGCCAAGACCGGCGCAGCAGGCTCCCTGATGGATGTGCTGCAC
CACCCCGGCATGAACCACCGGGTGGAGATCACAGAGGGAATCCTGGCAGACGAGTGCGCCGCCCTGCTGA
GCGATTTCTTTAGAATGCGGAGACAGGAGATCAAGGCCCAGAAGAAGGCACAGAGCTCCACCGACTCTGG
AGGATCTAGCGGAGGATCCTCTGGAAGCGAGACACCAGGCACAAGCGAGTCCGCCACACCAGAGAGCTCC
GGCGGCTCCTCCGGAGGATCCTCTGAGGTGGAGTTTTCCCACGAGTACTGGATGAGACATGCCCTGACCCT
GGCCAAGAGGGCACGCGATGAGAGGGAGGTGCCTGTGGGAGCCGTGCTGGTGCTGAACAATAGAGTGAT
CGGCGAGGGCTGGAACAGAGCCATCGGCCTGCACGACCCAACAGCCCATGCCGAAATTATGGCCCTGAGA
CAGGGCGGCCTGGTCATGCAGAACTACAGACTGATTGACGCCACCCTGTACGTGACATTCGAGCCTTGCGT
GATGTGCGCCGGCGCCATGATCCACTCTAGGATCGGCCGCGTGGTGTTTGGCGTGAGGAACGCAAAAACC
GGCGCCGCAGGCTCCCTGATGGACGTGCTGCACTACCCCGGCATGAATCACCGCGTCGAAATTACCGAGGG
AATCCTGGCAGATGAATGTGCCGCCCTGCTGTGCTATTTCTTTCGGATGCCTAGACAGGTGTTCAATGCTCAG
AAGAAGGCCCAGAGCTCCACCGACAGCGGAGGATCTAGCGGAGGATCAAGCGGAAGCGAGACTCCTGGA
ACCAGCGAAAGCGCAACCCCAGAAAGCAGCGGAGGAAGTAGCGGAGGAAGCATGGCAATGCAGATGCAG
CTTGAAGCAAATGCAGATACTTCAGTGGAAGAAGAAAGCTTTGGCCCACAACCCATTTCACGGTTAGAGCA
GTGTGGCATAAATGCCAACGATGTGAAGAAATTGGAAGAAGCTGGATTCCATACTGTGGAGGCTGTTGCCT
ATGCGCCAAAGAAGGAGCTAATAAATATTAAGGGAATTAGTGAAGCCAAAGCTGATAAAATTCTGGCTGAG
GCAGCTAAATTAGTTCCAATGGGTTTCACCACTGCAACTGAATTCCACCAAAGGCGGTCAGAGATCATACAG
ATTACTACTGGCTCCAAAGAGCTTGACAAACTACTTCAATCCGGAGGATCTAGCGGAGGCTCCTCTGGCTCT
GAGACACCTGGCACAAGCGAGAGCGCAACACCTGAAAGCAGCGGGGGCAGCAGCGGGGGGTCA--**Cas9
n(Same as Cas9n in ABEmax)**--TCTGGCGGCTCAAAAAGAACCGCCGACGGCAGCGAATTCGAGCCCA
AGAAGAAGAGGAAAGTCGGAAGCGGAGCTACTAACTTCAGCCTGCTGAAGCAGGCTGGAGACGTGGAGG
AGAACCCTGGACCT--GFP**(Same as GFP in ABEmax)**

ABEmax-C-Rad51DBD

ATGAAACGGACAGCCGACGGAAGCGAGTTCGAGTCACCAAAGAAGAAGCGGAAAGTCTCTGAAGTCGAG
TTTAGCCACGAGTATTGGATGAGGCACGCACTGACCCTGGCAAAGCGAGCATGGGATGAAAGAGAAGTCC
CCGTGGGCGCCGTGCTGGTGCACAACAATAGAGTGATCGGAGAGGGATGGAACAGGCCAATCGGCCGCCA
CGACCCTACCGCACACGCAGAGATCATGGCACTGAGGCAGGGAGGCCTGGTCATGCAGAATTACCGCCTGA
TCGATGCCACCCTGTATGTGACACTGGAGCCATGCGTGATGTGCGCAGGAGCAATGATCCACAGCAGGATC
GGAAGAGTGGTGTTCGGAGCACGGGACGCCAAGACCGGCGCAGCAGGCTCCCTGATGGATGTGCTGCAC
CACCCCGGCATGAACCACCGGGTGGAGATCACAGAGGGAATCCTGGCAGACGAGTGCGCCGCCCTGCTGA
GCGATTTCTTTAGAATGCGGAGACAGGAGATCAAGGCCCAGAAGAAGGCACAGAGCTCCACCGACTCTGG
AGGATCTAGCGGAGGATCCTCTGGAAGCGAGACACCAGGCACAAGCGAGTCCGCCACACCAGAGAGCTCC
GGCGGCTCCTCCGGAGGATCCTCTGAGGTGGAGTTTTCCCACGAGTACTGGATGAGACATGCCCTGACCCT
GGCCAAGAGGGCACGCGATGAGAGGGAGGTGCCTGTGGGAGCCGTGCTGGTGCTGAACAATAGAGTGAT

CGGCGAGGGCTGGAACAGAGCCATCGGCCTGCACGACCCAACAGCCCATGCCGAAATTATGGCCCTGAGA
CAGGGCGGCCTGGTCATGCAGAACTACAGACTGATTGACGCCACCCTGTACGTGACATTCGAGCCTTGCGT
GATGTGCGCCGGCGCCATGATCCACTCTAGGATCGGCCGCGTGGTGTTTGGCGTGAGGAACGCAAAAACC
GGCGCCGCAGGCTCCCTGATGGACGTGCTGCACTACCCCGGCATGAATCACCGCGTCGAAATTACCGAGGG
AATCCTGGCAGATGAATGTGCCGCCCTGCTGTGCTATTTCTTTCGGATGCCTAGACAGGTGTTCAATGCTCAG
AAGAAGGCCCAGAGCTCCACCGACTCCGGAGGATCTAGCGGAGGCTCCTCTGGCTCTGAGACACCTGGCA
CAAGCGAGAGCGCAACACCTGAAAGCAGCGGGGGCAGCAGCGGGGGGTCA--**Cas9n**(**Same as Cas9n
in ABEmax**)--TCTGGTGGTTCTGCAATGCAGATGCAGCTTGAAGCAAATGCAGATACTTCAGTGGAAGAAG
AAAGCTTTGGCCCACAACCCATTTCACGGTTAGAGCAGTGTGGCATAAATGCCAACGATGTGAAGAAATTG
GAAGAAGCTGGATTCCATACTGTGGAGGCTGTTGCCTATGCGCCAAAGAAGGAGCTAATAAATATTAAGGG
AATTAGTGAAGCCAAAGCTGATAAAATTCTGGCTGAGGCAGCTAAATTAGTTCCAATGGGTTTCACCACTGC
AACTGAATTCCACCAAAGGCGGTCAGAGATCATACAGATTACTACTGGCTCCAAAGAGCTTGACAAACTACT
TCAATCTGGCGGCTCAAAAAGAACCGCCGACGGCAGCGAATTCGAGCCCAAGAAGAAGAGGAAAGTCGG
AAGCGGAGCTACTAACTTCAGCCTGCTGAAGCAGGCTGGAGACGTGGAGGAGAACCCTGGACCT--**GFP**(**Same as GFP in ABEmax**)

ABE8e

ATGAAACGGACAGCCGACGGAAGCGAGTTCGAGTCACCAAAGAAGAAGCGGAAAGTCTCTGAGGTGGAG
TTTTCCCACGAGTACTGGATGAGACATGCCCTGACCCTGGCCAAGAGGGCACGGGATGAGAGGGAGGTGC
CTGTGGGAGCCGTGCTGGTGCTGAACAATAGAGTGATCGGCGAGGGCTGGAACAGAGCCATCGGCCTGCA
CGACCCAACAGCCCATGCCGAAATTATGGCCCTGAGACAGGGCGGCCTGGTCATGCAGAACTACAGACTGA
TTGACGCCACCCTGTACGTGACATTCGAGCCTTGCGTGATGTGCGCCGGCGCCATGATCCACTCTAGGATCG
GCCGCGTGGTGTTTGGCGTGAGGAACTCAAAAAGAGGCGCCGCAGGCTCCCTGATGAACGTGCTGAACTA
CCCCGGCATGAATCACCGCGTCGAAATTACCGAGGGAATCCTGGCAGATGAATGTGCCGCCCTGCTGTGCG
ATTTCTATCGGATGCCTAGACAGGTGTTCAATGCTCAGAAGAAGGCCCAGAGCTCCATCAACTCCGGAGGAT
CTAGCGGAGGCTCCTCTGGCTCTGAGACACCTGGCACAAGCGAGAGCGCAACACCTGAAAGCAGCGGGG
GCAGCAGCGGGGGGTCA--**Cas9n**(**Same as Cas9n in ABEmax**)--TCTGGCGGCTCAAAAAGAACCGC
CGACGGCAGCGAATTCGAGCCCAAGAAGAAGAGGAAAGTCGGAAGCGGAGCTACTAACTTCAGCCTGCT
GAAGCAGGCTGGAGACGTGGAGGAGAACCCTGGACCT--**GFP**(**Same as GFP in ABEmax**)

ABE8e-N-Rad51DBD

ATGAAACGGACAGCCGACGGAAGCGAGTTCGAGTCACCAAAGAAGAAGCGGAAAGTCGCAATGCAGATG
CAGCTTGAAGCAAATGCAGATACTTCAGTGGAAGAAGAAAGCTTTGGCCCACAACCCATTTCACGGTTAGA
GCAGTGTGGCATAAATGCCAACGATGTGAAGAAATTGGAAGAAGCTGGATTCCATACTGTGGAGGCTGTTG
CCTATGCGCCAAAGAAGGAGCTAATAAATATTAAGGGAATTAGTGAAGCCAAAGCTGATAAAATTCTGGCTG
AGGCAGCTAAATTAGTTCCAATGGGTTTCACCACTGCAACTGAATTCCACCAAAGGCGGTCAGAGATCATAC
AGATTACTACTGGCTCCAAAGAGCTTGACAAACTACTTCAAAGCGGCAGCGAGACTCCCGGGACCTCAGAG
TCCGCCACACCCGAAAGTTCTGAGGTGGAGTTTTCCCACGAGTACTGGATGAGACATGCCCTGACCCTGGC
CAAGAGGGCACGGGATGAGAGGGAGGTGCCTGTGGGAGCCGTGCTGGTGCTGAACAATAGAGTGATCGG
CGAGGGCTGGAACAGAGCCATCGGCCTGCACGACCCAACAGCCCATGCCGAAATTATGGCCCTGAGACAG
GGCGGCCTGGTCATGCAGAACTACAGACTGATTGACGCCACCCTGTACGTGACATTCGAGCCTTGCGTGATG
TGCGCCGGCGCCATGATCCACTCTAGGATCGGCCGCGTGGTGTTTGGCGTGAGGAACTCAAAAAGAGGCG
CCGCAGGCTCCCTGATGAACGTGCTGAACTACCCCGGCATGAATCACCGCGTCGAAATTACCGAGGGAATC
CTGGCAGATGAATGTGCCGCCCTGCTGTGCGATTTCTATCGGATGCCTAGACAGGTGTTCAATGCTCAGAAG

AAGGCCCAGAGCTCCATCAACTCCGGAGGATCTAGCGGAGGCTCCTCTGGCTCTGAGACACCTGGCACAAG
CGAGAGCGCAACACCTGAAAGCAGCGGGGGCAGCAGCGGGGGGTCA--**Cas9n(Same as Cas9n in A
BEmax)**--TCTGGCGGCTCAAAAAGAACCGCCGACGGCAGCGAATTCGAGCCCAAGAAGAAGAGGAAAGT
CGGAAGCGGAGCTACTAACTTCAGCCTGCTGAAGCAGGCTGGAGACGTGGAGGAGAACCCTGGACCT--**GF
P(Same as GFP in ABEmax)**

ABE8e-M-Rad51DBD (hyABE)

ATGAAACGGACAGCCGACGGAAGCGAGTTCGAGTCACCAAAGAAGAAGCGGAAAGTCTCTGAGGTGGAG
TTTTCCCACGAGTACTGGATGAGACATGCCCTGACCCTGGCCAAGAGGGCACGGGATGAGAGGGAGGTGC
CTGTGGGAGCCGTGCTGGTGCTGAACAATAGAGTGATCGGCGAGGGCTGGAACAGAGCCATCGGCCTGCA
CGACCCAACAGCCCATGCCGAAATTATGGCCCTGAGACAGGGCGGCCTGGTCATGCAGAACTACAGACTGA
TTGACGCCACCCTGTACGTGACATTCGAGCCTTGCGTGATGTGCGCCGGCGCCATGATCCACTCTAGGATCG
GCCGCGTGGTGTTTGGCGTGAGGAACTCAAAAAGAGGCGCCGCAGGCTCCCTGATGAACGTGCTGAACTA
CCCCGGCATGAATCACCGCGTCGAAATTACCGAGGGAATCCTGGCAGATGAATGTGCCGCCCTGCTGTGCG
ATTTCTATCGGATGCCTAGACAGGTGTTCAATGCTCAGAAGAAGGCCCAGAGCTCCATCAACTCCGGAGGAT
CTAGCGGAGGCTCCTCTGGCTCTGAGACACCTGGCACAAGCGAGAGCGCAACACCTGAAAGCAGCGGGG
GCAGCAGCGGGGGGTCAGCAATGCAGATGCAGCTTGAAGCAAATGCAGATACTTCAGTGGAAGAAGAAA
GCTTTGGCCCACAACCCATTTCACGGTTAGAGCAGTGTGGCATAAATGCCAACGATGTGAAGAAATTGGAA
GAAGCTGGATTCCATACTGTGGAGGCTGTTGCCTATGCGCCAAAGAAGGAGCTAATAAATATTAAGGGAATT
AGTGAAGCCAAAGCTGATAAAATTCTGGCTGAGGCAGCTAAATTAGTTCCAATGGGTTTCACCACTGCAACT
GAATTCCACCAAAGGCGGTCAGAGATCATACAGATTACTACTGGCTCCAAAGAGCTTGACAAACTACTTCAA
TCCGGAGGATCTAGCGGAGGCTCCTCTGGCTCTGAGACACCTGGCACAAGCGAGAGCGCAACACCTGAAA
GCAGCGGGGGCAGCAGCGGGGGGTCA--**Cas9n(Same as Cas9n in ABEmax)**--TCTGGCGGCTCAAA
AAGAACCGCCGACGGCAGCGAATTCGAGCCCAAGAAGAAGAGGAAAGTCGGAAGCGGAGCTACTAACTT
CAGCCTGCTGAAGCAGGCTGGAGACGTGGAGGAGAACCCTGGACCT--**GFP(Same as GFP in ABEma
x)**

ABE8e-C-Rad51DBD

ATGAAACGGACAGCCGACGGAAGCGAGTTCGAGTCACCAAAGAAGAAGCGGAAAGTCTCTGAGGTGGAG
TTTTCCCACGAGTACTGGATGAGACATGCCCTGACCCTGGCCAAGAGGGCACGGGATGAGAGGGAGGTGC
CTGTGGGAGCCGTGCTGGTGCTGAACAATAGAGTGATCGGCGAGGGCTGGAACAGAGCCATCGGCCTGCA
CGACCCAACAGCCCATGCCGAAATTATGGCCCTGAGACAGGGCGGCCTGGTCATGCAGAACTACAGACTGA
TTGACGCCACCCTGTACGTGACATTCGAGCCTTGCGTGATGTGCGCCGGCGCCATGATCCACTCTAGGATCG
GCCGCGTGGTGTTTGGCGTGAGGAACTCAAAAAGAGGCGCCGCAGGCTCCCTGATGAACGTGCTGAACTA
CCCCGGCATGAATCACCGCGTCGAAATTACCGAGGGAATCCTGGCAGATGAATGTGCCGCCCTGCTGTGCG
ATTTCTATCGGATGCCTAGACAGGTGTTCAATGCTCAGAAGAAGGCCCAGAGCTCCATCAACTCCGGAGGAT
CTAGCGGAGGCTCCTCTGGCTCTGAGACACCTGGCACAAGCGAGAGCGCAACACCTGAAAGCAGCGGGG
GCAGCAGCGGGGGGTCA--**Cas9n(Same as Cas9n in ABEmax)**--TCTGGTGGTTCTGCAATGCAGATG
CAGCTTGAAGCAAATGCAGATACTTCAGTGGAAGAAGAAAGCTTTGGCCCACAACCCATTTCACGGTTAGA
GCAGTGTGGCATAAATGCCAACGATGTGAAGAAATTGGAAGAAGCTGGATTCCATACTGTGGAGGCTGTTG
CCTATGCGCCAAAGAAGGAGCTAATAAATATTAAGGGAATTAGTGAAGCCAAAGCTGATAAAATTCTGGCTG
AGGCAGCTAAATTAGTTCCAATGGGTTTCACCACTGCAACTGAATTCCACCAAAGGCGGTCAGAGATCATAC
AGATTACTACTGGCTCCAAAGAGCTTGACAAACTACTTCAATCTGGCGGCTCAAAAAGAACCGCCGACGGC
AGCGAATTCGAGCCCAAGAAGAAGAGGAAAGTCGGAAGCGGAGCTACTAACTTCAGCCTGCTGAAGCAG

GCTGGAGACGTGGAGGAGAACCCTGGACCT--**GFP(Same as GFP in ABEmax)**

hyAID-BE4max

ATGAAACGGACAGCCGACGGAAGCGAGTTCGAGTCACCAAAGAAGAAGCGGAAAGTCGACAGCCTGCTG
ATGAACCGGAGAAAGTTCCTGTATCAGTTTAAGAATGTGAGGTGGGCAAAGGGCAGGCGCGAGACCTACC
TGTGCTATGTGGTGAAGCGGAGAGATAGCGCCACATCTTTCAGCCTGGACTTTGGCTACCTGCGGAACAAG
AATGGCTGCCACGTGGAGCTGCTGTTTCTGAGATACATCTCCGACTGGGATCTGGACCCAGGCAGGTGTTAT
CGCGTGACCTGGTTCACATCCTGGTCTCCCTGCTACGATTGTGCCCGGCACGTGGCCGACTTTCTGAGAGGC
AACCCTAATCTGTCTCTGAGGATCTTCACCGCCCGCCTGTATTTTTGCGAGGATAGGAAGGCCGAGCCAGAG
GGACTGAGGCGCCTGCACAGGGCCGGCGTGCAGATCGCCATCATGACATTCAAGGACTACTTTTATTGTTG
GAACACCTTCGTGGAGAATCACGAGCGGACATTTAAGGCCTGGGAGGGACTGCACGAGAACTCCGTGCGG
CTGTCTAGACAGCTGCGGAGAATCCTGCTGCCATCCGGAGGATCTAGCGGAGGCTCCTCTGGCTCTGAGAC
ACCTGGCACAAGCGAGAGCGCAACACCTGAAAGCAGCGGGGGCAGCAGCGGGGGGTCAGCAATGCAGA
TGCAGCTTGAAGCAAATGCAGATACTTCAGTGGAAGAAGAAAGCTTTGGCCCACAACCCATTTCACGGTTA
GAGCAGTGTGGCATAAATGCCAACGATGTGAAGAAATTGGAAGAAGCTGGATTCCATACTGTGGAGGCTGT
TGCCTATGCGCCAAAGAAGGAGCTAATAAATATTAAGGGAATTAGTGAAGCCAAAGCTGATAAAATTCTGGC
TGAGGCAGCTAAATTAGTTCCAATGGGTTTCACCACTGCAACTGAATTCCACCAAAGGCGGTCAGAGATCAT
ACAGATTACTACTGGCTCCAAAGAGCTTGACAAACTACTTCAATCCGGAGGATCTAGCGGAGGCTCCTCTGG
CTCTGAGACACCTGGCACAAGCGAGAGCGCAACACCTGAAAGCAGCGGGGGCAGCAGCGGGGGGTCA--
Cas9n(Same as Cas9n in ABEmax)--TCTGGCGGCTCAAAAAGAACCGCCGACGGCAGCGAATTCGAG
CCCAAGAAGAAGAGGAAAGTCGGAAGCGGAGCTACTAACTTCAGCCTGCTGAAGCAGGCTGGAGACGTG
GAGGAGAACCCTGGACCTACTAATCTGAGCGACATCATTGAGAAGGAGACTGGGAAACAGCTGGTCATTCA
GGAGTCCATCCTGATGCTGCCTGAGGAGGTGGAGGAAGTGATCGGCAACAAGCCAGAGTCTGACATCCTG
GTGCACACCGCCTACGACGAGTCCACAGATGAGAATGTGATGCTGCTGACCTCTGACGCCCCCGAGTATAAG
CCTTGGGCCCTGGTCATCCAGGATTCTAACGGCGAGAATAAGATCAAGATGCTGAGCGGAGGATCCGGAGG
ATCTGGAGGCAGCACCAACCTGTCTGACATCATCGAGAAGGAGACAGGCAAGCAGCTGGTCATCCAGGAG
AGCATCCTGATGCTGCCCGAAGAAGTCGAAGAAGTGATCGGAAACAAGCCTGAGAGCGATATCCTGGTCCA
TACCGCCTACGACGAGAGTACCGACGAAAATGTGATGCTGCTGACATCCGACGCCCCAGAGTATAAGCCCTG
GGCTCTGGTCATCCAGGATTCCAACGGAGAGAACAAAATCAAAATGCTGTCTGGCGGCTCAAAAAGAACCG
CCGACGGCAGCGAATTCGAGCCCAAGAAGAAGAGGAAAGTCGGAAGCGGAGCTACTAACTTCAGCCTGCT
GAAGCAGGCTGGAGACGTGGAGGAGAACCCTGGACCT--**GFP(Same as GFP in ABEmax)**

A&C-BEmax

ATGAAGAGGACCGCCGATGGCTCTGAGTTCGAGAGCCCCAAGAAGAAGCGGAAGGTGGACAGCCTGCTG
ATGAACCGGAGAAAGTTCCTGTATCAGTTTAAGAATGTGAGGTGGGCAAAGGGCAGGCGCGAGACCTACC
TGTGCTATGTGGTGAAGCGGAGAGATAGCGCCACATCTTTCAGCCTGGACTTTGGCTACCTGCGGAACAAG
AATGGCTGCCACGTGGAGCTGCTGTTTCTGAGATACATCTCCGACTGGGATCTGGACCCAGGCAGGTGTTAT
CGCGTGACCTGGTTCACATCCTGGTCTCCCTGCTACGATTGTGCCCGGCACGTGGCCGACTTTCTGAGAGGC
AACCCTAATCTGTCTCTGAGGATCTTCACCGCCCGCCTGTATTTTTGCGAGGATAGGAAGGCCGAGCCAGAG
GGACTGAGGCGCCTGCACAGGGCCGGCGTGCAGATCGCCATCATGACATTCAAGGACTACTTTTATTGTTG
GAACACCTTCGTGGAGAATCACGAGCGGACATTTAAGGCCTGGGAGGGACTGCACGAGAACTCCGTGCGG
CTGTCTAGACAGCTGCGGAGAATCCTGCTGCCAGAGGCCGCCGCCAAGGAAGCTGCCGCCAAGGAGGCCG
CCGCCAAGAGCGAGGTGGAGTTCAGCCACGAGTACTGGATGAGACACGCCCTGACCCTGGCTAAGAGAGC
TTGGGATGAGAGAGAGGTGCCCGTGGGAGCTGTTCTGGTTCATAACAACAGGGTGATCGGCGAGGGATGG

AACAGACCTATCGGGAGACACGACCCAACCGCTCATGCTGAAATCATGGCCCTGAGACAAGGAGGGCTGG
TGATGCAAAATTACAGACTGATCGACGCAACCCTGTACGTGACCCTGGAGCCTTGTGTGATGTGCGCAGGA
GCAATGATCCACTCCAGAATCGGCAGAGTGGTGTTCGGAGCTAGAGATGCCAAAACCGGAGCCGCTGGAA
GCCTGATGGACGTTCTGCATCACCCCGGAATGAATCACAGAGTGGAGATAACCGAGGGCATTCTGGCCGAC
GAGTGTGCTGCTCTGCTGTCTGATTTCTTCAGAATGAGAAGGCAGGAAATCAAGGCCCAGAAAAAGGCCCA
AAGCAGCACCGACAGCGGAGGATCTAGCGGAGGATCAAGCGGAAGCGAGACTCCTGGAACCAGCGAAAG
CGCAACCCCAGAAAGCAGCGGAGGAAGTAGCGGAGGAAGCTCAGAAGTCGAGTTCAGCCATGAGTATTG
GATGAGACATGCTCTGACCCTGGCAAAGAGAGCAAGAGACGAGAGAGAGGTCCCAGTGGGAGCAGTTCT
GGTGCTGAACAACAGAGTGATCGGGGAGGGGTGGAACAGAGCAATCGGACTGCACGACCCTACAGCACA
CGCAGAAATAATGGCACTGAGACAAGGGGGGCTCGTGATGCAAAACTACAGGCTGATCGACGCCACCCTG
TACGTCACATTTGAGCCCTGTGTGATGTGTGCCGGAGCCATGATTCACAGTAGAATCGGCCGGGTGGTGTTC
GGTGTGAGAAACGCTAAAACAGGCGCCGCCGGAAGCCTGATGGATGTTCTGCATTACCCCGGCATGAATCA
CCGGGTGGAGATCACAGAGGGCATCCTGGCTGACGAATGTGCCGCTCTGCTGTGTTACTTCTTCAGAATGCC
CCGACAAGTGTTCAACGCCCAGAAGAAAGCCCAGTCAAGCACCGACTCTGGCGGATCTAGCGGTGGATCTA
GCGGCTCTGAGACCCCTGGAACATCCGAATCCGCCACTCCAGAGAGCAGCGGAGGCTCTTCTGGAGGATC
AGACAAGAAGTACAGCATCGGCCTGgccATCGGCACCAACTCTGTGGGCTGGGCCGTGATCACCGACGAGT
ACAAGGTGCCCAGCAAGAAATTCAAGGTGCTGGGCAACACCGACCGGCACAGCATCAAGAAGAACCTGAT
CGGAGCCCTGCTGTTCGACAGCGGCGAAACAGCCGAGGCCACCCGGCTGAAGAGAACCGCCAGAAGAAG
ATACACCAGACGGAAGAACCGGATCTGCTATCTGCAAGAGATCTTCAGCAACGAGATGGCCAAGGTGGACG
ACAGCTTCTTCCACAGACTGGAAGAGTCCTTCCTGGTGGAAGAGGATAAGAAGCACGAGCGGCACCCCAT
CTTCGGCAACATCGTGGACGAGGTGGCCTACCACGAGAAGTACCCCACCATCTACCACCTGAGAAAGAAAC
TGGTGGACAGCACCGACAAGGCCGACCTGCGGCTGATCTATCTGGCCCTGGCCCACATGATCAAGTTCCGG
GGCCACTTCCTGATCGAGGGCGACCTGAACCCCGACAACAGCGACGTGGACAAGCTGTTCATCCAGCTGGT
GCAGACCTACAACCAGCTGTTCGAGGAAAACCCCATCAACGCCAGCGGCGTGGACGCCAAGGCCATCCTG
TCTGCCAGACTGAGCAAGAGCAGACGGCTGGAAAATCTGATCGCCCAGCTGCCCGGCGAGAAGAAGAATG
GCCTGTTCGGAAACCTGATTGCCCTGAGCCTGGGCCTGACCCCCAACTTCAAGAGCAACTTCGACCTGGCC
GAGGATGCCAAACTGCAGCTGAGCAAGGACACCTACGACGACGACCTGGACAACCTGCTGGCCCAGATCG
GCGACCAGTACGCCGACCTGTTTCTGGCCGCCAAGAACCTGTCCGACGCCATCCTGCTGAGCGACATCCTG
AGAGTGAACACCGAGATCACCAAGGCCCCCCTGAGCGCCTCTATGATCAAGAGATACGACGAGCACCACCA
GGACCTGACCCTGCTGAAAGCTCTCGTGCGGCAGCAGCTGCCTGAGAAGTACAAAGAGATTTTCTTCGACC
AGAGCAAGAACGGCTACGCCGGCTACATTGACGGCGGAGCCAGCCAGGAAGAGTTCTACAAGTTCATCAA
GCCCATCCTGGAAAAGATGGACGGCACCGAGGAACTGCTCGTGAAGCTGAACAGAGAGGACCTGCTGCG
GAAGCAGCGGACCTTCGACAACGGCAGCATCCCCCACCAGATCCACCTGGGAGAGCTGCACGCCATTCTGC
GGCGGCAGGAAGATTTTTACCCATTCCTGAAGGACAACCGGGAAAAGATCGAGAAGATCCTGACCTTCCGC
ATCCCCTACTACGTGGGCCCTCTGGCCAGGGGAAACAGCAGATTCGCCTGGATGACCAGAAAGAGCGAGG
AAACCATCACCCCCTGGAACTTCGAGGAAGTGGTGGACAAGGGCGCTTCCGCCCAGAGCTTCATCGAGCG
GATGACCAACTTCGATAAGAACCTGCCCAACGAGAAGGTGCTGCCCAAGCACAGCCTGCTGTACGAGTACT
TCACCGTGTATAACGAGCTGACCAAAGTGAAATACGTGACCGAGGGAATGAGAAAGCCCGCCTTCCTGAGC
GGCGAGCAGAAAAAGGCCATCGTGGACCTGCTGTTCAAGACCAACCGGAAAGTGACCGTGAAGCAGCTG
AAAGAGGACTACTTCAAGAAAATCGAGTGCTTCGACTCCGTGGAAATCTCCGGCGTGGAAGATCGGTTCAA
CGCCTCCCTGGGCACATACCACGATCTGCTGAAAATTATCAAGGACAAGGACTTCCTGGACAATGAGGAAA
ACGAGGACATTCTGGAAGATATCGTGCTGACCCTGACACTGTTTGAGGACAGAGAGATGATCGAGGAACG
GCTGAAAACCTATGCCCACCTGTTCGACGACAAAGTGATGAAGCAGCTGAAGCGGCGGAGATACACCGGC
TGGGGCAGGCTGAGCCGGAAGCTGATCAACGGCATCCGGGACAAGCAGTCCGGCAAGACAATCCTGGATT

TCCTGAAGTCCGACGGCTTCGCCAACAGAAACTTCATGCAGCTGATCCACGACGACAGCCTGACCTTTAAA
GAGGACATCCAGAAAGCCCAGGTGTCCGGCCAGGGCGATAGCCTGCACGAGCACATTGCCAATCTGGCCG
GCAGCCCCGCCATTAAGAAGGGCATCCTGCAGACAGTGAAGGTGGTGGACGAGCTCGTGAAAGTGATGGG
CCGGCACAAGCCCGAGAACATCGTGATCGAAATGGCCAGAGAGAACCAGACCACCCAGAAGGGACAGAA
GAACAGCCGCGAGAGAATGAAGCGGATCGAAGAGGGCATCAAAGAGCTGGGCAGCCAGATCCTGAAAGA
ACACCCCGTGGAAAACACCCAGCTGCAGAACGAGAAGCTGTACCTGTACTACCTGCAGAATGGGCGGATA
TGTACGTGGACCAGGAACTGGACATCAACCGGCTGTCCGACTACGATGTGGACCATATCGTGCCTCAGAGCT
TTCTGAAGGACGACTCCATCGACAACAAGGTGCTGACCAGAAGCGACAAGAACCGGGGCAAGAGCGACA
ACGTGCCCTCCGAAGAGGTCGTGAAGAAGATGAAGAACTACTGGCGGCAGCTGCTGAACGCCAAGCTGAT
TACCCAGAGAAAGTTCGACAATCTGACCAAGGCCGAGAGAGGCGGCCTGAGCGAACTGGATAAGGCCGG
CTTCATCAAGAGACAGCTGGTGGAAACCCGGCAGATCACAAAGCACGTGGCACAGATCCTGGACTCCCGG
ATGAACACTAAGTACGACGAGAATGACAAGCTGATCCGGGAAGTGAAAGTGATCACCCTGAAGTCCAAGCT
GGTGTCCGATTTCCGGAAGGATTTCCAGTTTTACAAAGTGCGCGAGATCAACAACTACCACCACGCCCACG
ACGCCTACCTAAACGCCGTCGTGGGAACCGCCCTGATCAAAAAGTACCCTAAGCTGGAAAGCGAGTTCGTG
TACGGCGACTACAAGGTGTACGACGTGCGGAAGATGATCGCCAAGAGCGAGCAGGAAATCGGCAAGGCTA
CCGCCAAGTACTTCTTCTACAGCAACATCATGAACTTTTTCAAGACCGAGATTACCCTGGCCAACGGCGAGA
TCCGGAAGCGGCCTCTGATCGAGACAAACGGCGAAACCGGGGAGATCGTGTGGGATAAGGGCCGGGATT
TTGCCACCGTGCGGAAAGTGCTGAGCATGCCCCAAGTGAATATCGTGAAAAAGACCGAGGTGCAGACAGG
CGGCTTCAGCAAAGAGTCTATCCTGCCCAAGAGGAACAGCGATAAGCTGATCGCCAGAAAGAAGGACTGG
GACCCTAAGAAGTACGGCGGCTTCGACAGCCCCACCGTGGCCTATTCTGTGCTGGTGGTGGCCAAAGTGGA
AAAGGGCAAGTCCAAGAAACTGAAGAGTGTGAAAGAGCTGCTGGGGATCACCATCATGGAAAGAAGCAG
CTTCGAGAAGAATCCCATCGACTTTCTGGAAGCCAAGGGCTACAAAGAAGTGAAAAAGGACCTGATCATCA
AGCTGCCTAAGTACTCCCTGTTCGAGCTGGAAAACGGCCGGAAGAGAATGCTGGCCTCTGCCGGCGAACT
GCAGAAGGGAAACGAACTGGCCCTGCCCTCCAAATATGTGAACTTCCTGTACCTGGCCAGCCACTATGAGA
AGCTGAAGGGCTCCCCCGAGGATAATGAGCAGAAACAGCTGTTTGTGGAACAGCACAAGCACTACCTGGA
CGAGATCATCGAGCAGATCAGCGAGTTCTCCAAGAGAGTGATCCTGGCCGACGCTAATCTGGACAAAGTGC
TGTCCGCCTACAACAAGCACCGGGATAAGCCCATCAGAGAGCAGGCCGAGAATATCATCCACCTGTTTACCC
TGACCAATCTGGGAGCCCCTGCCGCCTTCAAGTACTTTGACACCACCATCGACCGGAAGAGGTACACCAGC
ACCAAAGAGGTGCTGGACGCCACCCTGATCCACCAGAGCATCACCGGCCTGTACGAGACACGGATCGACCT
GTCTCAGCTGGGAGGTGACAAAAGGCCGGCGGCCACGAAAAAGGCCGGCCAGGCAAAAAAGAAAAAGG
GATCCGGAGAGGGCAGAGGAAGTCTGCTAACATGCGGTGACGTCGAGGAGAATCCTGGCCCAACTAATCT
GAGCGACATCATTGAGAAGGAGACTGGGAAACAGCTGGTCATTCAGGAGTCCATCCTGATGCTGCCTGAG
GAGGTGGAGGAAGTGATCGGCAACAAGCCAGAGTCTGACATCCTGGTGCACACCGCCTACGACGAGTCCA
CAGATGAGAATGTGATGCTGCTGACCTCTGACGCCCCCGAGTATAAGCCTTGGGCCCTGGTCATCCAGGATT
CTAACGGCGAGAATAAGATCAAGATGCTGAGCGGAGGATCCGGAGGATCTGGAGGCAGCACCAACCTGTC
TGACATCATCGAGAAGGAGACAGGCAAGCAGCTGGTCATCCAGGAGAGCATCCTGATGCTGCCCGAAGAA
GTCGAAGAAGTGATCGGAAACAAGCCTGAGAGCGATATCCTGGTCCATACCGCCTACGACGAGAGTACCGA
CGAAAATGTGATGCTGCTGACATCCGACGCCCCAGAGTATAAGCCCTGGGCTCTGGTCATCCAGGATTCCAA
CGGAGAGAACAAAATCAAAATGCTGTCTGGCGGCTCAAAAAGAACCGCCGACGGCAGCGAATTCGAGCCC
AAGAAGAAGAGGAAAGTCGGAAGCGGAGCTACTAACTTCAGCCTGCTGAAGCAGGCTGGAGACGTGGAG
GAGAACCCTGGACCTATGGTGAGCAAGGGCGAGGAGCTGTTCACCGGGGTGGTGCCCATCCTGGTCGAGC
TGGACGGCGACGTAAACGGCCACAAGTTCAGCGTGTCCGGCGAGGGCGAGGGCGATGCCACCTACGGCA
AGCTGACCCTGAAGTTCATCTGCACCACCGGCAAGCTGCCCGTGCCCTGGCCCACCCTCGTGACCACCCTG
ACCTATGGAGTGCAGTGCTTCAGCCGCTACCCCGACCACATGAAGCAGCACGACTTCTTCAAGTCCGCCATG

CCCGAAGGCTACGTCCAGGAGCGCACCATCTTCTTCAAGGACGACGGCAACTACAAGACCCGCGCCGAGG
TGAAGTTCGAGGGCGACACCCTGGTGAACCGCATCGAGCTGAAGGGCATCGACTTCAAGGAGGACGGCA
ACATCCTGGGGCACAAGCTGGAGTACAACTACAACAGCCACAACGTCTATATCATGGCCGACAAGCAGAAG
AACGGCATCAAGGTGAACTTCAAGATCCGCCACAACATCGAGGACGGCAGCGTGCAGCTCGCCGACCACTA
CCAGCAGAACACCCCCATCGGCGACGGCCCCGTGCTGCTGCCCGACAACCACTACCTGAGCACCCAGTCCG
CCCTGAGCAAAGACCCCAACGAGAAGCGCGATCACATGGTCCTGCTGGAGTTCGTGACCGCCGCCGGGAT
CACTCTCGGCATGGACGAGCTGTACAAG

A&C-BEmax-M-Rad51DBD

ATGAAGAGGACCGCCGATGGCTCTGAGTTCGAGAGCCCCAAGAAGAAGCGGAAGGTGGACAGCCTGCTG
ATGAACCGGAGAAAGTTCCTGTATCAGTTTAAGAATGTGAGGTGGGCAAAGGGCAGGCGCGAGACCTACC
TGTGCTATGTGGTGAAGCGGAGAGATAGCGCCACATCTTTCAGCCTGGACTTTGGCTACCTGCGGAACAAG
AATGGCTGCCACGTGGAGCTGCTGTTTCTGAGATACATCTCCGACTGGGATCTGGACCCAGGCAGGTGTTAT
CGCGTGACCTGGTTCACATCCTGGTCTCCCTGCTACGATTGTGCCCGGCACGTGGCCGACTTTCTGAGAGGC
AACCCTAATCTGTCTCTGAGGATCTTCACCGCCCGCCTGTATTTTTGCGAGGATAGGAAGGCCGAGCCAGAG
GGACTGAGGCGCCTGCACAGGGCCGGCGTGCAGATCGCCATCATGACATTCAAGGACTACTTTTATTGTTG
GAACACCTTCGTGGAGAATCACGAGCGGACATTTAAGGCCTGGGAGGGACTGCACGAGAACTCCGTGCGG
CTGTCTAGACAGCTGCGGAGAATCCTGCTGCCAGAGGCCGCCGCCAAGGAAGCTGCCGCCAAGGAGGCCG
CCGCCAAGAGCGAGGTGGAGTTCAGCCACGAGTACTGGATGAGACACGCCCTGACCCTGGCTAAGAGAGC
TTGGGATGAGAGAGAGGTGCCCGTGGGAGCTGTTCTGGTTCATAACAACAGGGTGATCGGCGAGGGATGG
AACAGACCTATCGGGAGACACGACCCAACCGCTCATGCTGAAATCATGGCCCTGAGACAAGGAGGGCTGG
TGATGCAAAATTACAGACTGATCGACGCAACCCTGTACGTGACCCTGGAGCCTTGTGTGATGTGCGCAGGA
GCAATGATCCACTCCAGAATCGGCAGAGTGGTGTTCGGAGCTAGAGATGCCAAAACCGGAGCCGCTGGAA
GCCTGATGGACGTTCTGCATCACCCCGGAATGAATCACAGAGTGGAGATAACCGAGGGCATTCTGGCCGAC
GAGTGTGCTGCTCTGCTGTCTGATTTCTTCAGAATGAGAAGGCAGGAAATCAAGGCCCAGAAAAAGGCCCA
AAGCAGCACCGACAGCGGAGGATCTAGCGGAGGATCAAGCGGAAGCGAGACTCCTGGAACCAGCGAAAG
CGCAACCCCAGAAAGCAGCGGAGGAAGTAGCGGAGGAAGCTCAGAAGTCGAGTTCAGCCATGAGTATTG
GATGAGACATGCTCTGACCCTGGCAAAGAGAGCAAGAGACGAGAGAGAGGTCCCAGTGGGAGCAGTTCT
GGTGCTGAACAACAGAGTGATCGGGGAGGGGTGGAACAGAGCAATCGGACTGCACGACCCTACAGCACA
CGCAGAAATAATGGCACTGAGACAAGGGGGGCTCGTGATGCAAAACTACAGGCTGATCGACGCCACCCTG
TACGTCACATTTGAGCCCTGTGTGATGTGTGCCGGAGCCATGATTCACAGTAGAATCGGCCGGGTGGTGTTC
GGTGTGAGAAACGCTAAAACAGGCGCCGCCGGAAGCCTGATGGATGTTCTGCATTACCCCGGCATGAATCA
CCGGGTGGAGATCACAGAGGGCATCCTGGCTGACGAATGTGCCGCTCTGCTGTGTTACTTCTTCAGAATGCC
CCGACAAGTGTTCAACGCCCAGAAGAAAGCCCAGTCAAGCACCGACTCTGGCGGATCTAGCGGTGGATCTA
GCGGCTCTGAGACCCCTGGAACATCCGAATCCGCCACTCCAGAGAGCAGCGGAGGCTCTTCTGGAGGATC
AGCAATGCAGATGCAGCTTGAAGCAAATGCAGATACTTCAGTGGAAGAAGAAAGCTTTGGCCCACAACCCA
TTTCACGGTTAGAGCAGTGTGGCATAAATGCCAACGATGTGAAGAAATTGGAAGAAGCTGGATTCCATACT
GTGGAGGCTGTTGCCTATGCGCCAAAGAAGGAGCTAATAAATATTAAGGGAATTAGTGAAGCCAAAGCTGA
TAAAATTCTGGCTGAGGCAGCTAAATTAGTTCCAATGGGTTTCACCACTGCAACTGAATTCCACCAAAGGCG
GTCAGAGATCATACAGATTACTACTGGCTCCAAAGAGCTTGACAAACTACTTCAATCCGGAGGATCTAGCGG
AGGCTCCTCTGGCTCTGAGACACCTGGCACAAGCGAGAGCGCAACACCTGAAAGCAGCGGGGGCAGCAG
CGGGGGGTCA--**Cas9n(Same as Cas9n in A&C-BEmax)**--AAAAGGCCGGCGGCCACGAAAAAGGCC
GGCCAGGCAAAAAAGAAAAAGGATCCGGAGAGGGCAGAGGAAGTCTGCTAACATGCGGTGACGTCGA
GGAGAATCCTGGCCCA--**2xUGI(Same as 2xUGI in A&C-BEmax)**--TCTGGCGGCTCAAAAAGAACCG

CCGACGGCAGCGAATTCGAGCCCAAGAAGAAGAGGAAAGTCGGAAGCGGAGCTACTAACTTCAGCCTGCTGAAGCAGGCTGGAGACGTGGAGGAGAACCCTGGACCT--GFP(Same as GFP in A&C-BEmax)

eA&C-BEmax

ATGAAACGGACAGCCGACGGAAGCGAGTTCGAGTCACCAAAGAAGAAGCGGAAAGTCGACAGCCTGCTGATGAACCGGAGAAAGTTCCTGTATCAGTTTAAGAATGTGAGGTGGGCAAAGGGCAGGCGCGAGACCTACCTGTGCTATGTGGTGAAGCGGAGAGATAGCGCCACATCTTTCAGCCTGGACTTTGGCTACCTGCGGAACAAGAATGGCTGCCACGTGGAGCTGCTGTTTCTGAGATACATCTCCGACTGGGATCTGGACCCAGGCAGGTGTTATCGCGTGACCTGGTTCACATCCTGGTCTCCCTGCTACGATTGTGCCCGGCACGTGGCCGACTTTCTGAGAGGCAACCCTAATCTGTCTCTGAGGATCTTCACCGCCCGCCTGTATTTTTGCGAGGATAGGAAGGCCGAGCCAGAGGGACTGAGGCGCCTGCACAGGGCCGGCGTGCAGATCGCCATCATGACATTCAAGGACTACTTTTATTGTTGGAACACCTTCGTGGAGAATCACGAGCGGACATTTAAGGCCTGGGAGGGACTGCACGAGAACTCCGTGCGGCTGTCTAGACAGCTGCGGAGAATCCTGCTGCCAGAGGCCGCCGCCAAGGAAGCTGCCGCCAAGGAGGCCGCCGCCAAGTCTGAGGTGGAGTTTTCCCACGAGTACTGGATGAGACATGCCCTGACCCTGGCCAAGAGGGCACGGGATGAGAGGGAGGTGCCTGTGGGAGCCGTGCTGGTGCTGAACAATAGAGTGATCGGCGAGGGCTGGAACAGAGCCATCGGCCTGCACGACCCAACAGCCCATGCCGAAATTATGGCCCTGAGACAGGGCGGCCTGGTCATGCAGAACTACAGACTGATTGACGCCACCCTGTACGTGACATTCGAGCCTTGCGTGATGTGCGCCGGCGCCATGATCCACTCTAGGATCGGCCGCGTGGTGTTTGGCGTGAGGAACTCAAAAAGAGGCGCCGCAGGCTCCCTGATGAACGTGCTGAACTACCCCGGCATGAATCACCGCGTCGAAATTACCGAGGGAATCCTGGCAGATGAATGTGCCGCCCTGCTGTGCGATTTCTATCGGATGCCTAGACAGGTGTTCAATGCTCAGAAGAAGGCCCAGAGCTCCATCAACTCCGGAGGATCTAGCGGAGGCTCCTCTGGCTCTGAGACACCTGGCACAAGCGAGAGCGCAACACCTGAAAGCAGCGGGGGCAGCAGCGGGGGGTCA--Cas9n(Same as Cas9n in A&C-BEmax)--AAAAGGCCGGCGGCCACGAAAAAGGCCGGCCAGGCAAAAAAGAAAAAGGGATCCGGAGAGGGCAGAGGAAGTCTGCTAACATGCGGTGACGTCGAGGAGAATCCTGGCCCA--2xUGI(Same as 2xUGI in A&C-BEmax)--TCTGGCGGCTCAAAAAGAACCGCCGACGGCAGCGAATTCGAGCCCAAGAAGAAGAGGAAAGTCGGAAGCGGAGCTACTAACTTCAGCCTGCTGAAGCAGGCTGGAGACGTGGAGGAGAACCCTGGACCT--GFP(Same as GFP in A&C-BEmax)

hyA&C-BEmax

ATGAAACGGACAGCCGACGGAAGCGAGTTCGAGTCACCAAAGAAGAAGCGGAAAGTCGACAGCCTGCTGATGAACCGGAGAAAGTTCCTGTATCAGTTTAAGAATGTGAGGTGGGCAAAGGGCAGGCGCGAGACCTACCTGTGCTATGTGGTGAAGCGGAGAGATAGCGCCACATCTTTCAGCCTGGACTTTGGCTACCTGCGGAACAAGAATGGCTGCCACGTGGAGCTGCTGTTTCTGAGATACATCTCCGACTGGGATCTGGACCCAGGCAGGTGTTATCGCGTGACCTGGTTCACATCCTGGTCTCCCTGCTACGATTGTGCCCGGCACGTGGCCGACTTTCTGAGAGGCAACCCTAATCTGTCTCTGAGGATCTTCACCGCCCGCCTGTATTTTTGCGAGGATAGGAAGGCCGAGCCAGAGGGACTGAGGCGCCTGCACAGGGCCGGCGTGCAGATCGCCATCATGACATTCAAGGACTACTTTTATTGTTGGAACACCTTCGTGGAGAATCACGAGCGGACATTTAAGGCCTGGGAGGGACTGCACGAGAACTCCGTGCGGCTGTCTAGACAGCTGCGGAGAATCCTGCTGCCAGAGGCCGCCGCCAAGGAAGCTGCCGCCAAGGAGGCCGCCGCCAAGTCTGAGGTGGAGTTTTCCCACGAGTACTGGATGAGACATGCCCTGACCCTGGCCAAGAGGGCACGGGATGAGAGGGAGGTGCCTGTGGGAGCCGTGCTGGTGCTGAACAATAGAGTGATCGGCGAGGGCTGGAACAGAGCCATCGGCCTGCACGACCCAACAGCCCATGCCGAAATTATGGCCCTGAGACAGGGCGGCCTGGTCATGCAGAACTACAGACTGATTGACGCCACCCTGTACGTGACATTCGAGCCTTGCGTGATGTGCGCCGGCGCCATGATCCACTCTAGGATCGGCCGCGTGGTGTTTGGCGTGAGGAACTCAAAAAGAGGCGCCGCAGGCTCCCTGATGAACGTGCTGAACTACCCCGGCATGAATCACCGCGTCGAAATTACCGAGGGAATCCTGGCAGATG

AATGTGCCGCCCTGCTGTGCGATTTCTATCGGATGCCTAGACAGGTGTTCAATGCTCAGAAGAAGGCCCAGA
GCTCCATCAACTCCGGAGGATCTAGCGGAGGCTCCTCTGGCTCTGAGACACCTGGCACAAGCGAGAGCGC
AACACCTGAAAGCAGCGGGGGCAGCAGCGGGGGGTCAGCAATGCAGATGCAGCTTGAAGCAAATGCAGA
TACTTCAGTGGAAGAAGAAAGCTTTGGCCCACAACCCATTTCACGGTTAGAGCAGTGTGGCATAAATGCCA
ACGATGTGAAGAAATTGGAAGAAGCTGGATTCCATACTGTGGAGGCTGTTGCCTATGCGCCAAAGAAGGAG
CTAATAAATATTAAGGGAATTAGTGAAGCCAAAGCTGATAAAATTCTGGCTGAGGCAGCTAAATTAGTTCCAA
TGGGTTTCACCACTGCAACTGAATTCCACCAAAGGCGGTCAGAGATCATACAGATTACTACTGGCTCCAAAG
AGCTTGACAAACTACTTCAATCCGGAGGATCTAGCGGAGGCTCCTCTGGCTCTGAGACACCTGGCACAAGC
GAGAGCGCAACACCTGAAAGCAGCGGGGGCAGCAGCGGGGGGTCA--**Cas9n**(**Same as Cas9n in A&C-BEmax**)--AAAAGGCCGGCGGCCACGAAAAAGGCCGGCCAGGCAAAAAAGAAAAAGGGATCCGGAGAG
GGCAGAGGAAGTCTGCTAACATGCGGTGACGTCGAGGAGAATCCTGGCCCA--**2xUGI**(**Same as 2xUGI in A&C-BEmax**)--TCTGGCGGCTCAAAAAGAACCGCCGACGGCAGCGAATTCGAGCCCAAGAAGAAGAG
GAAAGTCGGAAGCGGAGCTACTAACTTCAGCCTGCTGAAGCAGGCTGGAGACGTGGAGGAGAACCCTGG
ACCT--**GFP**(**Same as GFP in A&C-BEmax**)

Supplementary Note 4. DNA sequences used this study for HUDEP-2 transduction. Within lentivirus base editor sequences, hU6 sequences are highlighted in violet, *HBG 1/2* site 1 or *HBG 1/2* site 2 20bp sgRNA guide sequences are in gray, TadA8e sequences are in red, BpNLS sequences are in green, Rad51DBD sequences are in dark red, Cas9 nickase sequences are in cyan, linker sequences are in yellow, GFP sequences are in dark blue, puro sequences are in brown.

Linker-32aa: SGGSSGGSSGSETPGTSESATPESSGGSSGGS

TCCGGAGGATCTAGCGGAGGCTCCTCTGGCTCTGAGACACCTGGCACAAGCGAGAGCGCAACACCTGAAA
GCAGCGGGGGCAGCAGCGGGGGGTCA

Lenti HBG 1/2 site 1-ABE8e

GAGGGCCTATTTCCCATGATTCCTTCATATTTGCATATACGATACAAGGCTGTTAGAGAGATAATTAGAATTAA
TTTGACTGTAAACACAAAGATATTAGTACAAAATACGTGACGTAGAAAGTAATAATTTCTTGGGTAGTTTGCA
GTTTTAAAATTATGTTTTAAAATGGACTATCATATGCTTACCGTAACTTGAAAGTATTTCGATTTCTTGGCTTTA
TATATCTTGTGGAAAGGACGAAACACCGTTGACCAATAGCCTTGACAGTTTTAGAGCTAGAAATAGCAAGTT
AAAATAAGGCTAGTCCGTTATCAACTTGAAAAAGTGGCACCGAGTCGGTGCTTTTTTTGGTACCGAATTCGC
TAGCTAGGTCTTGAAAGGAGTGGGAATTGGCTCCGGTGCCCGTCAGTGGGCAGAGCGCACATCGCCCACA
GTCCCCGAGAAGTTGGGGGGAGGGGTCGGCAATTGATCCGGTGCCTAGAGAAGGTGGCGCGGGGTAAAC
TGGGAAAGTGATGTCGTGTACTGGCTCCGCCTTTTTCCCGAGGGTGGGGGAGAACCGTATATAAGTGCAGT
AGTCGCCGTGAACGTTCTTTTTCGCAACGGGTTTGCCGCCAGAACACAGGGGCGCGCCTCTAGAGCGATCG
CGCCACCATGAAACGGACAGCCGACGGAAGCGAGTTCGAGTCACCAAAGAAGAAGCGGAAAGTCTCTGA
GGTGGAGTTTTCCCACGAGTACTGGATGAGACATGCCCTGACCCTGGCCAAGAGGGCACGGGATGAGAGG
GAGGTGCCTGTGGGAGCCGTGCTGGTGCTGAACAATAGAGTGATCGGCGAGGGCTGGAACAGAGCCATC
GGCCTGCACGACCCAACAGCCCATGCCGAAATTATGGCCCTGAGACAGGGCGGCCTGGTCATGCAGAACTA
CAGACTGATTGACGCCACCCTGTACGTGACATTCGAGCCTTGCGTGATGTGCGCCGGCGCCATGATCCACTC
TAGGATCGGCCGCGTGGTGTTTGGCGTGAGGAACTCAAAAAGAGGCGCCGCAGGCTCCCTGATGAACGTG
CTGAACTACCCCGGCATGAATCACCGCGTCGAAATTACCGAGGGAATCCTGGCAGATGAATGTGCCGCCCTG
CTGTGCGATTTCTATCGGATGCCTAGACAGGTGTTCAATGCTCAGAAGAAGGCCCAGAGCTCCATCAACTCC
GGAGGATCTAGCGGAGGCTCCTCTGGCTCTGAGACACCTGGCACAAGCGAGAGCGCAACACCTGAAAGCA
GCGGGGGCAGCAGCGGGGGGTCAGACAAGAAGTACAGCATCGGCCTGGCCATCGGCACCAACTCTGTGG
GCTGGGCCGTGATCACCGACGAGTACAAGGTGCCCAGCAAGAAATTCAAGGTGCTGGGCAACACCGACCG
GCACAGCATCAAGAAGAACCTGATCGGAGCCCTGCTGTTCGACAGCGGCGAAACAGCCGAGGCCACCCGG
CTGAAGAGAACCGCCAGAAGAAGATACACCAGACGGAAGAACCGGATCTGCTATCTGCAAGAGATCTTCA
GCAACGAGATGGCCAAGGTGGACGACAGCTTCTTCCACAGACTGGAAGAGTCCTTCCTGGTGGAAGAGGA
TAAGAAGCACGAGCGGCACCCCATCTTCGGCAACATCGTGGACGAGGTGGCCTACCACGAGAAGTACCCC
ACCATCTACCACCTGAGAAAGAAACTGGTGGACAGCACCGACAAGGCCGACCTGCGGCTGATCTATCTGGC
CCTGGCCCACATGATCAAGTTCCGGGGCCACTTCCTGATCGAGGGCGACCTGAACCCCGACAACAGCGACG
TGGACAAGCTGTTCATCCAGCTGGTGCAGACCTACAACCAGCTGTTCGAGGAAAACCCCATCAACGCCAGC
GGCGTGGACGCCAAGGCCATCCTGTCTGCCAGACTGAGCAAGAGCAGACGGCTGGAAAATCTGATCGCCC
AGCTGCCCGGCGAGAAGAAGAATGGCCTGTTCGGAAACCTGATTGCCCTGAGCCTGGGCCTGACCCCCAA
CTTCAAGAGCAACTTCGACCTGGCCGAGGATGCCAAACTGCAGCTGAGCAAGGACACCTACGACGACGAC
CTGGACAACCTGCTGGCCCAGATCGGCGACCAGTACGCCGACCTGTTTCTGGCCGCCAAGAACCTGTCCGA
CGCCATCCTGCTGAGCGACATCCTGAGAGTGAACACCGAGATCACCAAGGCCCCCCTGAGCGCCTCTATGAT
CAAGAGATACGACGAGCACCACCAGGACCTGACCCTGCTGAAAGCTCTCGTGCGGCAGCAGCTGCCTGAG

AAGTACAAAGAGATTTTCTTCGACCAGAGCAAGAACGGCTACGCCGGCTACATTGACGGCGGAGCCAGCC
AGGAAGAGTTCTACAAGTTCATCAAGCCCATCCTGGAAAAGATGGACGGCACCGAGGAACTGCTCGTGAA
GCTGAACAGAGAGGACCTGCTGCGGAAGCAGCGGACCTTCGACAACGGCAGCATCCCCCACCAGATCCAC
CTGGGAGAGCTGCACGCCATTCTGCGGCGGCAGGAAGATTTTTACCCATTCCTGAAGGACAACCGGGAAA
AGATCGAGAAGATCCTGACCTTCCGCATCCCCTACTACGTGGGCCCTCTGGCCAGGGGAAACAGCAGATTC
GCCTGGATGACCAGAAAGAGCGAGGAAACCATCACCCCCTGGAACTTCGAGGAAGTGGTGGACAAGGGC
GCTTCCGCCCAGAGCTTCATCGAGCGGATGACCAACTTCGATAAGAACCTGCCCAACGAGAAGGTGCTGCC
CAAGCACAGCCTGCTGTACGAGTACTTCACCGTGTATAACGAGCTGACCAAAGTGAAATACGTGACCGAGG
GAATGAGAAAGCCCGCCTTCCTGAGCGGCGAGCAGAAAAAGGCCATCGTGGACCTGCTGTTCAAGACCAA
CCGGAAAGTGACCGTGAAGCAGCTGAAAGAGGACTACTTCAAGAAAATCGAGTGCTTCGACTCCGTGGAA
ATCTCCGGCGTGGAAGATCGGTTCAACGCCTCCCTGGGCACATACCACGATCTGCTGAAAATTATCAAGGAC
AAGGACTTCCTGGACAATGAGGAAAACGAGGACATTCTGGAAGATATCGTGCTGACCCTGACACTGTTTGA
GGACAGAGAGATGATCGAGGAACGGCTGAAAACCTATGCCCACCTGTTCGACGACAAAGTGATGAAGCAG
CTGAAGCGGCGGAGATACACCGGCTGGGGCAGGCTGAGCCGGAAGCTGATCAACGGCATCCGGGACAAG
CAGTCCGGCAAGACAATCCTGGATTTCCTGAAGTCCGACGGCTTCGCCAACAGAAACTTCATGCAGCTGATC
CACGACGACAGCCTGACCTTTAAAGAGGACATCCAGAAAGCCCAGGTGTCCGGCCAGGGCGATAGCCTGC
ACGAGCACATTGCCAATCTGGCCGGCAGCCCCGCCATTAAGAAGGGCATCCTGCAGACAGTGAAGGTGGT
GGACGAGCTCGTGAAAGTGATGGGCCGGCACAAGCCCGAGAACATCGTGATCGAAATGGCCAGAGAGAA
CCAGACCACCCAGAAGGGACAGAAGAACAGCCGCGAGAGAATGAAGCGGATCGAAGAGGGCATCAAAG
AGCTGGGCAGCCAGATCCTGAAAGAACACCCCGTGGAAAACACCCAGCTGCAGAACGAGAAGCTGTACCT
GTACTACCTGCAGAATGGGCGGGATATGTACGTGGACCAGGAACTGGACATCAACCGGCTGTCCGACTACG
ATGTGGACCATATCGTGCCTCAGAGCTTTCTGAAGGACGACTCCATCGACAACAAGGTGCTGACCAGAAGC
GACAAGAACCGGGGCAAGAGCGACAACGTGCCCTCCGAAGAGGTCGTGAAGAAGATGAAGAACTACTGG
CGGCAGCTGCTGAACGCCAAGCTGATTACCCAGAGAAAGTTCGACAATCTGACCAAGGCCGAGAGAGGCG
GCCTGAGCGAACTGGATAAGGCCGGCTTCATCAAGAGACAGCTGGTGGAAACCCGGCAGATCACAAAGCA
CGTGGCACAGATCCTGGACTCCCGGATGAACACTAAGTACGACGAGAATGACAAGCTGATCCGGGAAGTG
AAAGTGATCACCCTGAAGTCCAAGCTGGTGTCCGATTTCCGGAAGGATTTCCAGTTTTACAAAGTGCGCGA
GATCAACAACTACCACCACGCCCACGACGCCTACCTGAACGCCGTCGTGGGAACCGCCCTGATCAAAAAGT
ACCCTAAGCTGGAAAGCGAGTTCGTGTACGGCGACTACAAGGTGTACGACGTGCGGAAGATGATCGCCAA
GAGCGAGCAGGAAATCGGCAAGGCTACCGCCAAGTACTTCTTCTACAGCAACATCATGAACTTTTTCAAGA
CCGAGATTACCCTGGCCAACGGCGAGATCCGGAAGCGGCCTCTGATCGAGACAAACGGCGAAACCGGGG
AGATCGTGTGGGATAAGGGCCGGGATTTTGCCACCGTGCGGAAAGTGCTGAGCATGCCCCAAGTGAATATC
GTGAAAAAGACCGAGGTGCAGACAGGCGGCTTCAGCAAAGAGTCTATCCTGCCCAAGAGGAACAGCGATA
AGCTGATCGCCAGAAAGAAGGACTGGGACCCTAAGAAGTACGGCGGCTTCGACAGCCCCACCGTGGCCTA
TTCTGTGCTGGTGGTGGCCAAAGTGGAAAAGGGCAAGTCCAAGAAACTGAAGAGTGTGAAAGAGCTGCT
GGGGATCACCATCATGGAAAGAAGCAGCTTCGAGAAGAATCCCATCGACTTTCTGGAAGCCAAGGGCTACA
AAGAAGTGAAAAAGGACCTGATCATCAAGCTGCCTAAGTACTCCCTGTTCGAGCTGGAAAACGGCCGGAA
GAGAATGCTGGCCTCTGCCGGCGAACTGCAGAAGGGAAACGAACTGGCCCTGCCCTCCAAATATGTGAACT
TCCTGTACCTGGCCAGCCACTATGAGAAGCTGAAGGGCTCCCCCGAGGATAATGAGCAGAAACAGCTGTTT
GTGGAACAGCACAAGCACTACCTGGACGAGATCATCGAGCAGATCAGCGAGTTCTCCAAGAGAGTGATCCT
GGCCGACGCTAATCTGGACAAAGTGCTGTCCGCCTACAACAAGCACCGGGATAAGCCCATCAGAGAGCAG
GCCGAGAATATCATCCACCTGTTTACCCTGACCAATCTGGGAGCCCCTGCCGCCTTCAAGTACTTTGACACCA
CCATCGACCGGAAGAGGTACACCAGCACCAAAGAGGTGCTGGACGCCACCCTGATCCACCAGAGCATCAC
CGGCCTGTACGAGACACGGATCGACCTGTCTCAGCTGGGAGGCGACAAAAGGCCGGCGGCCACGAAAAA

GGCCGGCCAGGCAAAAAAGAAAAAGGCAACAAACTTCTCTCTGCTGAAACAAGCCGGAGATGTCGAAGA
GAATCCTGGACCGCTCGAGATGGTGAGCAAGGGCGAGGAGCTGTTCACCGGGGTGGTGCCCATCCTGGTC
GAGCTGGACGGCGACGTAAACGGCCACAAGTTCAGCGTGTCCGGCGAGGGCGAGGGCGATGCCACCTAC
GGCAAGCTGACCCTGAAGTTCATCTGCACCACCGGCAAGCTGCCCGTGCCCTGGCCCACCCTCGTGACCAC
CCTGACCTACGGCGTGCAGTGCTTCAGCCGCTACCCCGACCACATGAAGCAGCACGACTTCTTCAAGTCCGC
CATGCCCGAAGGCTACGTCCAGGAGCGCACCATCTTCTTCAAGGACGACGGCAACTACAAGACCCGCGCCG
AGGTGAAGTTCGAGGGCGACACCCTGGTGAACCGCATCGAGCTGAAGGGCATCGACTTCAAGGAGGACG
GCAACATCCTGGGGCACAAGCTGGAGTACAACTACAACAGCCACAACGTCTATATCATGGCCGACAAGCAG
AAGAACGGCATCAAGGTGAACTTCAAGATCCGCCACAACATCGAGGACGGCAGCGTGCAGCTCGCCGACC
ACTACCAGCAGAACACCCCCATCGGCGACGGCCCCGTGCTGCTGCCCGACAACCACTACCTGAGCACCCAG
TCCGCCCTGAGCAAAGACCCCAACGAGAAGCGCGATCACATGGTCCTGCTGGAGTTCGTGACCGCCGCCG
GGATCACTCTCGGCATGGACGAGCTGTACAAGTACTCAGATCTGGAGCTCAAGTGAGCTAGCCTACCGGGT
AGGGGAGGCGCTTTTCCCAAGGCAGTCTGGAGCATGCGCTTTAGCAGCCCCGCTGGGCACTTGGCGCTAC
ACAAGTGGCCTCTGGCCTCGCACACATTCCACATCCACCGGTAGGCGCCAACCGGCTCCGTTCTTTGGTGGC
CCCTTCGCGCCACCTTCTACTCCTCCCCTAGTCAGGAAGTTCCCCCCCGCCCCGCAGCTCGCGTCGTGCAGG
ACGTGACAAATGGAAGTAGCACGTCTCACTAGTCTCGTGCAGATGGACAGCACCGCTGAGCAATGGAAGCG
GGTAGGCCTTTGGGGCAGCGGCCAATAGCAGCTTTGCTCCTTCGCTTTCTGGGCTCAGAGGCTGGGAAGG
GGTGGGTCCGGGGGCGGGCTCAGGGGCGGGCTCAGGGGCGGGGCGGGCGCCCGAAGGTCCTCCGGAG
GCCCGGCATTCTGCACGCTTCAAAAGCGCACGTCTGCCGCGCTGTTCTCCTCTTCCTCATCTCCGGGCCTTTC
GGAATTCGCCACCATGACCGAGTACAAGCCCACGGTGCGCCTCGCCACCCGCGACGACGTCCCCAGGGCC
GTACGCACCCTCGCCGCCGCGTTCGCCGACTACCCCGCCACGCGCCACACCGTCGATCCGGACCGCCACATC
GAGCGGGTCACCGAGCTGCAAGAACTCTTCCTCACGCGCGTCGGGCTCGACATCGGCAAGGTGTGGGTCG
CGGACGACGGCGCCGCGGTGGCGGTCTGGACCACGCCGGAGAGCGTCGAAGCGGGGGCGGTGTTCGCC
GAGATCGGCCCGCGCATGGCCGAGTTGAGCGGTTCCCGGCTGGCCGCGCAGCAACAGATGGAAGGCCTCC
TGGCGCCGCACCGGCCCAAGGAGCCCGCGTGGTTCCTGGCCACCGTCGGAGTCTCGCCCGACCACCAGGG
CAAGGGTCTGGGCAGCGCCGTCGTGCTCCCCGGAGTGGAGGCGGCCGAGCGCGCCGGGGTGCCCGCCTT
CCTGGAGACCTCCGCGCCCCGCAACCTCCCCTTCTACGAGCGGCTCGGCTTCACCGTCACCGCCGACGTCG
AGGTGCCCGAAGGACCGCGCACCTGGTGCATGACCCGCAAGCCCGGTGCCTGA

Lenti HBG 1/2 site 1-hyABE

GAGGGCCTATTTCCCATGATTCCTTCATATTTGCATATACGATACAAGGCTGTTAGAGAGATAATTAGAATTAA
TTTGACTGTAAACACAAAGATATTAGTACAAAATACGTGACGTAGAAAGTAATAATTTCTTGGGTAGTTTGCA
GTTTTAAAATTATGTTTTAAAATGGACTATCATATGCTTACCGTAACTTGAAAGTATTTCGATTTCTTGGCTTTA
TATATCTTGTGGAAAGGACGAAACACCGTTGACCAATAGCCTTGACAGTTTTAGAGCTAGAAATAGCAAGTT
AAAATAAGGCTAGTCCGTTATCAACTTGAAAAAGTGGCACCGAGTCGGTGCTTTTTTTGGTACCGAATTCGC
TAGCTAGGTCTTGAAAGGAGTGGGAATTGGCTCCGGTGCCCGTCAGTGGGCAGAGCGCACATCGCCCACA
GTCCCCGAGAAGTTGGGGGGAGGGGTCGGCAATTGATCCGGTGCCTAGAGAAGGTGGCGCGGGGTAAAC
TGGGAAAGTGATGTCGTGTACTGGCTCCGCCTTTTTCCCGAGGGTGGGGGAGAACCGTATATAAGTGCAGT
AGTCGCCGTGAACGTTCTTTTTCGCAACGGGTTTGCCGCCAGAACACAGGGGCGCGCCTCTAGAGCGATCG
CGCCACCATGAAACGGACAGCCGACGGAAGCGAGTTCGAGTCACCAAAGAAGAAGCGGAAAGTCTCTGA
GGTGGAGTTTTCCCACGAGTACTGGATGAGACATGCCCTGACCCTGGCCAAGAGGGCACGGGATGAGAGG
GAGGTGCCTGTGGGAGCCGTGCTGGTGCTGAACAATAGAGTGATCGGCGAGGGCTGGAACAGAGCCATC
GGCCTGCACGACCCAACAGCCCATGCCGAAATTATGGCCCTGAGACAGGGCGGCCTGGTCATGCAGAACTA
CAGACTGATTGACGCCACCCTGTACGTGACATTCGAGCCTTGCGTGATGTGCGCCGGCGCCATGATCCACTC

TAGGATCGGCCGCGTGGTGTTTGGCGTGAGGAACTCAAAAAGAGGCGCCGCAGGCTCCCTGATGAACGTG
CTGAACTACCCCGGCATGAATCACCGCGTCGAAATTACCGAGGGAATCCTGGCAGATGAATGTGCCGCCCTG
CTGTGCGATTTCTATCGGATGCCTAGACAGGTGTTCAATGCTCAGAAGAAGGCCCAGAGCTCCATCAACTCC
GGAGGATCTAGCGGAGGCTCCTCTGGCTCTGAGACACCTGGCACAAGCGAGAGCGCAACACCTGAAAGCA
GCGGGGGCAGCAGCGGGGGGTCAGCAATGCAGATGCAGCTTGAAGCAAATGCAGATACTTCAGTGGAAG
AAGAAAGCTTTGGCCCACAACCCATTTCACGGTTAGAGCAGTGTGGCATAAATGCCAACGATGTGAAGAAA
TTGGAAGAAGCTGGATTCCATACTGTGGAGGCTGTTGCCTATGCGCCAAAGAAGGAGCTAATAAATATTAAG
GGAATTAGTGAAGCCAAAGCTGATAAAATTCTGGCTGAGGCAGCTAAATTAGTTCCAATGGGTTTCACCACT
GCAACTGAATTCCACCAAAGGCGGTCAGAGATCATACAGATTACTACTGGCTCCAAAGAGCTTGACAAACTA
CTTCAATCCGGAGGATCTAGCGGAGGCTCCTCTGGCTCTGAGACACCTGGCACAAGCGAGAGCGCAACAC
CTGAAAGCAGCGGGGGCAGCAGCGGGGGGTCA--Cas9n**(Same as Cas9n in Lenti HBG 1/2 site 1-ABE8e)**--AAAAGGCCGGCGGCCACGAAAAAGGCCGGCCAGGCAAAAAAGAAAAAGGCAACAAACTTCT
CTCTGCTGAAACAAGCCGGAGATGTCGAAGAGAATCCTGGACCGCTCGAG--GFP**(Same as GFP in Lenti HBG 1/2 site 1-ABE8e)**--GCTAGCCTACCGGGTAGGGGAGGCGCTTTTCCCAAGGCAGTCTGGAGC
ATGCGCTTTAGCAGCCCCGCTGGGCACTTGGCGCTACACAAGTGGCCTCTGGCCTCGCACACATTCCACATC
CACCGGTAGGCGCCAACCGGCTCCGTTCTTTGGTGGCCCCTTCGCGCCACCTTCTACTCCTCCCCTAGTCAG
GAAGTTCCCCCCCGCCCCGCAGCTCGCGTCGTGCAGGACGTGACAAATGGAAGTAGCACGTCTCACTAGTC
TCGTGCAGATGGACAGCACCGCTGAGCAATGGAAGCGGGTAGGCCTTTGGGGCAGCGGCCAATAGCAGCT
TTGCTCCTTCGCTTTCTGGGCTCAGAGGCTGGGAAGGGGTGGGTCCGGGGGCGGGCTCAGGGGCGGGCT
CAGGGGCGGGGCGGGCGCCCGAAGGTCCTCCGGAGGCCCGGCATTCTGCACGCTTCAAAAGCGCACGTC
TGCCGCGCTGTTCTCCTCTTCCTCATCTCCGGGCCTTTCGGAATTCGCCACCATGACCGAGTACAAGCCCACG
GTGCGCCTCGCCACCCGCGACGACGTCCCCAGGGCCGTACGCACCCTCGCCGCCGCGTTCGCCGACTACCC
CGCCACGCGCCACACCGTCGATCCGGACCGCCACATCGAGCGGGTCACCGAGCTGCAAGAACTCTTCCTCA
CGCGCGTCGGGCTCGACATCGGCAAGGTGTGGGTCGCGGACGACGGCGCCGCGGTGGCGGTCTGGACCA
CGCCGGAGAGCGTCGAAGCGGGGGCGGTGTTCGCCGAGATCGGCCCGCGCATGGCCGAGTTGAGCGGTT
CCCGGCTGGCCGCGCAGCAACAGATGGAAGGCCTCCTGGCGCCGCACCGGCCCAAGGAGCCCGCGTGGT
TCCTGGCCACCGTCGGAGTCTCGCCCGACCACCAGGGCAAGGGTCTGGGCAGCGCCGTCGTGCTCCCCGG
AGTGGAGGCGGCCGAGCGCGCCGGGGTGCCCGCCTTCCTGGAGACCTCCGCGCCCCGCAACCTCCCCTTC
TACGAGCGGCTCGGCTTCACCGTCACCGCCGACGTCGAGGTGCCCGAAGGACCGCGCACCTGGTGCATGA
CCCGCAAGCCCGGTGCCTGA

Lenti HBG 1/2 site 2-ABE8e

GAGGGCCTATTTCCCATGATTCCTTCATATTTGCATATACGATACAAGGCTGTTAGAGAGATAATTAGAATTAA
TTTGACTGTAAACACAAAGATATTAGTACAAAATACGTGACGTAGAAAGTAATAATTTCTTGGGTAGTTTGCA
GTTTTAAAATTATGTTTTAAAATGGACTATCATATGCTTACCGTAACTTGAAAGTATTTCGATTTCTTGGCTTTA
TATATCTTGTGGAAAGGACGAAACACCGTGGGGAAGGGGCCCCCAAGGTTTTAGAGCTAGAAATAGCAAG
TTAAAATAAGGCTAGTCCGTTATCAACTTGAAAAAGTGGCACCGAGTCGGTGCTTTTTTTGGTACCGAATTC
GCTAGCTAGGTCTTGAAAGGAGTGGGAATTGGCTCCGGTGCCCGTCAGTGGGCAGAGCGCACATCGCCCA
CAGTCCCCGAGAAGTTGGGGGGAGGGGTCGGCAATTGATCCGGTGCCTAGAGAAGGTGGCGCGGGGTAA
ACTGGGAAAGTGATGTCGTGTACTGGCTCCGCCTTTTTCCCGAGGGTGGGGGAGAACCGTATATAAGTGCA
GTAGTCGCCGTGAACGTTCTTTTTCGCAACGGGTTTGCCGCCAGAACACAGGGGCGCGCCTCTAGAGCGAT
CGCGCCACCATGAAACGGACAGCCGACGGAAGCGAGTTCGAGTCACCAAAGAAGAAGCGGAAAGTCTCT
GAGGTGGAGTTTTCCCACGAGTACTGGATGAGACATGCCCTGACCCTGGCCAAGAGGGCACGGATGAGA
GGGAGGTGCCTGTGGGAGCCGTGCTGGTGCTGAACAATAGAGTGATCGGCGAGGGCTGGAACAGAGCCA

TCGGCCTGCACGACCCAACAGCCCATGCCGAAATTATGGCCCTGAGACAGGGCGGCCTGGTCATGCAGAAC
TACAGACTGATTGACGCCACCCTGTACGTGACATTCGAGCCTTGCGTGATGTGCGCCGGCGCCATGATCCAC
TCTAGGATCGGCCGCGTGGTGTTTGGCGTGAGGAACTCAAAAAGAGGCGCCGCAGGCTCCCTGATGAACG
TGCTGAACTACCCCGGCATGAATCACCGCGTCGAAATTACCGAGGGAATCCTGGCAGATGAATGTGCCGCCC
TGCTGTGCGATTTCTATCGGATGCCTAGACAGGTGTTCAATGCTCAGAAGAAGGCCCAGAGCTCCATCAAC
TCCGGAGGATCTAGCGGAGGCTCCTCTGGCTCTGAGACACCTGGCACAAGCGAGAGCGCAACACCTGAAA
GCAGCGGGGGCAGCAGCGGGGGGTCA--Cas9n**(Same as Cas9n in Lenti HBG 1/2 site 1-ABE8e)**--AAAAGGCCGGCGGCCACGAAAAAGGCCGGCCAGGCAAAAAAGAAAAAGGCAACAAACTTCTCTCTG
CTGAAACAAGCCGGAGATGTCGAAGAGAATCCTGGACCGCTCGAG--GFP**(Same as GFP in Lenti HBG 1/2 site 1-ABE8e)**--GCTAGCCTACCGGGTAGGGGAGGCGCTTTTCCCAAGGCAGTCTGGAGCATGCG
CTTTAGCAGCCCCGCTGGGCACTTGGCGCTACACAAGTGGCCTCTGGCCTCGCACACATTCCACATCCACCG
GTAGGCGCCAACCGGCTCCGTTCTTTGGTGGCCCCTTCGCGCCACCTTCTACTCCTCCCCTAGTCAGGAAGT
TCCCCCCCGCCCCGCAGCTCGCGTCGTGCAGGACGTGACAAATGGAAGTAGCACGTCTCACTAGTCTCGTG
CAGATGGACAGCACCGCTGAGCAATGGAAGCGGGTAGGCCTTTGGGGCAGCGGCCAATAGCAGCTTTGCT
CCTTCGCTTTCTGGGCTCAGAGGCTGGGAAGGGGTGGGTCCGGGGGCGGGCTCAGGGGCGGGCTCAGG
GGCGGGGCGGGCGCCCGAAGGTCCTCCGGAGGCCCGGCATTCTGCACGCTTCAAAAGCGCACGTCTGCC
GCGCTGTTCTCCTCTTCCTCATCTCCGGGCCTTTCGGAATTCGCCACCATGACCGAGTACAAGCCCACGGTG
CGCCTCGCCACCCGCGACGACGTCCCCAGGGCCGTACGCACCCTCGCCGCCGCGTTCGCCGACTACCCCGC
CACGCGCCACACCGTCGATCCGGACCGCCACATCGAGCGGGTCACCGAGCTGCAAGAACTCTTCCTCACGC
GCGTCGGGCTCGACATCGGCAAGGTGTGGGTCGCGGACGACGGCGCCGCGGTGGCGGTCTGGACCACGC
CGGAGAGCGTCGAAGCGGGGGCGGTGTTCGCCGAGATCGGCCCGCGCATGGCCGAGTTGAGCGGTTCCC
GGCTGGCCGCGCAGCAACAGATGGAAGGCCTCCTGGCGCCGCACCGGCCCAAGGAGCCCGCGTGGTTCC
TGGCCACCGTCGGAGTCTCGCCCGACCACCAGGGCAAGGGTCTGGGCAGCGCCGTCGTGCTCCCCGGAGT
GGAGGCGGCCGAGCGCGCCGGGGTGCCCGCCTTCCTGGAGACCTCCGCGCCCCGCAACCTCCCCTTCTAC
GAGCGGCTCGGCTTCACCGTCACCGCCGACGTCGAGGTGCCCGAAGGACCGCGCACCTGGTGCATGACCC
GCAAGCCCGGTGCCTGA

Lenti HBG 1/2 site 2-hyABE

GAGGGCCTATTTCCCATGATTCCTTCATATTTGCATATACGATACAAGGCTGTTAGAGAGATAATTAGAATTAA
TTTGACTGTAAACACAAAGATATTAGTACAAAATACGTGACGTAGAAAGTAATAATTTCTTGGGTAGTTTGCA
GTTTTAAAATTATGTTTTAAAATGGACTATCATATGCTTACCGTAACTTGAAAGTATTTCGATTTCTTGGCTTTA
TATATCTTGTGGAAAGGACGAAACACCGTGGGGAAGGGGCCCCCAAGGTTTTAGAGCTAGAAATAGCAAG
TTAAAATAAGGCTAGTCCGTTATCAACTTGAAAAAGTGGCACCGAGTCGGTGCTTTTTTTGGTACCGAATTC
GCTAGCTAGGTCTTGAAAGGAGTGGGAATTGGCTCCGGTGCCCGTCAGTGGGCAGAGCGCACATCGCCCA
CAGTCCCCGAGAAGTTGGGGGGAGGGGTCGGCAATTGATCCGGTGCCTAGAGAAGGTGGCGCGGGGTAA
ACTGGGAAAGTGATGTCGTGTACTGGCTCCGCCTTTTTCCCGAGGGTGGGGGAGAACCGTATATAAGTGCA
GTAGTCGCCGTGAACGTTCTTTTTCGCAACGGGTTTGCCGCCAGAACACAGGGGCGCGCCTCTAGAGCGAT
CGCGCCACCATGAAACGGACAGCCGACGGAAGCGAGTTCGAGTCACCAAAGAAGAAGCGGAAAGTCTCT
GAGGTGGAGTTTTCCCACGAGTACTGGATGAGACATGCCCTGACCCTGGCCAAGAGGGCACGGGATGAGA
GGGAGGTGCCTGTGGGAGCCGTGCTGGTGCTGAACAATAGAGTGATCGGCGAGGGCTGGAACAGAGCCA
TCGGCCTGCACGACCCAACAGCCCATGCCGAAATTATGGCCCTGAGACAGGGCGGCCTGGTCATGCAGAAC
TACAGACTGATTGACGCCACCCTGTACGTGACATTCGAGCCTTGCGTGATGTGCGCCGGCGCCATGATCCAC
TCTAGGATCGGCCGCGTGGTGTTTGGCGTGAGGAACTCAAAAAGAGGCGCCGCAGGCTCCCTGATGAACG
TGCTGAACTACCCCGGCATGAATCACCGCGTCGAAATTACCGAGGGAATCCTGGCAGATGAATGTGCCGCCC

TGCTGTGCGATTTCTATCGGATGCCTAGACAGGTGTTCAATGCTCAGAAGAAGGCCCAGAGCTCCATCAACT
CCGGAGGATCTAGCGGAGGCTCCTCTGGCTCTGAGACACCTGGCACAAGCGAGAGCGCAACACCTGAAAG
CAGCGGGGGCAGCAGCGGGGGGTCAGCAATGCAGATGCAGCTTGAAGCAAATGCAGATACTTCAGTGGA
AGAAGAAAGCTTTGGCCCACAACCCATTTCACGGTTAGAGCAGTGTGGCATAAATGCCAACGATGTGAAGA
AATTGGAAGAAGCTGGATTCCATACTGTGGAGGCTGTTGCCTATGCGCCAAAGAAGGAGCTAATAAATATTA
AGGGAATTAGTGAAGCCAAAGCTGATAAAATTCTGGCTGAGGCAGCTAAATTAGTTCCAATGGGTTTCACCA
CTGCAACTGAATTCCACCAAAGGCGGTCAGAGATCATACAGATTACTACTGGCTCCAAAGAGCTTGACAAAC
TACTTCAATCCGGAGGATCTAGCGGAGGCTCCTCTGGCTCTGAGACACCTGGCACAAGCGAGAGCGCAACA
CCTGAAAGCAGCGGGGGCAGCAGCGGGGGGTCA--**Cas9n(Same as Cas9n in Lenti HBG 1/2 site
1-ABE8e)**--AAAAGGCCGGCGGCCACGAAAAAGGCCGGCCAGGCAAAAAAGAAAAAGGCAACAAACTTC
TCTCTGCTGAAACAAGCCGGAGATGTCGAAGAGAATCCTGGACCGCTCGAG--**GFP(Same as GFP in Le
nti HBG 1/2 site 1-ABE8e)**--GCTAGCCTACCGGGTAGGGGAGGCGCTTTTCCCAAGGCAGTCTGGAGC
ATGCGCTTTAGCAGCCCCGCTGGGCACTTGGCGCTACACAAGTGGCCTCTGGCCTCGCACACATTCCACATC
CACCGGTAGGCGCCAACCGGCTCCGTTCTTTGGTGGCCCCTTCGCGCCACCTTCTACTCCTCCCCTAGTCAG
GAAGTTCCCCCCCGCCCCGCAGCTCGCGTCGTGCAGGACGTGACAAATGGAAGTAGCACGTCTCACTAGTC
TCGTGCAGATGGACAGCACCGCTGAGCAATGGAAGCGGGTAGGCCTTTGGGGCAGCGGCCAATAGCAGCT
TTGCTCCTTCGCTTTCTGGGCTCAGAGGCTGGGAAGGGGTGGGTCCGGGGGCGGGCTCAGGGGCGGGCT
CAGGGGCGGGGCGGGCGCCCGAAGGTCCTCCGGAGGCCCGGCATTCTGCACGCTTCAAAAGCGCACGTC
TGCCGCGCTGTTCTCCTCTTCCTCATCTCCGGGCCTTTCGGAATTCGCCACCATGACCGAGTACAAGCCCACG
GTGCGCCTCGCCACCCGCGACGACGTCCCCAGGGCCGTACGCACCCTCGCCGCCGCGTTCGCCGACTACCC
CGCCACGCGCCACACCGTCGATCCGGACCGCCACATCGAGCGGGTCACCGAGCTGCAAGAACTCTTCCTCA
CGCGCGTCGGGCTCGACATCGGCAAGGTGTGGGTCGCGGACGACGGCGCCGCGGTGGCGGTCTGGACCA
CGCCGGAGAGCGTCGAAGCGGGGGCGGTGTTCGCCGAGATCGGCCCGCGCATGGCCGAGTTGAGCGGTT
CCCGGCTGGCCGCGCAGCAACAGATGGAAGGCCTCCTGGCGCCGCACCGGCCCAAGGAGCCCGCGTGGT
TCCTGGCCACCGTCGGAGTCTCGCCCGACCACCAGGGCAAGGGTCTGGGCAGCGCCGTCGTGCTCCCCGG
AGTGGAGGCGGCCGAGCGCGCCGGGGTGCCCGCCTTCCTGGAGACCTCCGCGCCCCGCAACCTCCCCTTC
TACGAGCGGCTCGGCTTCACCGTCACCGCCGACGTCGAGGTGCCCGAAGGACCGCGCACCTGGTGCATGA
CCCGCAAGCCCGGTGCCTGA

Supplementary Table 1. Phenotypes of zebrafish embryos injected with ABEs at various concentrations.

	mRNA Concentration (ng/μl)	Number of injected embryos	Number of survived embryos (%)	Number of embryos with abnormal phenotypes (%)
**Control**	Nuclease-free water	63	59（93.7%）	1（1.7%）
**ABEmax**	100	72	65（90.3%）	4（6.2%）
	200	64	58（90.6%）	5（8.6%）
	400	119	42（35.3%）	12（28.6%）
**ABE8e**	100	79	70（88.6%）	3（4.3%）
	200	53	46（86.8%）	3（6.5%）
	400	80	31（38.8%）	10（32.3%）
**hyABE**	100	63	57（90.5%）	3（5.3%）
	200	65	56（86.2%）	4（7.1%）
	400	110	54（49.1%）	13（24.1%）

Supplementary Table 2. Percentages of survived embryos with hyABE or ABEmax injections.

	**Mutation type**	**Number of injected embryos**	**Number of survived embryos (%)**
**hyABE**	*rps14*^{E12G}	183	163 (89.1%)
**ABEmax**	*rps14*^{E12G}	110	89 (80.9%)

Supplementary Table 3. Genotypes of zebrafish embryos injected with hyABE/*rps14* sgRNA or ABEmax/*rps14* sgRNA.

	**Mutation type**	**Homozygote （G=100%）**	**High mosaic （100%>G>50%）**	**Low mosaic （G<50%）**
**hyABE**	$rps14^{E12G}$	13.9% (11/79)	59.5% (47/79)	26.6% (21/79)
**ABEmax**	$rps14^{E12G}$	0% (0/78)	0% (0/78)	66.6% (52/78)

Supplementary Table 4. Target protospacer sequences analyzed in this study of the zebrafish

sgRNA	Target site sequence (5’-3’)
gdf6	ATGTCGATCAGAGAGGCCACTGG
ntl	GGAACCAGCCACCGACTGTTGG
dmd	ACAGTCAGCACTGCAAGACATGG
musk	CGGGATGGCATCATTTTCACTGG
rps14	GAAGAGCAGGTCATCAGCCTAGG
abcc4	TGGGTCGTACCTTGATCCTGCGG
psp1	GATGATCGAGGCCGGCCCACAGG
egfra	AAGATCAAAGTGCTGGGCTCCGG

Supplementary Table 5. QPCR primers used in this study. Oligonucleotides are synthesized at BioSune.

primers	sequence (5'-3')
Human β-Actin-QPCR-F	CACCAACTGGGACGACAT
Human β-Actin-QPCR-R	ACAGCCTGGATAGCAACG
HBG-QPCR-F	GGTTATCAATAAGCTCCTAGTCC
HBG-QPCR-R	ACAACCAGGAGCCTTCCCA
HBB-QPCR-F	TGAGGAGAAGTCTGCCGTTAC
HBB-QPCR-R	ACCACCAGCAGCCTGCCCA
