# Supplementary material for: Improving adenine and dual base editors through introduction of TadA-8e and Rad51DBD
Source: Nat Commun. 2023 Mar 3;14:1224. doi: 10.1038/s41467-023-36887-1 (PMC9984408; doi:10.1038/s41467-023-36887-1)
Supplement: Supplementary file 2 — Reporting Summary [file 41467_2023_36887_MOESM2_ESM.pdf]

nature portfolio

Corresponding author(s): Xiaohui Zhang, Tao P. Zhong and Dili Li

Last updated by author(s): Feb 19, 2023

Reporting Summary

Nature Portfolio wishes to improve the reproducibility of the work that we publish. This form provides structure for consistency and transparency in reporting. For further information on Nature Portfolio policies, see our Editorial Policies and the Editorial Policy Checklist.

Statistics

For all statistical analyses, confirm that the following items are present in the figure legend, table legend, main text, or Methods section.

n/a	Confirmed	
☐	☒	The exact sample size (*n*) for each experimental group/condition, given as a discrete number and unit of measurement
☐	☒	A statement on whether measurements were taken from distinct samples or whether the same sample was measured repeatedly
☐	☒	The statistical test(s) used AND whether they are one- or two-sided<br>*Only common tests should be described solely by name; describe more complex techniques in the Methods section.*
☒	☐	A description of all covariates tested
☒	☐	A description of any assumptions or corrections, such as tests of normality and adjustment for multiple comparisons
☐	☒	A full description of the statistical parameters including central tendency (e.g. means) or other basic estimates (e.g. regression coefficient) AND variation (e.g. standard deviation) or associated estimates of uncertainty (e.g. confidence intervals)
☐	☒	For null hypothesis testing, the test statistic (e.g. *F*, *t*, *r*) with confidence intervals, effect sizes, degrees of freedom and *P* value noted<br>*Give P values as exact values whenever suitable.*
☒	☐	For Bayesian analysis, information on the choice of priors and Markov chain Monte Carlo settings
☒	☐	For hierarchical and complex designs, identification of the appropriate level for tests and full reporting of outcomes
☒	☐	Estimates of effect sizes (e.g. Cohen's *d*, Pearson's *r*), indicating how they were calculated

Our web collection on statistics for biologists contains articles on many of the points above.

Software and code

Policy information about availability of computer code

Data collection	Targeted amplicon sequencing data were collected and demultiplexed by an Illumina HiSeq X Ten instrument.<br>RNA-seq data were collected and demultiplexed by an Illumina NovaSeq 6000 instrument.<br>FACS gating data were collected on a FACSAria III (BD Biosciences) using FACSDiva version 8.0.2 (BD Biosciences).
Data analysis	High-throughput sequencing data was analyzed by BE-Analyzer (http://www.rgenome.net/be-analyzer/#!)(Hwang G-H et al, BMC Bioinformatics, 2018) or CRISPResso2 (http://crispresso.pinellolab.partners.org/)(Clement, K. et al.Nat Biotechnol. 2019)for base editing (A>G,C>T,C>G and C>A) and indels efficiencies.<br>The custom script to analyze simultaneous A-to-G and C-to-T conversions induced by dual base editors is in https://zenodo.org/record/7635813#.Y-otL5PgQUs, or in Supplementary Software.<br>Potential DNA off-targets site for hyABE/hyA&C-BEmax were predicated using cas-OFFinder web (http://www.rgenome.net/cas-offinder/).<br>RNA-seq data were analyzed using Trim Galore (version 0.6.6), STAR (version 2.7.1a), SAMtools (version 1.14), Picard MarkDuplicates module (version 2.23.9) software.<br>FACS data was analyzed using FlowJo v.10.<br>Prism 9.3 was also used to analyze data.

For manuscripts utilizing custom algorithms or software that are central to the research but not yet described in published literature, software must be made available to editors and reviewers. We strongly encourage code deposition in a community repository (e.g. GitHub). See the Nature Portfolio guidelines for submitting code & software for further information.

Data

Policy information about availability of data

All manuscripts must include a data availability statement. This statement should provide the following information, where applicable:

- Accession codes, unique identifiers, or web links for publicly available datasets
- A description of any restrictions on data availability
- For clinical datasets or third party data, please ensure that the statement adheres to our policy

High–throughput sequencing reads have been deposited in the NCBI Sequence Read Archive under (PRJNA820131, PRJNA820322, PRJNA899988 and PRJNA925829). RNA-seq data have been deposited in the NCBI Sequence Read Archive database under accession code PRJNA818975, All Plasmids sequences or target site sequences mentioned in the manuscript are provided in supplementary information file. There are no restrictions on data availability.

Human research participants

Policy information about studies involving human research participants and Sex and Gender in Research.

Reporting on sex and gender	N/A
Population characteristics	N/A
Recruitment	N/A
Ethics oversight	N/A

Note that full information on the approval of the study protocol must also be provided in the manuscript.

Field-specific reporting

Please select the one below that is the best fit for your research. If you are not sure, read the appropriate sections before making your selection.

☒ Life sciences ☐ Behavioural & social sciences ☐ Ecological, evolutionary & environmental sciences

For a reference copy of the document with all sections, see nature.com/documents/nr-reporting-summary-flat.pdf

Life sciences study design

All studies must disclose on these points even when the disclosure is negative.

Sample size	No statistical methods were used to predetermine sample size. Experiments were performed in biological triplicate n=3 unless otherwise noted. Sample sizes were opted to display the range and consistency of differences and three biological replicates made it sufficient to support the conclusions in this research.
Data exclusions	No data were excluded from the analyses.
Replication	Three independent biological replicates were performed on different days.
Randomization	Samples were randomly distributed into groups.
Blinding	Investigators were not blinded to group allocation in this research since experimental conditions were evident and all samples of treatment were consistent throughout experiments.

Reporting for specific materials, systems and methods

We require information from authors about some types of materials, experimental systems and methods used in many studies. Here, indicate whether each material, system or method listed is relevant to your study. If you are not sure if a list item applies to your research, read the appropriate section before selecting a response.

Materials & experimental systems

n/a	Involved in the study
☒	☐ Antibodies
☐	☒ Eukaryotic cell lines
☒	☐ Palaeontology and archaeology
☐	☒ Animals and other organisms
☒	☐ Clinical data
☒	☐ Dual use research of concern

Methods

n/a	Involved in the study
☒	☐ ChIP-seq
☐	☒ Flow cytometry
☒	☐ MRI-based neuroimaging

Eukaryotic cell lines

Policy information about cell lines and Sex and Gender in Research

Cell line source(s)	HEK293T cells (source: ATCC CRL-3216)<br>HUDEP-2(Δ Gγ) cell line (University of New South Wales, Merlin Crossley lab)
Authentication	Cell lines were authenticated by the supplier.
Mycoplasma contamination	All cell lines used were tested negative for mycoplasma contamination.
Commonly misidentified lines (See ICLAC register)	No commonly misidentified cell lines were used.

Animals and other research organisms

Policy information about studies involving animals; ARRIVE guidelines recommended for reporting animal research, and Sex and Gender in Research

Laboratory animals	Wild-type AB zebrafish(Danio rerio) were purchased from the Institute of Hydrobiology of the Chinese Academy of Science(Wuhan, China) , raised and maintained at 28°C in an automatic fish housing system with 14h-10h light-dark cycle. About 50 pairs of male and female zebrafish (age from 4 months to 12 months) were used for crossing and generating embryos, embryos were used to microinject. Genomic DNA was extracted from pools of six injected embryos that developed normally at 48 hours post fertilization (hpf). The targeted genomic loci were amplified from genomic DNA and the PCR products were used for High-throughput sequencing.<br>Injected embryos that developed normally at 48hpf were fixed with 4% PFA and then conducted O-dianisidine staining and Whole-mount in situ hybridization.To maintain optical clearance, the embryos were treated with 0.003% phenylthiourea (PTU) to suppress pigmentation for analysis performed beyond 24 h post-fertilization (hpf).
Wild animals	No studies with wild animals were performed.
Reporting on sex	There is no sex based information in this study since at the developmental stages we performed the experiments the sex of embryo is not determined yet.
Field-collected samples	No studies with field-collected samples were performed.
Ethics oversight	All animal experiments were reviewed by the East China Normal University Center for Animal Research. All animal protocols were approved by IACUCs of East China Normal University.

Note that full information on the approval of the study protocol must also be provided in the manuscript.

Flow Cytometry

Plots

Confirm that:

☒ The axis labels state the marker and fluorochrome used (e.g. CD4-FITC).

☒ The axis scales are clearly visible. Include numbers along axes only for bottom left plot of group (a 'group' is an analysis of identical markers).

☒ All plots are contour plots with outliers or pseudocolor plots.

☒ A numerical value for number of cells or percentage (with statistics) is provided.

Methodology

Sample preparation	Cell culture and transfection procedures are described in the online methods. Cells were washed and filtered through a 45μm cell strainer cap before sorting (72h after transfection).

Instrument	FACSAria III (BD Biosciences)
Software	BD FACSDiva Software Diva8.0.2
Cell population abundance	HEK293T Cell population abundances after gating for target populations were similar in different biology replicates. HEK293T cells infected with base editors described in the supplement usually were ~ 60-75% GFP+ (of gated population = % parent in BD FACSDiva).
Gating strategy	For HEK293T cells, gates were established using uninfected control cells and GFP positive control. Gates were drawn to collect subsets of GFP-expressing cells. Cells with top 15% of GFP signal were sorted, after gating for the cell population (~15% of parent). Exemplifying the gating strategy is provided in the Supplementary Note.

☒ Tick this box to confirm that a figure exemplifying the gating strategy is provided in the Supplementary Information.
