# Supplementary material for: Improving adenine and dual base editors through introduction of TadA-8e and Rad51DBD
Source: Nat Commun. 2023 Mar 3;14:1224. doi: 10.1038/s41467-023-36887-1 (PMC9984408; doi:10.1038/s41467-023-36887-1)
Supplement: Supplementary file 3 — Description of Additional Supplementary Files [file 41467_2023_36887_MOESM3_ESM.pdf]

Title: Supplementary Software.
Description: The custom script to analyze simultaneous A-to-G and C-to-T conversions induced by dual base editors.

BE editing efficiency analysis pipeline
This code follows the analysis process of BE-analyzer and suit for batch analysis.

prerequisite
* fastq-join, Version: 1.3.1
* blat, Version: 35
* SeqKit, Version: 2.1.0
* EMBOSS, Version: 6.6.0.0

usage example
in shell
bash BE-efficiency.sh

Title: Supplementary Data 1.
Description: Target protospacer sequences and corresponding oligonucleotides used in this study in HEK293T cells. Oligonucleotides are generally synthesized at BioSune unless otherwise stated.

Title: Supplementary Data 2.
Description: PCR primers used in this study. Primers are generally synthesized at BioSune unless otherwise stated.

Title: Supplementary Data 3.
Description: Off-target sites and PCR primers used in this study. Primers are generally synthesized at BioSune unless otherwise stated.
